# Video-based telemedicine utilization patterns and associated factors among racial and ethnic minorities in the United States during the COVID-19 pandemic: A mixed-methods scoping review

**DOI:** 10.1371/journal.pdig.0000952

**Published:** 2025-07-24

**Authors:** John M. Meddar, Ratnalekha V. N. Viswanadham, Defne L. Levine, Tiffany R. Martinez, Kendra Willis, Noah Choi, Jackson Douglas, Katharine S. Lawrence

**Affiliations:** 1 Department of Population Health, New York University Grossman School of Medicine, New York, New York, United States of America; 2 Department of Pediatrics, New York Presbyterian Hospital (Cornell Campus), New York, New York, United States of America; 3 Great Neck North High School, Great Neck, New York, United States of America; 4 College of Engineering, Cornell University, Ithaca, New York, United States of America; Shahid Beheshti University of Medical Sciences School of Dentistry, IRAN, ISLAMIC REPUBLIC OF

## Abstract

The COVID-19 pandemic catalyzed a rapid expansion of telemedicine across the United States, expanding access to video-based services but also raising concerns about equitable access, use, and experience among minority populations. This mixed-methods scoping review quantitatively describes patterns of video-based telemedicine utilization and qualitatively evaluates factors impacting utilization among racial/ethnic minorities in the United States during the COVID-19 pandemic. We conducted a comprehensive literature search across six databases for studies published between January 2020 and March 2023. Eligible studies reported on telehealth or telemedicine use, specifically video-based visit utilization among racial/ethnic minorities. Reviewers independently screened studies, extracted data, and synthesized findings using an integrated mixed-methods approach. Of 1801 studies, 77 studies met the inclusion criteria. Of these, a majority were published in metropolitan coastal areas, and most were heterogeneous in their definition of telemedicine and utilization. Quantitatively, 33 studies (42.9%) reported increased use of video-based telemedicine, 29 (37.7%) reported decreased use, and 15 (20%) reported variable use across racial/ethnic subgroups. Most studies assessed disparities among non-Hispanic Black and Hispanic/Latinx populations (73 and 66 studies, respectively), while fewer examined disparities among other minority populations (45 studies). Factors associated with telemedicine adoption included patient- and community-level digital access barriers, low organizational digital capacity and infrastructure, implicit bias, and inadequate provider education and training. Identified facilitators included trust and awareness of telemedicine, adequate provider training, cultural and linguistic adaptations, targeted internet subsidies, and telemedicine reimbursements. Video-based telemedicine utilization among racial/ethnic minorities during the COVID-19 pandemic was heterogeneous, influenced by individual, systemic, and implementation factors. Disparities were most pronounced among Asians and other minority populations. Despite increased attention and efforts to address access barriers, our findings highlight the need for more targeted, culturally and structurally tailored interventions to improve digital inclusion.

## Author summary

The COVID-19 pandemic accelerated the adoption of telemedicine in the U.S, which negatively impacted racial/ethnic minorities who struggled with unequal access and use of digital technologies. In this study, we analyzed literature from the pandemic, examining unequal use of telemedicine among racial/ethnic minority groups. Seventy-seven studies met our inclusion criteria and findings were summarized based on the reported frequency of telemedicine use or proportional use compared to non-Hispanic Whites. We compared telemedicine use across the following visit types: video, telephone and in-person visits. Our findings showed a greater number of studies reported increased telemedicine (video and/or telephone) use among non-Hispanic Black and Hispanic populations, while more studies reported decreased use among Asians and other minority populations. We also examined factors that influenced telemedicine adoption across studies. Identified factors included access to digital technologies, digital literacy, providers’ racial bias and attitudes towards telemedicine, institutional readiness to adopt and effectively use telemedicine, community outreach, and policies influencing digital expansion and telemedicine reimbursements. Our findings highlight uneven telemedicine use patterns among minority populations and a multitude of factors that influenced these patterns. Effective strategies are needed across various levels of healthcare delivery to improve digital inclusion of minority populations.

## Introduction

The recent COVID-19 pandemic introduced unprecedented digital changes to the U.S. healthcare delivery system [1,2]. These changes required digital devices, platforms, and solutions to access and utilize healthcare delivery services—a revolution in “virtual” service delivery. However, widespread digital transformations resulted in access and utilization barriers for vulnerable populations, including racial/ethnic minorities [1,3,4].

It is well established that inequitable access to internet-enabled technologies during the pandemic, including smartphones, laptops, computers, and broadband connection, precipitated unequally negative outcomes for minority groups across various mobility domains, including health [5,6], education [7,8], banking [9,10], and legal systems [11]. In the health domain, minority populations were disproportionately impacted by COVID-related hospitalization and mortality outcomes, which were exacerbated by inequitable access and use of digitally mediated healthcare [12,13]. However, recent findings from the pandemic literature show mixed telemedicine utilization among racial/ethnic minorities, suggesting that telemedicine use is highly nuanced and requires additional investigation [14–18].

Telemedicine care offers several benefits to patients facing access and utilization barriers to healthcare, including remote physical examinations and more connected care compared to telephone visits [19]. Video-based visits also increase access to care for patients who experience physical, work, and transportation difficulties [20–22]. While the benefits of video-based telemedicine visits, the principal conceptual focus of this study, are well described, racial/ethnic minorities are disproportionately excluded from accessing its benefits [23–25]. Other studies from the pandemic literature report increased telephone uptake among racial/ethnic groups due to ease of use relative to video-based visits [26,27]. However, telephone visits hinder access to care for patients with language barriers who are unable to utilize visual and nonverbal forms of communication compared to patients who use video-based visits [28]. In contrast, additional studies report greater uptake of in-person visits after the lockdown from April to June 2020, attributed to patient trust and personal preference, shifting in favor of in-person visits [29–32]. Factors associated with variability in telemedicine use were broadly described across the following categories: geographic [33], specialty line [14,34,35], patient [36–38], and operational-specific factors [15,39].

A recent scoping review analyzing pandemic telemedicine use disparities reported no variations in geographical settings and clinical specialties concerning trends in utilization [40]. While studies show that rural populations utilize telemedicine more due to geographic distance from brick-and-mortar clinical settings [41], substantial research shows that rural populations lack quality internet access due to pervasive gaps in digital infrastructure [42,43]. Furthermore, existing telemedicine research suggests that adoption and utilization among racial/ethnic minorities vary across lines of specialty practices [44,45], mirroring underlying inequities in the broader healthcare delivery system. Inconclusive findings warrant a more comprehensive assessment of the existing literature landscape, including a qualitative evaluation of the factors that drive adoption and utilization practices among racial/ethnic minorities. For instance, a narrow body of research has qualitatively characterized factors associated with telemedicine use, such as barriers and facilitators, at varying socio-ecological levels of care, including at the individual, provider, organizational, community, and policy levels.

The present study provides a quantitative overview of synchronous telemedicine utilization patterns, with a focus on video-based visits, among racial/ethnic minorities in the US during the pandemic. This study also qualitatively evaluates factors that promoted or hindered these patterns. Given increased technology acceptance and adoption among racial/ethnic minorities in recent years [46,47], this work lays an essential groundwork for systematic improvements focused on enhancing and sustaining telemedicine adoption and utilization practices among racial/ethnic minorities, particularly in preparation for future pandemics and as the healthcare delivery system becomes increasingly digitized.

## Materials and methods

### Research question and terminology

We proposed the following questions for our scoping review: (1) How did video-based telemedicine visit utilization–defined as the proportion of telemedicine use, as reported using descriptive or inferential statistics, by patients to obtain medical services from a clinical provider– among racial/ethnic minority populations compared to in-person visits during the pandemic? (2) What was the distribution of clinical specialties and geographic locations involved? (3) What are the barriers and facilitators of telemedicine utilization among racial/ethnic minorities?

We utilized the Arksey and O’Malley methodological framework for scoping reviews [48]. We systematically mapped, synthesized, and reported study findings in alignment with the PRISMA Extension for scoping reviews [49]. Given the limited consensus on an appropriate and well-defined definition in the existing literature, we used the American Academy of Family Medicine (AAFP) definitions of “telehealth” and “telemedicine”, focusing on video-based (or video-first, in cases where mixed modalities were present) platforms as these represent the most novel technology shift seen during the pandemic (versus phone calls) [50]. Since telehealth and telemedicine are often used interchangeably, we operationalized telemedicine as the operative term to analyze, describe, and report on study findings. (Table 1) We define race/ethnicity using the U.S. Census Bureau’s definitions as “a person’s self-identification with one or more social groups” [51]. We prioritized race/ethnic minority populations for this review due to persistent concerns regarding disparities in healthcare access and utilization among racial/ethnic minority groups and the increased potential to exacerbate them by virtualizing healthcare delivery processes [52,53]. Finally, the term “utilization” was derived from the broader MeSH term “Health Services Accessibility” [54], which encapsulates a wider ethos of service delivery utilization terminologies, including telehealth access, adoption, use, and utilization across the health services literature. We defined utilization as the proportion of telemedicine used, reported using descriptive or inferential statistics, across race/ethnic minority groups, to assess utilization differences based on quantitative results presented in the literature. Our study period ranged from 2020 to 2023, reflecting a period of concentrated digital transformation across the healthcare system [3].

**Table 1:** Definitions, inclusion criteria, and exclusion criteria used to define the scoping review and analyses.

Technology interventions	
*Telehealth (AAFP)*	A broad collection of electronic and telecommunications technologies and services that support at-a-distance healthcare delivery and services. Telehealth technologies and tactics support virtual medical, health, and education services.
*Telemedicine (AAFP)*	The practice of medicine using technology to deliver care at a distance, over a telecommunications infrastructure, between a patient at an originating (spoke) site and a physician, or other practitioner licensed to practice medicine, at a distant (hub) site.
*Telemedicine (PubMed MESH)*	Delivery of health services via remote telecommunications, which includes interactive consultative and diagnostic services.
**Health disparity population**	
*Race and/or ethnicity (US Census Bureau)*	non-Hispanic White, non-Hispanic Black or African American, Hispanic/Latinx, Asian, Native American and Alaska Native (NA/AN), Native Hawaiian and Pacific Islander (NH/PI).
**Outcomes of interest**	
*Utilization, Use*	The proportion of telemedicine use reported, using descriptive or inferential statistics, across racial/ethnic minority subgroups.
*Health Services Accessibility (MESH)*	The degree to which individuals are inhibited or facilitated in their ability to gain entry to and to receive care and services from the health care system. Factors influencing this ability include geographic, architectural, transportation, and financial considerations.
**Time Period**	
*COVID-19 pandemic*	2020-2023
**Included studies**	Study designs: Observational analyses, case reports, mixed methods papers (for quantitative sections), cross-sectional studies, panel studies, survey studies. U.S.-based studies that were conducted and published between 2020 and 2023 Studies with patients of all ages Studies with a sample size of ≥ 30% race/ethnicity Studies explicitly focused on disparities in telemedicine utilization. Any demographic setting (rural, urban, or suburban) All practice types. All sexes and genders Studies were eligible for inclusion if they involved the synchronous provision of care (patient-provider interactions) using telecommunication and/or information technology. Studies that compared telemedicine utilization to other modalities (i.e., telephone and in-person visits) Studies that examined patient-provider interactions via video-based visits or a combination of video, telephone, and in-person visits.
**Other exclusions**	Non-English papers International papers Studies that did not fall within the predefined time window for the review. Interventions that were asynchronous and did not involve provider-patient interactions. Studies that examined patient-provider interactions, but to evaluate exclusive telephone visit uptakes, making appointments, taking medications, and other administrative reasons. Studies that did not examine the utilization of telehealth and telemedicine. Studies that did not measure or assess utilization and did not stratify findings by racial or ethnic minorities. Studies that were not peer-reviewed or did not have a quantitative analysis (commentaries, perspectives, qualitative studies, formal review papers) Randomized controlled trials (RCTs) were also excluded, with the assumption that the controlled environment of an RCT, where digital technologies are provided by study staff, does not lend itself to understanding prevalent patterns of utilization among racial and ethnic minority groups.

### Search strategy and screening

A medical librarian (DR) conducted a comprehensive literature search in December 2023 across six databases: PubMed, Embase, Cochrane Library, Web of Science, CINAHL, and Engineering Village. The search strategy incorporated controlled vocabulary and keywords related to “telemedicine utilization,” “telehealth,” “disparities,” and “racial/ethnic minorities.” Additional studies were identified by screening the reference lists of prior relevant reviews. For more details on our search strategy, please refer to S4 File.

### Inclusion

We included studies that met the following criteria: observational studies (including retrospective and prospective cohort studies, cross-sectional evaluations, and case studies) conducted in the United States (U.S.) and explicitly focused on evaluating sociodemographic disparities in telemedicine utilization, particularly among racial/ethnic minorities. Furthermore, studies were required to have analyzed data directly or indirectly from patient-provider interactions within the clinical setting (e.g., EHR data, claims data, self-reported telemedicine use data, such as population-based surveys). Given our explicit interest in understanding video-based telemedicine use dynamics relative to other visit modalities, studies must have utilized and performed direct comparisons between video-based visits versus telephone and in-person visits. To closely approximate the racial/ethnic composition of the U.S., studies were only included if they had minority representation of at least 30% in their race or ethnicity [55]. This threshold reflects national racial/ethnic distributions and reduces selection bias in studies that may disproportionately include white patients. We included individuals of all ages, sexes, geographic types (urban, suburban, and rural), clinical conditions, and clinical practice types. Asynchronous studies that did not assess visits at the provider-patient level, did not directly compare visit modalities, were not conducted in the US, were not written in English, or were not peer-reviewed were excluded. Additionally, randomized controlled trials (RCTs) were excluded since the controlled conditions in an RCT do not reflect the underlying telemedicine adoption patterns of the general U.S. population.

We used Covidence, an internet-based software tool developed by Cochrane, to screen publications and extract key parameters [56]. JMM, RVNV, TRM, DLL, and KW conducted literature screening across two successive stages: abstract and title screening, and full-text screening. JMM, RVNV, NC, JD, and DLL completed data extraction with consensus. Emerging conflicts were resolved among reviewers. Table 1 details the definitions used for paper screening, the study’s examination period, and the inclusion and exclusion criteria.

### Data extraction

The primary aim of data extraction was to systematically and uniformly extract data from each study, thereby facilitating information synthesis and dissemination. Reviewers assessed the final selection of eligible studies and extracted data based on the following study parameters: author, year of publication, objective, study design, treatment modality, geographical location, medical specialty, medical condition, telehealth/telemedicine utilization by race/ethnicity, relevant results, and conclusion. The data extracted were inserted into our internally developed extraction tool. We utilized the built-in functionalities provided in Covidence to increase consensus. This process required at least two reviewers to agree on the data extracted from each study. An ad hoc extraction process was employed to extract data on additional study parameters after completing the Covidence extraction process.

### Data analysis

#### Quantitative analysis.

We analyzed a subset of studies to identify telemedicine adoption patterns. This evaluated variability across study populations or consistencies according to US racial demographic breakdowns. If patterns were not identifiable, we examined the frequency of occurrence across the extracted study aspects, including sample size, race/ethnicity of participants, study design, study duration, terminology definition, medical specialties, medical conditions, geographic distribution, unit of analysis, sample size, and pre-post analysis. Telemedicine utilization was assessed by examining the proportion of uptake using frequencies, percentages, or measures of association (i.e., odds ratios and risk ratios) for each group across three distinct visit modalities: video, telephone, and in-person visits. If frequency was not used, we used OR and RR (which had a comparative group of White). For frequency, we compared occurrence and percentage distributions across the modality categories. If the study had a statistical test, we examined statistical significance.

**Increased uptake**: If increased video-based telemedicine, telephone, or in-person visits were observed across all groups, we defined it as “increased uptake.”

**Mixed uptake:** If utilization varied across groups, it was defined as “mixed uptake.”

**Decreased uptake**: If in-person visits were greater than those for video-based telemedicine and telephone visits, we defined it as “decreased uptake.”

We assessed uptakes across three categories: video-based telemedicine versus in-person visits, video-based telemedicine versus telephone visits versus in-person visits, and telemedicine (including video and telephone visits) versus in-person visits.

#### Qualitative analysis.

To expand on our quantitative analysis, our qualitative analysis aimed to identify factors that influenced the adoption of telemedicine among racial/ethnic minorities during the pandemic [14,15,28,34–39,56–67]. We purposively reviewed the qualitative content of included studies for information richness and selected a subset (n = 20), which provided sufficiently robust descriptions of factors associated with telemedicine use and were representative across geography, specialty practice types, and race/ethnicity groups. We determined information saturation based on the following limitations observed across studies (1) many studies had robust descriptions of factors that moderated telemedicine utilization, but had a high degree of redundancy in results, discussion, and contextualization of existing literature, (2) studies provided limited descriptions of inequities among racial/ethnic populations and were primarily focused on disparities across other essential demographic characteristics, including age, sex, income, geography, etc.,(3) studies were primarily or exclusively quantitative and did not provide sufficient discussion of factors that impacted telemedicine utilization.

For our qualitative synthesis, we performed a systematic thematic analysis [68], comprised of the following steps: (1) descriptive texts that best characterized our research objective were identified and color-coded, (2) keywords and terms that frequently recurred in the text were selected as they emerged, (3) exemplary words, terms, or phrases were coded and inserted into an informal codebook, (4) codes were organized into groups that facilitated meaningful data interpretation to stimulate broader thematic development, (5) conceptual interpretation of emerging keywords, codes, and themes was performed to contextualize and concretize concepts derived from data interpretation, and (6) a theoretical framework was developed to organize emerging concepts into a cohesive theoretical structure [69,70]. A socio-ecological theory [71] was employed to organize facilitative and prohibitive concepts of telemedicine adoption across the following socio-ecological levels of care: individual, provider, organization, community, and policy. Findings from our analysis are collated, summarized, and graphically illustrated in the Results section.

#### Inter-rater and coding reliability.

Inter-rater reliability (IRR) was 0.54 for abstract and title screening and 0.35 for full-text screening. Covidence did not generate an IRR for data extraction. All conflicts were resolved amongst reviewers to ensure consistency, as Covidence required perfect agreement across each stage of the screening and extraction process [72]. Quantitative data elements such as unit of analysis, study period duration, utilization across differentiated study periods, and sample size were independently extracted, outside of our formalized data extraction process in Covidence (JMM). Additionally, we used a single-coder process in the qualitative evaluations of this review, given the clearly defined and easily identifiable themes that emerged in the literature (JMM).

## Results

### Overview

**Fig 1:**
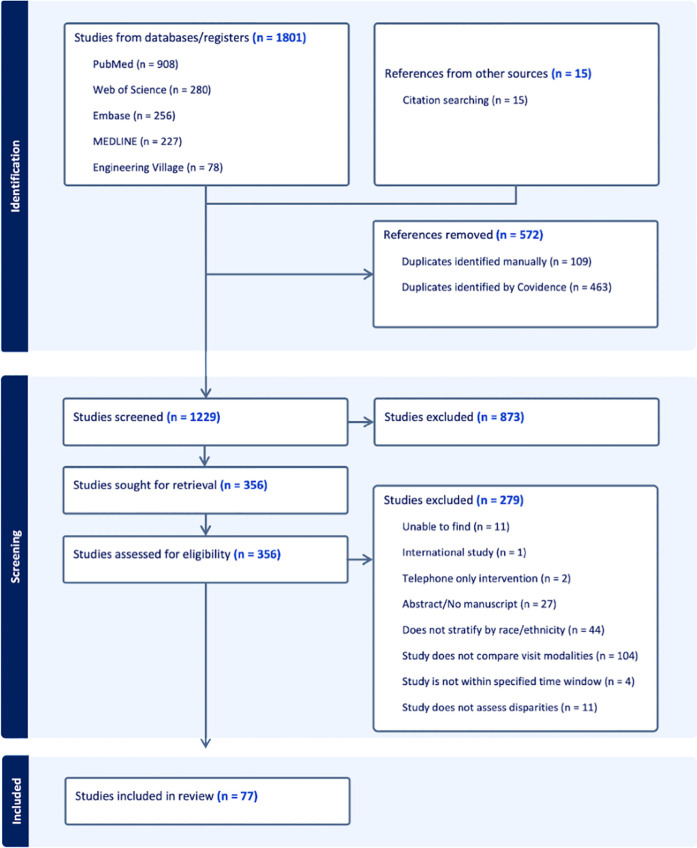
PRISMA diagram showing the exclusion of studies deemed ineligible for inclusion and the corresponding reasons, leading to a final pool of 77 included studies.

A total of 1,786 studies were identified in the initial search, downloaded into Covidence, and screened by reviewers (Fig 1). Of these, 77 studies met our inclusion criteria. Across included studies, the majority analyzed patient-level data, representing diverse racial/ethnic minority groups and various study designs. Table 2 briefly overviews the study characteristics. A majority of studies analyzed patients (n = 53, aggregate patient count: n = 7,937,340), though a significant number analyzed clinical encounters (n = 42, aggregate encounter count: n = 6,963,693). The racial/ethnic minorities breakdown across included studies were: non-Hispanic/Latinx Black (n = 73), Hispanic/Latinx (n = 66), Asians (n = 45), Native American and Alaskan Native (NA/AN) (n = 18), and Native Hawaiian and Pacific Islander (NH/PI) (n = 13). A majority of studies had a cohort design (n = 46). A minority of studies had a cross-sectional design (n = 31).

**Table 2:** Key study characteristics and features extracted from included studies.

Author (year)	Study design	Unit of analysis	Sample size	Geo-graphic region	State	Clinical specialty	Race/ethnicity groups included	Modalities compared	Telemedicine Utilization
Almandoz et al., 2023 [73]	Cross-sectional	Patient visits	583	South	Texas	Obstetrics	NHB, H/L	Video and telephone and In-person	More use by NHB
Anderson et al., 2023 [74]	Cohort	Patient visits	212	Northeast	Connecticut	Pediatric neurosurgery	NHB, H/L, AS	Video and telephone and In-person	Less use by H/L
Annapragada et. al., 2021 [75]	Cohort	Patient visit	13,979	Northeast	Maryland	Surgery	NHB	Video and telephone and In-person	No difference
Brown et al., 2021 [76]	Cross-sectional	Patient visits	73,465	South	Multi-state	Pediatrics	NHB, H/L	Video, In-person, and Telephone	Less use by NHB and H/L
Bustamante et al., 2023 [57]	Cohort	Encounter visits	118,963	West	California	Multiple specialties	NHB, H/L, AS	Video, In-person, and Telephone	Less use by NHB and H/L
Campos-Castillo et al., 2021 [77]	Cross-sectional	Patient visits	10,624	National	National	Multiple specialties	NHB, H/L	Video, In-person, and Telephone	More use by NHB and less use by H/L
Campos-Castillo., 2022 [78]	Cross-sectional	Patient visits	532	National	National	Behavioral health	NHB, H/L	Video, In-person, and Telephone	Less use by NHB and more use by H/L
Chen et al., 2022 [58]	Cross-sectional	Patient visits	5,023	Northeast	Connecticut	Ophthalmology	NHB, H/L	Video, In-person, and Telephone	Less use by NHB and H/L
Chen et al., 2023 [59]	Cohort	Patient visits	247,287	Northeast	New York	Primary care	NHB, H/L, AS	Video, In-person, and Telephone	Less use across all race/ethnicity groups
Childs et al., 2021 [79]	Cohort	Patient visits	1,008	Northeast	Connecticut	Psychiatry	NHB, H/L	Video, In-person, and Telephone	Less use by H/L and no change for NHB
Chumbler et al., 2023 [60]	Cohort	Patient visits	13, 962	South	Tennessee	Ambulatory care	NHB, H/L	Video and Telephone	More use by H/L
Chunara et al., 2021 [15]	Cohort	Patient visits	494,322	Northeast	New York	Multiple specialties	NHB, H/L, AS	Video and In-person	Less use by NHB
Cordasco et al., 2022 [80]	Cross-sectional	Patient visits	13,469	Multi-regional	Multi-state	Urgent care	NHB, H/L	Video, In-person, and Telephone	More use by NHB
Cousins et al., 2022 [61]	Cohort	Patient visits	62,172	Midwest	Michigan	Oncology	NHB, H/L, AS, NA/AN	Video, In-person and Telephone	Mixed use depending on subspecialities
D’Amico et al., 2023 [30]	Cohort	Encounter visits	129,816	Midwest	Ohio	Primary care	NHB, AS, NA/AN, NH/PI	Video, In-person, and Telephone	No difference for NHB and less video visit use across all other groups
Darrat et al., 2021 [81]	Cohort	Patient visits	1,162	Midwest	Michigan	Otolaryngology	NHB	Video, In-person, and Telephone	Less use by NHB
Der-Martirosian et al., 2022 [82]	Cohort	Patient visits	113,091	West	California	Multiple specialties	NHB, H/L	Video, In-person, Telephone, Video and telephone	More use by NBH and less use by H/L
Drake et al., 2022 [29]	Cohort	Encounter visits	624, 886	South	North Carolina	Multiple specialties	NHB, AS NA/AN, NH/PI	Video, In-person, and Telephone	Less use by NHB, H/L and AS
Duan et al., 2022 [62]	Cross-sectional	Encounter visits	1,444	Midwest	Illinois	Specialty care	NHB, H/L, AS	Video and In-person	Less use by NHB and no difference for H/L
Eberly et. 2022 [83]	Cohort	Patient visits	148,402	Northeast	Multiple states	Multiple specialties	NHB, H/L, AS	Video, in-person, and telephone	Less use by AS and more use by NHB and H/L
Ekwegh et al., 2023 [63]	Cross-sectional	Patient visits	150	West	California	Multiple specialties	NHB	Video, In-person, and Telephone	No difference
Ennis et al., 2022 [64]	Cross-sectional	Patient visits	4,873	South	Multiple states	Primary care	NHB, H/L	Video and Telephone	Less use by NHB and H/L
Eruchalu et al., 2022 [34]	Cohort	Encounter visits	5,908	Northeast	Massachusetts	Surgery	NHB, H/L	Video, In-person, and Telephone	Less use by NHB
Esper et al., 2021 [14]	Cross-sectional	Encounter visits	686	South	Georgia	Neurology	NHB; AS	Video, In-person, and Telephone	Less use by NHB and AS
Flowers et al., 2023 [84]	Cohort	Patient visits	13,608	Northeast	Rhode Island	Behavioral health	NHB; H/L	Video, In-person, Video and telephone	More use by H/L and less use by NHB
Freed et al., 2023 [85]	Cohort	Encounter visits	63,665	South	North Carolina	Multiple specialties	NHB, H/L	Video, In-person, and Telephone	Less use by NHB and H/L
French et al., 2023 [86]	Cohort	Patient visits	54,996	South	North Carolina	Behavioral health	NHB, H/L	Video, In-person, Telephone, Video and telephone	Less use by NHB and H/L
Friedman et al., 2022 [36]	Cross-sectional	Patient visits	856	Midwest	Illinois	Infectious diseases	NHB	Video, In-person, and Telephone	Less use by NHB
Gao et al., 2022 [31]	Cross-sectional	Patient visits	2,521	South	Tennessee	Obstetrics	NHB, H/L, AS	Video, In-person, and Video and telephone	Less use by NHB; H/L
Govier et al., 2022 [65]	Cohort	Patient visits	11,326	Multi-regional	Multi-State	Multiple specialties	NHB, H/L, AS, NA/AN, NH/PI	Video, In-person, and Telephone	More use by NHB and less use by H/L
Grefe et al., 2023 [35]	Cohort	Patient visits	1,250	South	Multi-state	Neurology	NHB, H/L	Video, In-person, and Telephone	Less use by NHB and H/L
Haynes et al., 2021 [87]	Cohort	Patient visits	1,292	West	California	Endocrinology	NHB, H/L, AS, NA/AN, NH/PI	Video, In-person, and Telephone	No difference
He et al., 2023 [88]	Cross-sectional	Patient visits	3,621	National	National	Multiple specialties	NHB, H/L	Video, In-person, and Telephone	Less video use by NHB and H/L
Hill et al., 2021 [89]	Cross-sectional	Encounter visits	3,142	South	Multi-state	Reproductive health	NHB, H/L	Video, In-person, and Telephone	Less use by NHB and no difference for H/L
Huang et al., 2022 [32]	Cohort	Patient visits	64,011	West	California	Multiple specialties	NHB, H/L, AS	Video, In-person, and Video and telephone	No differences across hospitalized groups and more use among non-hospitalized AS
Jallow et al., 2022 [66]	Cohort	Encounter visits	12,620	Northeast	District of Columbia	Dermatology	NHB, H/L, AS	Video, In-person, and Telephone	More use by AS and less use by NHB and H/L
Khatana et al., 2022 [90]	Cross-sectional	Patient visits	1,999,534	National	National	Multiple specialties	NHB, H/L	Video, In-person, and Video and telephone	Less use by NHB and H/L
Kolb et al., 2021 [39]	Cross-sectional	Encounter visits	7,392	Northeast	Delaware	Otolaryngology	NHB, AS, NA/AN, NH/PI	Video, In-person, and Telephone	Less use across all race/ethnicity groups
Kummer et al., 2022 [38]	Cohort	Patient visits	14,170	Northeast	New York	Neurology	NHB, AS, NA/AN, NH/PI	Video, In-person, and Telephone	Less use across all race/ethnicity groups
Kusters et al., 2023 [91]	Cohort	Patient visits	1, 754 311	National	National	Behavioral Health	NA/AN	Video, In-person, and Telephone	Less use by rural NA/AN
Lamb et al., 2022 [92]	Cross-sectional	Patient visits	5,717	Northeast	Pennsylvania	Dermatology	NHB, H/L, AS	Video and Telephone	More video visit use across all groups
Lambert et al., 2021 [16]	Cross-sectional	Patient visits	682	Northeast	Connecticut	Pediatric neurosurgery	NHB, H/L	Video, In-person, and Telephone	Less use by NHB and H/L
Lee et al., 2023 [93]	Cohort	Patient visits	1,067,798	National	National	Multiple specialties	NHB, H/L, AS	Video, In-person, Telephone, and Video and telephone	More telephone use by NHB
Lin et al., 2023 [37]	Cross-sectional	Patient visits	6,967	National	National	Behavioral health	NHB, H/L, AS	Video, In-person, and Video and telephone	No differences
Lott et al., 2021 [94]	Cohort	Encounter visits	5,035	Northeast	New York	Orthopedics	NHB, AS, NA/AN, NH/PI	Video and In-person	Less use by NHB, H/L and AS
Mahmud et al., 2020 [95]	Cohort	Encounter visits	11,828	Multi-regional	National	Hepatology	NHB, H/L, AS	Video, In-person, Video and telephone	No differences
Merz-Herrala et al., 2023 [96]	Cohort	Patient visits	2,031	National	National	Reproductive health	NHB, H/L, AS, NA/AN	Video, In-person, and Video and telephone	Less use across all race/ethnicity groups
Mueller et al., 2022 [28]	Cohort	Patient visits	3,314	Northeast	New York	Multiple specialties	NHB, H/L, AS	Video, In-person, and Telephone	Less use by NHB and H/L
Naqvi et al., 2023 [97]	Cross-sectional	Encounter visits	2,024	Multi-regional	Multi-state	Multiple specialties	NHB, H/L, AS	Video, In-person, and Telephone	Less use by NHB and H/L
Neeman et al., 2022 [67]	Cohort	Patient visits	46,052	West	California	Oncology	NHB, H/L, AS, NH/PI	Video, In-person, and Telephone	More telephone use across all race/ethnicity groups
Osmanlliu et al., 2023 [98]	Cohort	Encounter visits	113,888	West	California	Cardiology	NHB, H/L, AS	Video, In-person, Telephone, and Video and telephone	Less use by NHB and H/L
Pagan et al., 2020 [99]	Cohort	Patient visits	19,376	West	California	Primary care	NHB, AS, NA/AN; NH/PI	Video, In-person, and Telephone	Less video use by NHB, H/L and AS
Palzes et al., 2023 [100]	Cohort	Patient visits	36,607	West	California	Multiple specialties	NHB, H/L, AS, NA/AN, NH/PI	Video, In-person, and Video and telephone	No differences
Pitaro et al., 2022 [101]	Cohort	Patient visits	3,090	Northeast	New York	Multiple specialties	H/L	Video, In-person, and Video and telephone	Less use by H/L
Pritchett et al., 2022 [102]	Cross-sectional	Patient visits	101,756	Multi-regional	Multi-state	Multiple specialties	H/L	Video, In-person, and Video and telephone	Less use by H/L
Rametta et al., 2020 [103]	Cohort	Encounter visits	17,369	Northeast	Pennsylvania	Neurology	NHB, H/L, AS	Video and telephone and In-person	Less video visit use across all race/ethnicity groups

Table 2 summarizes key study characteristics and results from included studies. The extracted parameters bridge a connection between our research questions and the insights synthesized in this review by visually depicting specific and explanatory data elements drawn from each study.

NHB, non-Hispanic Blacks; H/L, Hispanics/Latinx; As, Asians; NA/AN, Native American/Alaska Native; NH/PI, Native Hawaiian/ Pacific Islander.

Across all studies, the average study period was 8.12 months (SE = 0.79 months), with a median of 6.30 months (IQR = [3.30, 10.0]). A minority collected data before and during the pandemic (n = 17). We observed varied definitions for telemedicine and telehealth: slightly more studies used “telehealth” (n = 36) compared to “telemedicine” (n = 11), with some using terms interchangeably. The full table of included studies can be seen in the S1 File.

### Clinical specialties and conditions included in studies

Minority Video-based telemedicine adoption among minority populations across clinical specialties and conditions included in studies during the acute pandemic. Among studies that examined race/ethnic disparities in telemedicine uptake, single specialty care specialties were the most significantly represented (n = 30) followed by multiple specialty care (n = 23) and primary care (n = 14) Other studies evaluated race/ethnic minority telemedicine utilization and adoption in behavioral health (n = 5) and surgery (n = 5). A majority of studies examined multiple conditions (n = 55) versus a single condition (n = 20). A minority of studies were unclear about the conditions studied (n = 2).

### Geographical distribution of studies

**Fig 2:**
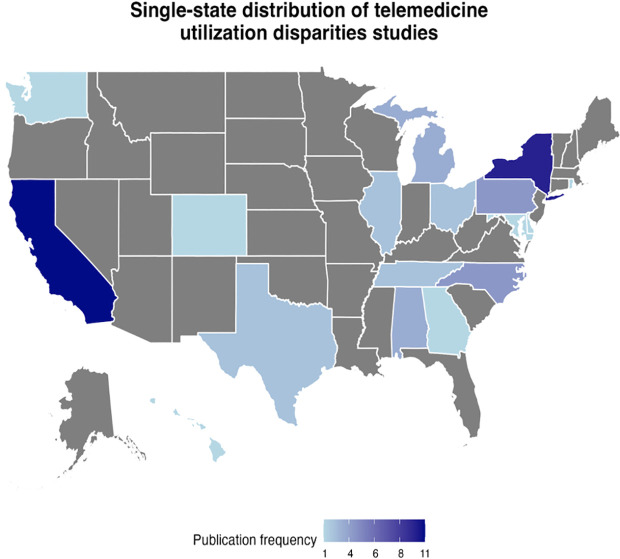
The single-state frequency distribution of telemedicine utilization studies featured in the review, with California and New York having the highest publication frequencies of all states. Studies that were nationally representative, multi-regional, and cross-institutional collaborations were excluded from the figure.

The included studies primarily represented metropolitan areas, particularly along the East Coast, with less representation from rural regions. Most studies were conducted in the Northeast (n = 27), followed by the South (n = 16), the West (n = 14), multi-regional sites (e.g., using data from national surveys, networks of healthcare sites, or cross-institutional collaborations (n = 13) and the Midwest (n = 7). The state with the highest single-state representation across studies was California (n = 11), followed by New York (n = 10). Other studies were national (n = 9) or multi-state representative studies (n = 9). This representation can be found in Fig 2.

### Video-based telemedicine adoption among minority populations

**Fig 3:**
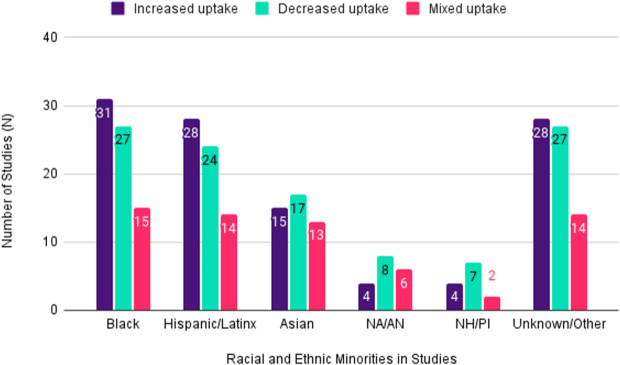
Highlights significant differences in telemedicine uptake by racial/ethnic group, with Black and Hispanic/Latinx populations showing the highest variability in adoption patterns.

Telemedicine adoption patterns differed across racial/ethnic groups, with increased uptake more often reported among Black and Hispanic/Latinx patients and mixed or decreased uptake among Asian, Native American/Alaska Native, and Native Hawaiian/Pacific Islander groups. We classified the results of included studies based on whether telemedicine or video visit uptake increased relative to in-person visits (all racial/ethnic minorities had increased telemedicine or video visit uptake), were mixed (some racial/ethnic minorities had increased telemedicine or video visit uptake while others had decreased uptake), or decreased (all racial/ethnic had decreased telemedicine defined as telephone and/or video visit uptake relative to in-person visits). Of these groups, uptake varied significantly. Among the 73 studies that included Black patients, 31 (42%) reported increased uptake, 27 (37%) reported decreased uptake, and 15 (21%) reported mixed uptake. Among the 66 studies that included Hispanic/Latinx patients, 28 (42%) reported increased uptake, 24 (36%) reported decreased uptake, and 14 (21%) reported mixed uptake. Among the 45 studies that included Asian patients, 15 (33%) reported increased uptake, 17 (38%) reported mixed uptake, and 13 (29%) reported decreased uptake. Among the 18 studies that included NA/AN patients, 4 (22.2%) reported increased uptake, 8 (44.4%) reported mixed uptake, and 6 (33.3%) reported decreased uptake. Among the 13 studies that included NH/PI patients, 4 (30.7%) reported increased uptake, 7 (53.8%) reported mixed uptake, and 2 (15.4%) reported decreased uptake. These results suggest that Black and Hispanic/Latinx populations were more likely to experience increased uptake, although a substantial number of studies still reported decreased or mixed patterns. In contrast, Asian, NA/AN, and NH/PI populations more frequently experienced decreased or mixed uptake. A visual representation of these reported uptakes is available in Fig 3.

### Comparison of telemedicine modalities utilized by racial/ethnic minorities

Across studies that evaluated differences in modality, we observed variation in the uptake of video, telephone, and in-person care among racial and ethnic minority groups. Of studies that included direct comparisons between video-only and in-person visits (n = 5), a majority (n = 4) reported decreased telemedicine uptake among racial/ethnic minority groups. Of the studies that evaluated all three modalities (video, telephone, and in-person visits) (n = 23), a minority reported increased video visit uptake (n = 7), while fewer studies reported increased telephone visit uptake (n = 6). A larger share (n = 9) of studies reported decreased uptakes of video and telephone visits relative to in-person visits, while other studies reported mixed uptake (n = 6) across all modalities. Among studies that compared telemedicine to in-person visits (n = 31), a larger proportion of studies reported decreased uptake or increased in-person visit uptake among racial/ethnic minorities (n = 15). A smaller proportion reported increased (n = 9) and mixed uptake (n = 7). A small minority of studies examined video-only visits versus telephone visits (n = 5), with mixed results across racial/ethnic groups. When broken down by racial/ethnic minority group, Black and Hispanic/Latinx patients were the most frequently represented in studies showing increased telephone but decreased video visit uptake, suggesting a modality gap. Asian, NA/AN, and NH/PI groups were more often included in studies reporting decreased or mixed uptake across both telephone and video modalities. A subset of studies (fewer than 10%) described a reversion to in-person care for racial/ethnic minority groups post-lockdown, but few provided systematic or longitudinal data to quantify these shifts.

### Sub-analysis of telemedicine utilization across distinct periods

Multi-period analyses demonstrated variable trends in telemedicine utilization over time, including decreased video use post-lockdown in some minority populations. A total of 34 studies examined telemedicine utilization across distinct study periods. Of these,11 were cohort studies that utilized the pre-pandemic period as the baseline period. Among studies comparing service utilization in pre- versus during-pandemic periods among racial/ethnic groups, 7 reported greater utilization of in-person visits during the pre-pandemic periods, 4 reported greater telemedicine utilization during the pandemic periods, and 4 reported no differences in utilization. Four studies observed mixed utilization patterns across race/ethnicity groups during the pandemic, and 2 studies observed no differences in utilization during intra-pandemic periods. Two studies did not adequately disaggregate telemedicine utilization data to ascertain differential use patterns across examined racial/ethnic groups and study periods.

### Associated factors of telemedicine utilization

**Fig 4:**
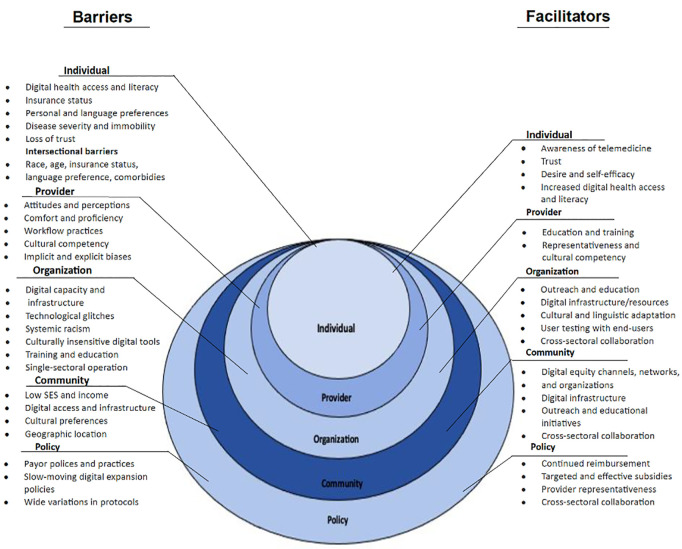
Model of multilevel factors associated with telemedicine utilization among racial/ethnic minority populations during the pandemic.

Of the 77 included studies focused on sociodemographic disparities in telemedicine utilization, we qualitatively evaluated a representative sample of 20 studies to identify associated factors, particularly barriers and facilitators, of telemedicine utilization. Our thematic evaluation was organized across multiple socio-ecological levels: individual, provider, organization, community, and policy (Fig 4).

#### Individual level.

At the individual level, digital access barriers and literacy challenges were the most commonly reported impediments to telemedicine utilization among racial/ethnic minorities. Barriers at the individual level centered on access to and the ability to use digital tools and technologies. (Fig 4) Identified barriers included access to quality internet and broadband connectivity [15,57,58,64] and digital technologies such as smartphones, computers, laptops, and tablets [38,58,61]. Other barriers included literacy skills, including health and digital literacy [15,34,38,62]. Digital inequity-related barriers were widely identified as prohibitory to virtual care utilization among racial/ethnic minorities during the pandemic. Other barriers included mistrust in providers [15,35,58,61], insurance status, personal and language preferences, disease severity, and immobility. The following sociodemographic barriers cut across examined studies and emerged at the intersection of race and ethnicity, older age, non-English language preference, public insurance, and disease comorbidity [35,38,58,64,67]. Notable facilitators included improved digital access and awareness of telemedicine [58,59], increased trust and confidence in providers [15,34,35,58,65,66], health systems, and telemedicine, enhanced literacy skills [57,58,62], improved self-efficacy [15,58,63], and a desire to use telemedicine [14,63].

#### Provider level.

Overall, provider training, comfort with telemedicine technologies, and biases were key factors associated with the utilization of telemedicine among minorities. Emergent barriers included providers’ attitudes and perceptions of telemedicine [15,34,38]. Providers’ attitude towards telemedicine was frequently associated with telemedicine reimbursement policies. Additional barriers included providers’ comfort levels and proficiency with telemedicine and poorly streamlined scheduling and workflow processes, which impeded care delivery services and reduced telemedicine visit volumes [35,39,59]. Cultural competency and implicit and explicit biases were emphasized as barriers to improving minority telemedicine adoption rates [15,34,58,60,65]. It is well established that these barriers reinforce health inequities by reducing treatment adherence and eroding trust between patients and providers, and the broader healthcare delivery system [104–106]. Facilitators of telemedicine utilization included implicit bias and cultural competency education and training, essential to enhancing patient-provider relationships and improving clinical outcomes [15,59,65].

#### Organization level.

Organizational factors influencing telemedicine use included outreach strategies, infrastructure capabilities, and cultural tailoring of digital services. Emergent organizational barriers comprised limited targeted outreach to local communities [15,28,65], inadequate digital infrastructure [14,63,65], system-wide glitches [15,36,39], systemic racism [15,35,58], lack of culturally and linguistically inclusive technologies [57,63], and poor telemedicine training [14,59]. Strengthening partnerships with trusted community organizations such as churches, grocery stores, and barber shops was emphasized as a tangible solution to bridging digital equity gaps [58]. Other facilitators reflected building collaborative relationships with local community leaders [15,34,63,65] and implementing initiatives to increase telemedicine awareness and digital literacy [34,58]. Infrastructure improvements, such as platforms that support audio and video at varying internet speeds, were highlighted for reducing access and use barriers among individuals with low-to-moderate internet speeds [15,39,58]. Several studies reinforced the importance of culturally congruent digital tools, particularly for populations facing language or cultural barriers [15,34,65]. Additional facilitators reflected addressing system-wide errors by creating telemedicine support teams [15,39] and providing educational materials across multiple secure communication channels and language options [37,39,65]. Training programs to increase acceptance among rural and digitally disadvantaged populations were noted as important considerations [15,60,61]. Finally, studies emphasized the value of participatory design approaches for digital tools [15,65] and cross-sector collaborations across health, policy, and community organizations to promote digital equity [34,35,65].

#### Community level.

Low socioeconomic status (SES) and low income [15,57,58,61] were consistently reported community-level barriers that are frequently associated with low telemedicine adoption [15,58]. Other barriers reflected low community-level digital access, inadequate infrastructure, cultural preferences [15,36,66], and geographic location [61,64,65]. A recommended community-level facilitator was aligning community-based digital health equity initiatives with digital expansion efforts at affiliated healthcare organizations [58,63]. Community-based digital equity anchors, such as libraries and other public facilities that provide free broadband and internet access, were highlighted for their potential to increase telemedicine adoption rates among minority populations [67]. Furthermore, outreach and education across communities were emphasized for their potential to alleviate concerns about telemedicine use regarding patient data privacy, safety, and confidentiality [15,63,65]. Finally, policy solutions focused on increasing digital access at the community level were recommended facilitators for ameliorating digital equity barriers [65].

#### Policy level.

A widely reported barrier at the policy level was the lack of policies related to telemedicine reimbursement. Limited support for comprehensive telemedicine reimbursement substantially impacted telemedicine availability, particularly during the early periods of the pandemic [34,35,67]. Other policy-level barriers included glacially slow digital expansion policies and policies that led to varied telemedicine utilization practices across state lines [34,64,65,67]. Policy-level facilitators included policies that prioritized adequate and sustained reimbursements for both telephone and video visits [35,67]. Two studies emphasized the centrality of continuing reimbursements for telephone visits, given their effectiveness at improving access to care for racial/ethnic minorities during the public health emergency, compared to video visits [35,67]. Another emerging facilitator was increased subsidies for internet and data charges, which may facilitate increased broadband access and device ownership [34,58]. Additional facilitators included policies explicitly focused on improving provider representativeness in healthcare, which may expand access to care for medically underserved populations [58,65], and policies that promote intersectoral collaborations, particularly at the community and organizational levels [34,65].

## Discussion

In this study, we comprehensively analyzed COVID-19-era literature to examine telemedicine utilization patterns among racial/ethnic minorities. Persistent disparities in telemedicine adoption were observed across all modality comparisons—video-only versus in-person, video versus telephone versus in-person, and combined video/phone versus in-person visits. Telemedicine uptake varied by racial/ethnic group: increased use was more frequently reported among Black and Hispanic/Latino populations, while lower or mixed uptake was more commonly reported among Asian, Native American/Alaska Native (NA/AN), and Native Hawaiian/Pacific Islander (NH/PI) populations. Decreased use of video-based services compared to in-person visits was a consistent finding across studies. Most research focused on metropolitan populations, with fewer studies representing rural or Southern regions. Barriers to telemedicine use were identified at multiple levels, including limited digital access and literacy at the individual level, provider-related factors such as comfort and bias, organizational infrastructure gaps, community-level socioeconomic barriers, and policy challenges related to reimbursement and broadband access. Facilitators included improved digital infrastructure, culturally adapted services, community partnerships, and policies supporting sustained telemedicine access.

This review contributes to an emerging body of research mapping disparities in digital health. Our findings align with prior studies documenting limited tailoring of telemedicine services for Asians, NA/AN, and NH/PI groups, who often face cultural, language, geographic, and systemic barriers [107–111]. These groups remain understudied and underserved, further exacerbating digital inequities [112,113]. Conversely, observed gains in telemedicine utilization among Black and Hispanic/Latinx populations may reflect targeted equity initiatives, such as Spanish-language services, improved user interfaces, and community outreach that emerged during the pandemic [114–122]. While much of the literature focuses on primary and specialty care, the findings may not generalize to broader populations due to baseline disparities in healthcare access. Most studies were conducted in coastal metropolitan areas with relatively higher digital connectivity, while less research was published in underserved rural regions, particularly in the South and Midwest, where gaps in digital infrastructure, such as fiber optics, mobile networks, and digital devices, are more pronounced [123–125]. Many rural minority communities face compounded access barriers to care, including limited broadband, lower educational attainment, lower socioeconomic status, and transportation challenges, compared to their urban-dwelling counterparts. To increase digital access and promote economic growth in rural areas, policy efforts should consider strategic collaborations with local digital equity anchors, such as libraries, schools, and community centers [126]. Public-private partnerships with centrally located commercial structures, including malls, restaurants, dry cleaners, salons, gift shops, doctor’s offices, and interstate and intercounty public transportation systems, may play a pivotal role in widening access to high-speed internet in rural settings [127–129]. Strategically placed kiosks in high foot-traffic areas may also serve as a digital access lifeline in rural communities [130]. Additionally, digital expansion policies should incentivize increased concentrations of internet service markets in rural environments as infrastructure develops to increase capacity for digital adoption and stimulate wider economic development. Future research should aim to cultivate a broader understanding of the evolving patterns of digital health access and use across various geographic categories and clinical specialties and assess whether post-pandemic gains have been sustained.

Our analysis of factors associated with telemedicine utilization among racial/ethnic minority patients revealed a confluence of factors influencing telemedicine adoption across multiple levels of socio-ecological influence. Widely reported barriers to telemedicine utilization reflected systemic racism and trust in providers. While evidence on the impact of racism in virtual interactions is limited, the enduring legacy of systemic racism, having persisted throughout the decades, is shown to reduce minority individuals’ willingness to engage with the healthcare delivery system. A California-based study of 2,552 participants, the state with the largest publication representation in our sample, showed that 85.9% of non-Hispanic Black individuals who reported perceived race-based discrimination during the pandemic delayed/forwent receiving care relative to 63.3% who did not experience discrimination [131]. Other studies suggest that trust in providers was a fundamental driver of telemedicine use disparities [17,132]. In one New York City study with >52,000 patients, non-Hispanic Black individuals were four times more likely to use the emergency department during the pandemic than non-Hispanic White individuals [132]. Findings were believed to be associated with systemically induced barriers to accessing primary care services and a lack of trust in the effectiveness of telemedicine systems [17,132–134]. It is probable that minority patients who struggled with telemedicine utilization barriers, including limited technology proficiency and reticence to use telemedicine due to privacy, safety, and confidentiality reasons, experienced a compounded layer of utilization barriers due to racism, mistrust, or distrust in the healthcare delivery system during the pandemic [122,135]. Healthcare systems and organizations committed to addressing these barriers should consider enacting various community-based telemedicine initiatives with the dual aim of establishing goodwill, and fostering a sense of trust and connectedness while also providing proactive solutions to digital inequities, including improving telemedicine awareness, assessing end-user needs through participatory design processes for telemedicine and digital tool development, and providing digital literacy training. These efforts should be conducted in parallel with rigorous and extensive anti-bias training, thereby strengthening community-institution partnerships and improving patient engagement.

### Limitations

This review has several limitations. First, there was little consensus in how studies defined “telehealth” and “utilization,” thereby complicating comparisons and potentially inflating utilization estimates. To address this, we broadened our search terms. Additionally, the inconsistent or interchangeable use of the terms *telehealth* and *telemedicine* across studies posed a challenge for synthesis. Some studies used the terms synonymously, while others applied them to different modalities (e.g., video only versus video and telephone combined). This definitional ambiguity could have contributed to heterogeneity in findings and limited the clarity of modality-specific uptake trends. Future work would benefit from standardized definitions to facilitate more precise comparisons across studies. Second, many included studies were cross-sectional, and a large fraction had short study duration periods. These limitations restricted our ability to infer causality and assess longitudinal patterns of adoption among racial/ethnic minorities. Though high satisfaction with telemedicine was reported during the pandemic [136], recent data suggest a resurgence of in-person care, further highlighting the need for future research on post-pandemic acceptance of technology [2]. Third, our review focused on synchronous telemedicine modalities, excluding asynchronous communication methods (e.g., email, secure messaging, patient portal). While synchronous formats are central to routine care delivery, excluding asynchronous modalities limits our capacity to more thoroughly assess the extent of telemedicine use disparities across all telemedicine formats. Fourth, a single coder performed the qualitative analysis; although thematic content was clear and consistent, subjective interpretation may have introduced bias. A single coder also extracted some data elements outside our Covidence workflow, which could have also introduced bias. Fifth, the exclusion of RCTs may have negatively impacted our study findings since we did not include rigorous comparisons of telemedicine adoption interventions. This imposed a challenge to establishing a causal link between telemedicine utilization patterns and sociodemographic disposition. Sixth, the review was restricted to U.S.-based studies. While findings from other geographical contexts may provide novel insights into minority telemedicine use patterns and associated factors, the unique complexities of the American healthcare delivery system, including its provider payment systems, extensive heterogeneity of the general U.S. population, and prevailing structural inequities, warrant focused examination. Seventh, only English-language, peer-reviewed studies were included, which may have limited the generalizability of our study. Finally, following PRISMA-ScR guidelines, we did not formally appraise study quality, which was consistent with scoping review conventions. However, studies examined in this review may be subject to several methodological limitations, including missing data from informal and unstructured EHR data collection (i.e., text messages, unstructured documentation, or undocumented phone calls), leading to underreported utilization estimates. Populations considered digitally disenfranchised, such as racial/ethnic minorities and geographically rural populations, may be significantly underrepresented in analyses due to digital access barriers, including limited digital literacy, inadequate internet access, and lack of device ownership. This has important implications for the internal and external validity of the studies under review. Cohort studies may be limited by the inability to assess the temporal influences that underlie technology utilization patterns, such as policy, COVID-19 variants, or rapid technology diffusion in response to pandemic-induced restrictions.

## Conclusion

There was a significant upsurge in studies exploring telemedicine utilization during the COVID-19 pandemic. Findings from this work demonstrated that patterns of telemedicine use were heterogeneous across racial/ethnic minority populations in reported studies, suggesting that differences in uptake were likely influenced by disparities-related conditions, including socioeconomic status (SES) and technological barriers, as well as related factors such as availability and provider capability. Efforts to address inequitable access to telemedicine for race/ethnic minorities and other vulnerable populations will require a robust understanding of a constellation of factors that are involved in telemedicine use, including addressing barriers at every socio-ecological level of care.

## Supporting information

S1 File Full supplemental table. (DOCX)

S2 File Master coding index. (DOCX)

S3 File PRISMA checklist. (DOCX)

S4 File Search strategies. (DOCX)

S5 File Search terms. (XLSX)

S6 File Data spreadsheet. (XLSX)

S7 File Qualitative data. (DOCX)

S8 File Coding document. (DOCX)
