# Supplementary material for: Video-based telemedicine utilization patterns and associated factors among racial and ethnic minorities in the United States during the COVID-19 pandemic: A mixed-methods scoping review
Source: PLOS Digit Health. 2025 Jul 24;4(7):e0000952. doi: 10.1371/journal.pdig.0000952 (PMC12289041; doi:10.1371/journal.pdig.0000952)
Supplement: S1 File — (DOCX) [file pdig.0000952.s001.docx]

| **Author (year)** | **Study design** | **Unit**  **of analysis** | **Sample size** | **Geo-graphic region** | **State** | **Clinical specialty** | **Race/**  **ethnicity groups included** | **Modalities compared** | **Telemedicine Utilization** |
| --- | --- | --- | --- | --- | --- | --- | --- | --- | --- |
| Almandoz et al., 2023  [[1]](https://sciwheel.com/work/citation?ids=13441487&pre=&suf=&sa=0) | Cross-sectional | Patient visits | 583 | South | Texas | Obstetrics | NHB, H/L | Video and telephone and In-person | More use by NHB |
| Anderson et al., 2023  [[2]](https://sciwheel.com/work/citation?ids=18004609&pre=&suf=&sa=0) | Cohort | Patient visits | 212 | Northeast | Connecticut | Pediatric neurosurgery | NHB, H/L, AS | Video and telephone and In-person | Less use by H/L |
| Annapragada et. al., 2021 [[3]](https://sciwheel.com/work/citation?ids=14654934&pre=&suf=&sa=0) | Cohort | Patient visit | 13,979 | Northeast | Maryland | Surgery | NHB | Video and telephone and In-person | No difference |
| Brown et al., 2021  [[4]](https://sciwheel.com/work/citation?ids=11993540&pre=&suf=&sa=0) | Cross-sectional | Patient visits | 73,465 | South | Multi-state | Pediatrics | NHB, H/L | Video, In-person, and Telephone | Less use by NHB and H/L |
| Bustamante et al., 2023  [[5]](https://sciwheel.com/work/citation?ids=15358607&pre=&suf=&sa=0) | Cohort | Encounter visits | 118,963 | West | California | Multiple specialties | NHB, H/L, AS | Video, In-person, and Telephone | Less use by NHB and H/L |
| Campos-Castillo et al., 2021 [[6]](https://sciwheel.com/work/citation?ids=9607029&pre=&suf=&sa=0) | Cross-sectional | Patient visits | 10,624 | National | National | Multiple specialties | NHB, H/L | Video, In-person, and Telephone | More use by NHB and less use by H/L |
| Campos-Castillo., 2022  [[7]](https://sciwheel.com/work/citation?ids=13943861&pre=&suf=&sa=0) | Cross-sectional | Patient visits | 532 | National | National | Behavioral health | NHB, H/L | Video, In-person, and Telephone | Less use by NHB and more use by H/L |
| Chen et al., 2022  [[8]](https://sciwheel.com/work/citation?ids=11407878&pre=&suf=&sa=0) | Cross-sectional | Patient visits | 5,023 | Northeast | Connecticut | Ophthalmology | NHB, H/L | Video, In-person, and Telephone | Less use by NHB and H/L |
| Chen et al., 2023  [[9]](https://sciwheel.com/work/citation?ids=15449872&pre=&suf=&sa=0) | Cohort | Patient visits | 247,287 | Northeast | New York | Primary care | NHB, H/L, AS | Video, In-person, and Telephone | Less use across all race/ethnicity groups |
| Childs et al., 2021  [[10]](https://sciwheel.com/work/citation?ids=11289218&pre=&suf=&sa=0) | Cohort | Patient visits | 1,008 | Northeast | Connecticut | Psychiatry | NHB, H/L | Video, In-person, and Telephone | Less use by H/L and no change for NHB |
| Chumbler et al., 2023  [[11]](https://sciwheel.com/work/citation?ids=15413788&pre=&suf=&sa=0) | Cohort | Patient visits | 13, 962 | South | Tennessee | Ambulatory care | NHB, H/L | Video and Telephone | More use by H/L |
| Chunara et al., 2021  [[12]](https://sciwheel.com/work/citation?ids=9578396&pre=&suf=&sa=0) | Cohort | Patient visits | 494,322 | Northeast | New York | Multiple specialties | NHB, H/L, AS | Video and In-person | Less use by NHB |
| Cordasco et al., 2022  [[13]](https://sciwheel.com/work/citation?ids=13844624&pre=&suf=&sa=0) | Cross-sectional | Patient visits | 13,469 | Multi-regional | Multi-state | Urgent care | NHB, H/L | Video, In-person, and Telephone | More use by NHB |
| Cousins et al., 2022  [[14]](https://sciwheel.com/work/citation?ids=15449884&pre=&suf=&sa=0) | Cohort | Patient visits | 62,172 | Midwest | Michigan | Oncology | NHB, H/L, AS, NA/AN | Video, In-person and Telephone | Mixed use depending on subspecialities |
| D'Amico et al., 2023  [[15]](https://sciwheel.com/work/citation?ids=16320850&pre=&suf=&sa=0) | Cohort | Encounter visits | 129,816 | Midwest | Ohio | Primary care | NHB, AS, NA/AN, NH/PI | Video, In-person, and Telephone | No difference for NHB and less video visit use across all other groups |
| Darrat et al., 2021 [[16]](https://sciwheel.com/work/citation?ids=10386620&pre=&suf=&sa=0) | Cohort | Patient visits | 1,162 | Midwest | Michigan | Otolaryngology | NHB | Video, In-person, and Telephone | Less use by NHB |
| Der-Martirosian et al., 2022 [[17]](https://sciwheel.com/work/citation?ids=13602672&pre=&suf=&sa=0) | Cohort | Patient visits | 113,091 | West | California | Multiple specialties | NHB, H/L | Video, In-person, Telephone, Video and telephone | More use by NBH and less use by H/L |
| Drake et al., 2022  [[18]](https://sciwheel.com/work/citation?ids=10931819&pre=&suf=&sa=0) | Cohort | Encounter visits | 624, 886 | South | North Carolina | Multiple specialties | NHB, AS NA/AN, NH/PI | Video, In-person, and Telephone | Less use by NHB, H/L and AS |
| Duan et al., 2022  [[19]](https://sciwheel.com/work/citation?ids=18004578&pre=&suf=&sa=0) | Cross-sectional | Encounter visits | 1,444 | Midwest | Illinois | Specialty care | NHB, H/L, AS | Video and In-person | Less use by NHB and no difference for H/L |
| Eberly et. al., 20222  [[20]](https://sciwheel.com/work/citation?ids=10228368&pre=&suf=&sa=0) | Cohort | Patient visits | 148,402 | Northeast | Multiple states | Multiple specialties | NHB, H/L, AS | Video, in-person, and telephone | Less use by AS and more use by NHB and H/L |
| Ekwegh et al., 2023  [[21]](https://sciwheel.com/work/citation?ids=15255262&pre=&suf=&sa=0) | Cross-sectional | Patient visits | 150 | West | California | Multiple specialties | NHB | Video, In-person, and Telephone | No difference |
| Ennis et al., 2022  [[22]](https://sciwheel.com/work/citation?ids=11704193&pre=&suf=&sa=0) | Cross-sectional | Patient visits | 4,873 | South | Multiple states | Primary care | NHB, H/L | Video and Telephone | Less use by NHB and H/L |
| Eruchalu et al., 2022  [[23]](https://sciwheel.com/work/citation?ids=12679868&pre=&suf=&sa=0) | Cohort | Encounter visits | 5,908 | Northeast | Massachusetts | Surgery | NHB, H/L | Video, In-person, and Telephone | Less use by NHB |
| Esper et al., 2021  [[24]](https://sciwheel.com/work/citation?ids=16537464&pre=&suf=&sa=0) | Cross-sectional | Encounter visits | 686 | South | Georgia | Neurology | NHB; AS | Video, In-person, and Telephone | Less use by NHB and AS |
| Flowers et al., 2023  [[25]](https://sciwheel.com/work/citation?ids=17907904&pre=&suf=&sa=0) | Cohort | Patient visits | 13,608 | Northeast | Rhode Island | Behavioral health | NHB; H/L | Video, In-person, Video and telephone | More use by H/L and less use by NHB |
| Freed et al., 2023  [[26]](https://sciwheel.com/work/citation?ids=18004638&pre=&suf=&sa=0) | Cohort | Encounter visits | 63,665 | South | North Carolina | Multiple specialties | NHB, H/L | Video, In-person, and Telephone | Less use by NHB and H/L |
| French et al., 2023  [[27]](https://sciwheel.com/work/citation?ids=15449880&pre=&suf=&sa=0) | Cohort | Patient visits | 54,996 | South | North Carolina | Behavioral health | NHB, H/L | Video, In-person, Telephone, Video and telephone | Less use by NHB and H/L |
| Friedman et al., 2022  [[28]](https://sciwheel.com/work/citation?ids=12682590&pre=&suf=&sa=0) | Cross-sectional | Patient visits | 856 | Midwest | Illinois | Infectious diseases | NHB | Video, In-person, and Telephone | Less use by NHB |
| Gao et al., 2022  [[29]](https://sciwheel.com/work/citation?ids=15512658&pre=&suf=&sa=0) | Cross-sectional | Patient visits | 2,521 | South | Tennessee | Obstetrics | NHB, H/L, AS | Video, In-person, and Video and telephone | Less use by NHB; H/L |
| Govier et al., 2022  [[30]](https://sciwheel.com/work/citation?ids=15540091&pre=&suf=&sa=0) | Cohort | Patient visits | 11,326 | Multi-regional | Multi-State | Multiple specialties | NHB, H/L, AS, NA/AN, NH/PI | Video, In-person, and Telephone | More use by NHB and less use by H/L |
| Grefe et al., 2023  [[31]](https://sciwheel.com/work/citation?ids=18004541&pre=&suf=&sa=0) | Cohort | Patient visits | 1,250 | South | Multi-state | Neurology | NHB, H/L | Video, In-person, and Telephone | Less use by NHB and H/L |
| Haynes et al., 2021  [[32]](https://sciwheel.com/work/citation?ids=11113852&pre=&suf=&sa=0) | Cohort | Patient visits | 1,292 | West | California | Endocrinology | NHB, H/L, AS, NA/AN, NH/PI | Video, In-person, and Telephone | No difference |
| He et al., 2023 [[33]](https://sciwheel.com/work/citation?ids=15824042&pre=&suf=&sa=0) | Cross-sectional | Patient visits | 3,621 | National | National | Multiple specialties | NHB, H/L | Video, In-person, and Telephone | Less video use by NHB and H/L |
| Hill et al., 2021  [[34]](https://sciwheel.com/work/citation?ids=11895387&pre=&suf=&sa=0) | Cross-sectional | Encounter visits | 3,142 | South | Multi-state | Reproductive health | NHB, H/L | Video, In-person, and Telephone | Less use by NHB and no difference for H/L |
| Huang et al., 2022  [[35]](https://sciwheel.com/work/citation?ids=18004536&pre=&suf=&sa=0) | Cohort | Patient visits | 64,011 | West | California | Multiple specialties | NHB, H/L, AS | Video, In-person, and Video and telephone | No differences across hospitalized groups and more use among non-hospitalized AS |
| Jallow et al., 2022  [[36]](https://sciwheel.com/work/citation?ids=18004581&pre=&suf=&sa=0) | Cohort | Encounter visits | 12,620 | Northeast | District of Columbia | Dermatology | NHB, H/L, AS | Video, In-person, and Telephone | More use by AS and less use by NHB and H/L |
| Khatana et al., 2022  [[37]](https://sciwheel.com/work/citation?ids=15812176&pre=&suf=&sa=0) | Cross-sectional | Patient visits | 1,999,534 | National | National | Multiple specialties | NHB, H/L | Video, In-person, and Video and telephone | Less use by NHB and H/L |
| Kolb et al., 2021  [[38]](https://sciwheel.com/work/citation?ids=14601750&pre=&suf=&sa=0) | Cross-sectional | Encounter visits | 7,392 | Northeast | Delaware | Otolaryngology | NHB, AS, NA/AN, NH/PI | Video, In-person, and Telephone | Less use across all race/ethnicity groups |
| Kummer et al., 2022  [[39]](https://sciwheel.com/work/citation?ids=18004544&pre=&suf=&sa=0) | Cohort | Patient visits | 14,170 | Northeast | New York | Neurology | NHB, AS, NA/AN, NH/PI | Video, In-person, and Telephone | Less use across all race/ethnicity groups |
| Kusters et al., 2023  [[40]](https://sciwheel.com/work/citation?ids=18004650&pre=&suf=&sa=0) | Cohort | Patient visits | 1, 754 311 | National | National | Behavioral Health | NA/AN | Video, In-person, and Telephone | Less use by rural NA/AN |
| Lamb et al., 2022  [[41]](https://sciwheel.com/work/citation?ids=18004652&pre=&suf=&sa=0) | Cross-sectional | Patient visits | 5,717 | Northeast | Pennsylvania | Dermatology | NHB, H/L, AS | Video and Telephone | More video visit use across all groups |
| Lambert et al., 2021  [[42]](https://sciwheel.com/work/citation?ids=13320017&pre=&suf=&sa=0) | Cross-sectional | Patient visits | 682 | Northeast | Connecticut | Pediatric neurosurgery | NHB, H/L | Video, In-person, and Telephone | Less use by NHB and H/L |
| Lee et al., 2023  [[43]](https://sciwheel.com/work/citation?ids=15539454&pre=&suf=&sa=0) | Cohort | Patient visits | 1,067,798 | National | National | Multiple specialties | NHB, H/L, AS | Video, In-person, Telephone, and Video and telephone | More telephone use by NHB |
| Lin et al., 2023 [[44]](https://sciwheel.com/work/citation?ids=18004542&pre=&suf=&sa=0) | Cross-sectional | Patient visits | 6,967 | National | National | Behavioral health | NHB, H/L, AS | Video, In-person, and Video and telephone | No differences |
| Lott et al., 2021  [[45]](https://sciwheel.com/work/citation?ids=16537534&pre=&suf=&sa=0) | Cohort | Encounter visits | 5,035 | Northeast | New York | Orthopedics | NHB, AS, NA/AN, NH/PI | Video and In-person | Less use by NHB, H/L and AS |
| Mahmud et al., 2020  [[46]](https://sciwheel.com/work/citation?ids=18004655&pre=&suf=&sa=0) | Cohort | Encounter visits | 11,828 | Multi-regional | National | Hepatology | NHB, H/L, AS | Video, In-person, Video and telephone | No differences |
| Merz-Herrala et al., 2023 [[47]](https://sciwheel.com/work/citation?ids=18004657&pre=&suf=&sa=0) | Cohort | Patient visits | 2,031 | National | National | Reproductive health | NHB, H/L, AS, NA/AN | Video, In-person, and Video and telephone | Less use across all race/ethnicity groups |
| Mueller et al., 2022  [[48]](https://sciwheel.com/work/citation?ids=14054833&pre=&suf=&sa=0) | Cohort | Patient visits | 3,314 | Northeast | New York | Multiple specialties | NHB, H/L, AS | Video, In-person, and Telephone | Less use by NHB and H/L |
| Naqvi et al., 2023  [[49]](https://sciwheel.com/work/citation?ids=18004658&pre=&suf=&sa=0) | Cross-sectional | Encounter visits | 2,024 | Multi-regional | Multi-state | Multiple specialties | NHB, H/L, AS | Video, In-person, and Telephone | Less use by NHB and H/L |
| Neeman et al., 2022  [[50]](https://sciwheel.com/work/citation?ids=12682705&pre=&suf=&sa=0) | Cohort | Patient visits | 46,052 | West | California | Oncology | NHB, H/L, AS, NH/PI | Video, In-person, and Telephone | More telephone use across all race/ethnicity groups |
| Osmanlliu et al., 2023  [[51]](https://sciwheel.com/work/citation?ids=15380983&pre=&suf=&sa=0) | Cohort | Encounter visits | 113,888 | West | California | Cardiology | NHB, H/L, AS | Video, In-person, Telephone, and Video and telephone | Less use by NHB and H/L |
| Pagan et al., 2020  [[52]](https://sciwheel.com/work/citation?ids=12761788&pre=&suf=&sa=0) | Cohort | Patient visits | 19,376 | West | California | Primary care | NHB, AS, NA/AN; NH/PI | Video, In-person, and Telephone | Less video use by NHB, H/L and AS |
| Palzes et al., 2023  [[53]](https://sciwheel.com/work/citation?ids=15231006&pre=&suf=&sa=0) | Cohort | Patient visits | 36,607 | West | California | Multiple specialties | NHB, H/L, AS, NA/AN, NH/PI | Video, In-person, and Video and telephone | No differences |
| Pitaro et al., 2022  [[54]](https://sciwheel.com/work/citation?ids=18004663&pre=&suf=&sa=0) | Cohort | Patient visits | 3,090 | Northeast | New York | Multiple specialties | H/L | Video, In-person, and Video and telephone | Less use by H/L |
| Pritchett et al., 2022  [[55]](https://sciwheel.com/work/citation?ids=18004664&pre=&suf=&sa=0) | Cross-sectional | Patient visits | 101,756 | Multi-regional | Multi-state | Multiple specialties | H/L | Video, In-person, and Video and telephone | Less use by H/L |
| Rametta et al., 2020  [[56]](https://sciwheel.com/work/citation?ids=9090968&pre=&suf=&sa=0) | Cohort | Encounter visits | 17,369 | Northeast | Pennsylvania | Neurology | NHB, H/L, AS | Video and telephone and In-person | Less video visit use across all race/ethnicity groups |
| Rockholt et al., 2023  [[57]](https://sciwheel.com/work/citation?ids=18004667&pre=&suf=&sa=0) | Cohort | Patient visits | 12,615 | Northeast | New York | Pain medicine | NHB, H/L, AS | Video and telephone and In-person | Less use across all race/ethnicity groups |
| Rovner et al., 2021  [[58]](https://sciwheel.com/work/citation?ids=11113843&pre=&suf=&sa=0) | Cross-sectional | Patient visits | 157 | Northeast | Pennsylvania | Endocrinology | NHB | Video and In-person | Less use by NHB |
| Ryskina et al., 2021  [[59]](https://sciwheel.com/work/citation?ids=11407883&pre=&suf=&sa=0) | Cross-sectional | Patient visits | 17,103 | Northeast | Pennsylvania | Primary care | NHB, H/L, AS | Video and telephone, In-person, and Telephone | More use by NHB and less use by H/L |
| Schenker et al., 2022  [[60]](https://sciwheel.com/work/citation?ids=15449885&pre=&suf=&sa=0) | Cross-sectional | Patient visits | 11,961 | West | California | Pediatrics | NHB, H/L, AS, NA/AN | Video and In-person | No differences |
| Schifeling et al., 2020  [[61]](https://sciwheel.com/work/citation?ids=10318135&pre=&suf=&sa=0) | Cross-sectional | Encounter visits | 190 | West | Colorado | Primary care | NHB, H/L, AS | Video and Telephone | Less use across all race/ethnicity groups |
| Sen et al., 2022  [[62]](https://sciwheel.com/work/citation?ids=12862778&pre=&suf=&sa=0) | Cohort | Patient visits | 106,478 | South | Alabama | Pediatrics | NHB, H/L, NA/AN | Video, In-person, and Video and telephone | Less use by NHB and H/L and more use by NA/AN |
| Sen et al., 2023  [[63]](https://sciwheel.com/work/citation?ids=15449881&pre=&suf=&sa=0) | Cross-sectional | Patient visits | 637,792 | South | Alabama | Pediatrics | NHB, H/L, AS, NA/AN | Video, In-person, and Video and telephone | Less use across all race/ethnicity groups |
| Shao et al., 2022  [[64]](https://sciwheel.com/work/citation?ids=12706776&pre=&suf=&sa=0) | Cohort | Encounter visits | 50,519 | South | Alabama | Oncology | NHB, H/L, AS | Video, In-person, Telephone, and Video and telephone | Less use by NHB |
| Shehan et al., 2021  [[65]](https://sciwheel.com/work/citation?ids=12138184&pre=&suf=&sa=0) | Cohort | Encounter visits | 8,013 | Northeast | Massachusetts | Otolaryngology | NHB, H/L, AS, NA/AN | Video and telephone and In-person | More use by H/L and AS |
| Smith et al., 2020  [[66]](https://sciwheel.com/work/citation?ids=10826189&pre=&suf=&sa=0) | Cross-sectional | Encounter visits | 7,530 | Northeast | Massachusetts | Neurology | NHB, H/L, AS | Video, In-person, Telephone, and Video and telephone | Less use by NHB; H/L; AS |
| Steven et al., 2021  [[67]](https://sciwheel.com/work/citation?ids=14060796&pre=&suf=&sa=0) | Cohort | Encounter visits | 310,675 | Northeast | Massachusetts | Multiple specialties | NHB, H/L, AS | Video and telephone and In-person | More use across all race/ethnicity groups |
| Tobin et al., 2023  [[68]](https://sciwheel.com/work/citation?ids=15449877&pre=&suf=&sa=0) | Cohort | Encounter visits | 1,075 | Midwest | Michigan | Primary care | NHB | Video, In-person, and Telephone | Less video visit use by NHB |
| Uscher-Pines et al., 2022 [[69]](https://sciwheel.com/work/citation?ids=12104017&pre=&suf=&sa=0) | Cohort | Patient visits | 549,306 | West | California | Pediatric | NHB, H/L, AS | Video and telephone and In-person | Less use by NHB and H/L |
| Vaughan et al., 2023  [[70]](https://sciwheel.com/work/citation?ids=16086515&pre=&suf=&sa=0) | Cohort | Patient visits | 5,410 | South | Texas | Cardiology | NHB, H/L, AS, NA/AN, NH/PI | Video and telephone and In-person | No differences |
| Walters et al., 2021  [[71]](https://sciwheel.com/work/citation?ids=18004490&pre=&suf=&sa=0) | Cross-sectional | Encounter visits | 767 | Midwest | Ohio | Pediatrics | NHB, H/L | Video and In-person | Less use by NHB |
| Weber et al., 2020  [[72]](https://sciwheel.com/work/citation?ids=10218290&pre=&suf=&sa=0) | Cross-sectional | Patient visits | 52,585 | Northeast | New York | Multiple specialties | NHB, H/L, AS | Video and telephone and In-person | Less use by NHB and H/L |
| Weber et al., 2023  [[73]](https://sciwheel.com/work/citation?ids=18004692&pre=&suf=&sa=0) | Cohort | Patient visits | 121,072 | Northeast | New York | Multiple specialties | NHB, H/L, AS | Video, In-person, and Telephone | Less video visit use by NHB and H/L |
| Wegermann et al., 2022  [[74]](https://sciwheel.com/work/citation?ids=11452356&pre=&suf=&sa=0) | Cohort | Encounter visits | 7,099 | South | North Carolina | Hepatology | NHB, H/L | Video and Telephone | Less video visit use across all race/ethnicity groups |
| Wong et al., 2021  [[75]](https://sciwheel.com/work/citation?ids=14601760&pre=&suf=&sa=0) | Cross-sectional | Patient visits | 122 | West | Neurology | Neurology | AS, NA/AN, NH/PI | Video and telephone and In-person | Less use across all race/ethnicity groups |
| Wood et al., 2021  [[76]](https://sciwheel.com/work/citation?ids=15141359&pre=&suf=&sa=0) | Cohort | Patient visits | 2930 | West | Washington | Infectious diseases | NHB, AS, NH/PI | Video, In-person, Telephone, and Video and telephone | Less video visit use across all race/ethnicity groups |
| Ye et al., 2021 [[77]](https://sciwheel.com/work/citation?ids=10575804&pre=&suf=&sa=0) | Cross-sectional | Patient visits | 50,101 | Northeast | New York | Multiple specialties | NHB, H/L, AS, NA/AN, NH/PI | Video and Telephone | Less use by NHB and H/L |

**References**

[1. Almandoz JP, Xie L, Schellinger JN, Mathew MS, Edwards K, Ofori A, et al. Telehealth utilization among multi-ethnic patients with obesity during the COVID-19 pandemic. J Telemed Telecare. 2023;29:530–9.](https://sciwheel.com/work/bibliography/13441487)

[2. Anderson MG, Lambert W, Leclair N, Athar D, Martin JE, Bookland MJ, et al. Telemedicine utilization in an outpatient pediatric neurosurgical clinic: A prospective survey of patient and family preferences. World Neurosurg. 2023;176:e557–68.](https://sciwheel.com/work/bibliography/18004609)

[3. Annapragada AV, Meshram P, Jenkins SG, Jain A, Middleton KK, Thakkar SC, et al. Age and racial disparities in telemedicine utilization in an academic orthopedic surgery department. Telemed J E Health. 2022;28:970–5.](https://sciwheel.com/work/bibliography/14654934)

[4. Brown CL, Montez K, Amati JB, Simeonsson K, Townsend JD, Orr CJ, et al. Impact of COVID-19 on Pediatric Primary Care Visits at Four Academic Institutions in the Carolinas. Int J Environ Res Public Health. 2021;18.](https://sciwheel.com/work/bibliography/11993540)

[5. Bustamante AV, Martínez LE, Jalal S, Benitez Santos N, Félix Beltrán L, Rich J, et al. Racial and ethnic disparities in telehealth use before and after California’s stay-at-home order. Front Public Health. 2023;11:1222203.](https://sciwheel.com/work/bibliography/15358607)

[6. Campos-Castillo C, Anthony D. Racial and ethnic differences in self-reported telehealth use during the COVID-19 pandemic: a secondary analysis of a US survey of internet users from late March. J Am Med Inform Assoc. 2021;28:119–25.](https://sciwheel.com/work/bibliography/9607029)

[7. Campos-Castillo C, Laestadius LI. Mental Healthcare Utilization, Modalities, and Disruptions During Spring 2021 of the COVID-19 Pandemic Among U.S. Adolescents. J Adolesc Health. 2022;71:512–5.](https://sciwheel.com/work/bibliography/13943861)

[8. Chen EM, Andoh JE, Nwanyanwu K. Socioeconomic and Demographic Disparities in the Use of Telemedicine for Ophthalmic Care during the COVID-19 Pandemic. Ophthalmology. 2022;129:15–25.](https://sciwheel.com/work/bibliography/11407878)

[9. Chen K, Zhang C, Gurley A, Akkem S, Jackson H. Patient Characteristics Associated with Telehealth Scheduling and Completion in Primary Care at a Large, Urban Public Healthcare System. J Urban Health. 2023;100:468–77.](https://sciwheel.com/work/bibliography/15449872)

[10. Childs AW, Bacon SM, Klingensmith K, Li L, Unger A, Wing AM, et al. Showing Up Is Half the Battle: The Impact of Telehealth on Psychiatric Appointment Attendance for Hospital-Based Intensive Outpatient Services During COVID-19. Telemed J E Health. 2021;27:835–42.](https://sciwheel.com/work/bibliography/11289218)

[11. Chumbler NR, Chen M, Harrison A, Surbhi S. Racial and Socioeconomic Characteristics Associated with the use of Telehealth Services Among Adults With Ambulatory Sensitive Conditions. Health Serv Res Manag Epidemiol. 2023;10:23333928231154336.](https://sciwheel.com/work/bibliography/15413788)

[12. Chunara R, Zhao Y, Chen J, Lawrence K, Testa PA, Nov O, et al. Telemedicine and healthcare disparities: a cohort study in a large healthcare system in New York City during COVID-19. J Am Med Inform Assoc. 2021;28:33–41.](https://sciwheel.com/work/bibliography/9578396)

[13. Cordasco KM, Yuan AH, Rollman JE, Moreau JL, Edwards LK, Gable AR, et al. Veterans’ Use of Telehealth for Veterans Health Administration Community Care Urgent Care During the Early COVID-19 Pandemic. Med Care. 2022;60:860–7.](https://sciwheel.com/work/bibliography/13844624)

[14. Cousins MM, Van Til M, Steppe E, Ng S, Ellimoottil C, Sun Y, et al. Age, race, insurance type, and digital divide index are associated with video visit completion for patients seen for oncologic care in a large hospital system during the COVID-19 pandemic. PLoS ONE. 2022;17:e0277617.](https://sciwheel.com/work/bibliography/15449884)

[15. D’Amico R, Schnell PM, Foraker R, Olayiwola JN, Jonas DE, Brill SB. The Evolution of Primary Care Telehealth Disparities During COVID-19: Retrospective Cohort Study. J Med Internet Res. 2023;25:e43965.](https://sciwheel.com/work/bibliography/16320850)

[16. Darrat I, Tam S, Boulis M, Williams AM. Socioeconomic disparities in patient use of telehealth during the coronavirus disease 2019 surge. JAMA Otolaryngol Head Neck Surg. 2021;147:287–95.](https://sciwheel.com/work/bibliography/10386620)

[17. Der-Martirosian C, Chu K, Steers WN, Wyte-Lake T, Balut MD, Dobalian A, et al. Examining telehealth use among primary care patients, providers, and clinics during the COVID-19 pandemic. BMC Prim Care. 2022;23:155.](https://sciwheel.com/work/bibliography/13602672)

[18. Drake C, Lian T, Cameron B, Medynskaya K, Bosworth HB, Shah K. Understanding telemedicine’s “new normal”: variations in telemedicine use by specialty line and patient demographics. Telemed J E Health. 2022;28:51–9.](https://sciwheel.com/work/bibliography/10931819)

[19. Duan GY, Ruiz De Luzuriaga AM, Schroedl LM, Rosenblatt AE. Disparities in telemedicine use during the COVID-19 pandemic among pediatric dermatology patients. Pediatr Dermatol. 2022;39:520–7.](https://sciwheel.com/work/bibliography/18004578)

[20. Eberly LA, Kallan MJ, Julien HM, Haynes N, Khatana SAM, Nathan AS, et al. Patient Characteristics Associated With Telemedicine Access for Primary and Specialty Ambulatory Care During the COVID-19 Pandemic. JAMA Netw Open. 2020;3:e2031640.](https://sciwheel.com/work/bibliography/10228368)

[21. Ekwegh T, Cobb S, Adinkrah EK, Vargas R, Kibe LW, Sanchez H, et al. Factors Associated with Telehealth Utilization among Older African Americans in South Los Angeles during the COVID-19 Pandemic. Int J Environ Res Public Health. 2023;20.](https://sciwheel.com/work/bibliography/15255262)

[22. Ennis N, Armas L, Butame S, Joshi H. Factors Impacting Video Telehealth Appointment Completion During COVID-19 Pandemic Among People Living with HIV in a Community-Based Health System. AIDS Behav. 2022;26:407–14.](https://sciwheel.com/work/bibliography/11704193)

[23. Eruchalu CN, Bergmark RW, Smink DS, Tavakkoli A, Nguyen LL, Bates DW, et al. Demographic Disparity in Use of Telemedicine for Ambulatory General Surgical Consultation During the COVID-19 Pandemic: Analysis of the Initial Public Health Emergency and Second Phase Periods. J Am Coll Surg. 2022;234:191–202.](https://sciwheel.com/work/bibliography/12679868)

[24. Esper CD, Scorr L, Papazian S, Bartholomew D, Esper GJ, Factor SA. Telemedicine in an Academic Movement Disorders Center during COVID-19. J Mov Disord. 2021;14:119–25.](https://sciwheel.com/work/bibliography/16537464)

[25. Flowers D, Goodspeed E, Daly M. Telehealth as an Effective Care Delivery Method During the COVID-19 Pandemic for the Rhode Island Behavioral Health Population. Community Ment Health J. 2024;60:108–14.](https://sciwheel.com/work/bibliography/17907904)

[26. Freed SS, Jones KA, Whitaker RG, Norman K, Carvalho M, Giri A, et al. Evaluating Telehealth Uptake Among North Carolina Medicaid Beneficiaries With Musculoskeletal Conditions: Insights From the COVID-19 Pandemic. Med Care. 2023;61:750–9.](https://sciwheel.com/work/bibliography/18004638)

[27. French A, Jones KA, Bettger JP, Maslow GR, Cholera R, Giri A, et al. Telehealth Utilization Among Adult Medicaid Beneficiaries in North Carolina with Behavioral Health Conditions During the COVID-19 Pandemic. J Racial Ethn Health Disparities. 2024;11:2663–75.](https://sciwheel.com/work/bibliography/15449880)

[28. Friedman EE, Devlin SA, Gilson SF, Ridgway JP. Age and Racial Disparities in Telehealth Use Among People with HIV During the COVID-19 Pandemic. AIDS Behav. 2022;26:2686–91.](https://sciwheel.com/work/bibliography/12682590)

[29. Gao C, Osmundson S, Malin BA, Chen Y. Telehealth Use in the COVID-19 Pandemic: A Retrospective Study of Prenatal Care. Stud Health Technol Inform. 2022;290:503–7.](https://sciwheel.com/work/bibliography/15512658)

[30. Govier DJ, Cohen-Cline H, Marsi K, Roth SE. Differences in access to virtual and in-person primary care by race/ethnicity and community social vulnerability among adults diagnosed with COVID-19 in a large, multi-state health system. BMC Health Serv Res. 2022;22:511.](https://sciwheel.com/work/bibliography/15540091)

[31. Grefe A, Chen S-H, Ip EH, Kirkendall E, Nageswaran S. Audio or video? access to pediatric neurology outpatient services varies by the type of telehealth, especially for black children. J Child Neurol. 2023;38:263–9.](https://sciwheel.com/work/bibliography/18004541)

[32. Haynes SC, Kompala T, Neinstein A, Rosenthal J, Crossen S. Disparities in Telemedicine Use for Subspecialty Diabetes Care During COVID-19 Shelter-In-Place Orders. J Diabetes Sci Technol. 2021;15:986–92.](https://sciwheel.com/work/bibliography/11113852)

[33. He Q, Keith D, Eckhoff DO, Park C, Ng BP. Accessibility and Utilization of Telehealth Services During the COVID-19 Pandemic Among Medicare Beneficiaries by Diabetes Status. Res Gerontol Nurs. 2023;16:134–46.](https://sciwheel.com/work/bibliography/15824042)

[34. Hill BJ, Lock L, Anderson B. Racial and ethnic differences in family planning telehealth use during the onset of the COVID-19 response in Arkansas, Kansas, Missouri, and Oklahoma. Contraception. 2021;104:262–4.](https://sciwheel.com/work/bibliography/11895387)

[35. Huang BZ, Creekmur B, Yoo MS, Broder B, Subject C, Sharp AL. Healthcare Utilization Among Patients Diagnosed with COVID-19 in a Large Integrated Health System. J Gen Intern Med. 2022;37:830–7.](https://sciwheel.com/work/bibliography/18004536)

[36. Jallow M, Ewulu A, Ajilore P, Hussain AN, Geng X, Cardis MA. Analyzing disparities in access to teledermatology compared with dermatology clinic visits before, during, and after COVID-19 quarantine. Clin Dermatol. 2023;41:207–14.](https://sciwheel.com/work/bibliography/18004581)

[37. Khatana SAM, Yang L, Eberly LA, Julien HM, Adusumalli S, Groeneveld PW. Predictors of telemedicine use during the COVID-19 pandemic in the United States-an analysis of a national electronic medical record database. PLoS ONE. 2022;17:e0269535.](https://sciwheel.com/work/bibliography/15812176)

[38. Kolb CM, Born K, Banker K, Barth PC, Aaronson NL. Improving Attendance and Patient Experiences During the Expansion of a Telehealth-Based Pediatric Otolaryngology Practice. Otolaryngol Head Neck Surg. 2021;164:952–8.](https://sciwheel.com/work/bibliography/14601750)

[39. Kummer BR, Agarwal P, Sweetnam C, Robinson-Papp J, Blank LJ, Katz Sand I, et al. Trends in the Utilization of Teleneurology and Other Healthcare Resources Prior to and During the COVID-19 Pandemic in an Urban, Tertiary Health System. Front Neurol. 2022;13:834708.](https://sciwheel.com/work/bibliography/18004544)

[40. Kusters IS, Amspoker AB, Frosio K, Day SC, Day G, Ecker A, et al. Rural-Urban Disparities in Video Telehealth Use During Rapid Mental Health Care Virtualization Among American Indian/Alaska Native Veterans. JAMA Psychiatry. 2023;80:1055–60.](https://sciwheel.com/work/bibliography/18004650)

[41. Lamb JE, Fitzsimmons R, Sevagamoorthy A, Kovarik CL, Shin DB, Takeshita J. Patient Factors Associated With Teledermatology Visit Type and Submission of Photographs During the COVID-19 Pandemic: Cross-sectional Analysis. JMIR Dermatol. 2022;5:e38694.](https://sciwheel.com/work/bibliography/18004652)

[42. Lambert WA, Leclair NK, Knopf J, Mosha MH, Bookland MJ, Martin JE, et al. Predictors of Telemedicine Utilization in a Pediatric Neurosurgical Population During the COVID-19 Pandemic. World Neurosurg. 2021;153:e308–14.](https://sciwheel.com/work/bibliography/13320017)

[43. Lee H, Singh GK. The Impact of Telemedicine Parity Requirements on Telehealth Utilization in the United States During the COVID-19 Pandemic. J Public Health Manag Pract. 2023;29:E147–56.](https://sciwheel.com/work/bibliography/15539454)

[44. Lin C, Pham H, Hser Y-I. Mental Health Service Utilization and Disparities in the U.S: Observation of the First Year into the COVID Pandemic. Community Ment Health J. 2023;59:972–85.](https://sciwheel.com/work/bibliography/18004542)

[45. Lott A, Sacks H, Hutzler L, Campbell KA, Lajam CM. Telemedicine Utilization by Orthopedic Patients During COVID-19 Pandemic: Demographic and Socioeconomic Analysis. Telemed J E Health. 2021;27:1117–22.](https://sciwheel.com/work/bibliography/16537534)

[46. Mahmud N, Goldberg DS, Kaplan DE, Serper M. Major shifts in outpatient cirrhosis care delivery attributable to the COVID‐19 pandemic: A national cohort study. Hepatol Commun. 2020;](https://sciwheel.com/work/bibliography/18004655)

[47. Merz-Herrala AA, Kerns JL, Logan R, Gutierrez S, Marshall C, Diamond-Smith N. Contraceptive care in the United States during the COVID-19 pandemic: A social media survey of contraceptive access, telehealth use and telehealth quality. Contraception. 2023;123:110000.](https://sciwheel.com/work/bibliography/18004657)

[48. Mueller BR, Lawrence S, Benn E, Nirenberg S, Kummer B, Jette N, et al. Disparities in telehealth utilization in patients with pain during COVID-19. Pain Rep. 2022;7:e1001.](https://sciwheel.com/work/bibliography/14054833)

[49. Naqvi IA, Cohen AS, Kim Y, Harris J, Denny MC, Strobino K, et al. Inequities in Telemedicine Use Among Patients With Stroke and Cerebrovascular Diseases: A Tricenter Cross-sectional Study. Neurol Clin Pract. 2023;13:e200148.](https://sciwheel.com/work/bibliography/18004658)

[50. Neeman E, Lyon L, Sun H, Conell CA, Reed M, Kumar D, et al. The future of tele-oncology: trends and disparities in telehealth and secure message utilization in the COVID-19 era. JCO. 2021;39:1506–1506.](https://sciwheel.com/work/bibliography/12682705)

[51. Osmanlliu E, Kalwani NM, Parameswaran V, Qureshi L, Dash R, Scheinker D, et al. Sociodemographic disparities in the use of cardiovascular ambulatory care and telemedicine during the COVID-19 pandemic. Am Heart J. 2023;263:169–76.](https://sciwheel.com/work/bibliography/15380983)

[52. Pagán VM, McClung KS, Peden CJ. An Observational Study of Disparities in Telemedicine Utilization in Primary Care Patients Before and During the COVID-19 Pandemic. Telemed J E Health. 2022;28:1117–25.](https://sciwheel.com/work/bibliography/12761788)

[53. Palzes VA, Chi FW, Metz VE, Sterling S, Asyyed A, Ridout KK, et al. Overall and Telehealth Addiction Treatment Utilization by Age, Race, Ethnicity, and Socioeconomic Status in California After COVID-19 Policy Changes. JAMA Health Forum. 2023;4:e231018.](https://sciwheel.com/work/bibliography/15231006)

[54. Pitaro NL, Barbera JP, Ranson WA, Zubizarreta N, Poeran J, Chen DD, et al. Evaluating Resource Utilization for In-Person and Virtual Joint Classes in Total Joint Arthroplasty: An Analysis of Attendance Patterns at a Large Metropolitan Health System. J Arthroplasty. 2022;37:1708–14.](https://sciwheel.com/work/bibliography/18004663)

[55. Pritchett J, Borah BJ, Dholakia R, Moriarty JP, Ahn H, Huang M, et al. Patient- and provider-level factors associated with telehealth utilization across a multisite, multiregional cancer practice. JCO. 2022;40:1512–1512.](https://sciwheel.com/work/bibliography/18004664)

[56. Rametta SC, Fridinger SE, Gonzalez AK, Xian J, Galer PD, Kaufman M, et al. Analyzing 2,589 child neurology telehealth encounters necessitated by the COVID-19 pandemic. Neurology. 2020;95:e1257–66.](https://sciwheel.com/work/bibliography/9090968)

[57. Rockholt MM, Addae G, Chee A, Chin W, Cuff G, Wang J, et al. Implementing Telemedicine During the COVID-19 Pandemic: Disparities in Utilization in an Urban Pain Medicine Practice. J Pain Res. 2023;16:2763–75.](https://sciwheel.com/work/bibliography/18004667)

[58. Rovner BW, Casten RJ, Chang AM, Hollander JE, Rising K. Mistrust, Neighborhood Deprivation, and Telehealth Use in African Americans with Diabetes. Popul Health Manag. 2021;24:699–700.](https://sciwheel.com/work/bibliography/11113843)

[59. Ryskina KL, Shultz K, Zhou Y, Lautenbach G, Brown RT. Older adults’ access to primary care: Gender, racial, and ethnic disparities in telemedicine. J Am Geriatr Soc. 2021;69:2732–40.](https://sciwheel.com/work/bibliography/11407883)

[60. Schenker RB, Laguna MC, Odisho AY, Okumura MJ, Burnett H. Are We Reaching Everyone? A Cross-Sectional Study of Telehealth Inequity in the COVID-19 Pandemic in an Urban Academic Pediatric Primary Care Clinic. Clin Pediatr (Phila). 2022;61:26–33.](https://sciwheel.com/work/bibliography/15449885)

[61. Schifeling CH, Shanbhag P, Johnson A, Atwater RC, Koljack C, Parnes BL, et al. Disparities in Video and Telephone Visits Among Older Adults During the COVID-19 Pandemic: Cross-Sectional Analysis. JMIR Aging. 2020;3:e23176.](https://sciwheel.com/work/bibliography/10318135)

[62. Sen BP, Sharma P, Brisendine A, Blackburn J, Morrisey M, Menachemi N, et al. Disparities in Telehealth Utilization in a Population of Publicly Insured Children During the COVID-19 Pandemic. Popul Health Manag. 2022;25:178–85.](https://sciwheel.com/work/bibliography/12862778)

[63. Sen B, Rahim MJ, McDougal J, Sharma P, Yang N, Brisendine A, et al. Telehealth use among pediatric Alabama Medicaid enrollees, March-December 2020: Variations by race/ethnicity & place of residence. PLoS ONE. 2023;18:e0287598.](https://sciwheel.com/work/bibliography/15449881)

[64. Shao CC, McLeod MC, Gleason LT, Dos Santos Marques IC, Chu DI, Wallace EL, et al. Inequity in Telemedicine Use Among Patients with Cancer in the Deep South During the COVID-19 Pandemic. Oncologist. 2022;27:555–64.](https://sciwheel.com/work/bibliography/12706776)

[65. Shehan JN, Agarwal P, Danis DO, Ghulam-Smith M, Bloom J, Piraquive J, et al. Effects of COVID-19 on telemedicine practice patterns in outpatient otolaryngology. Am J Otolaryngol. 2021;42:103044.](https://sciwheel.com/work/bibliography/12138184)

[66. Smith CB, Bhardwaj AS. Disparities in the use of telehealth during the COVID-19 pandemic. JCO. 2020;38:87–87.](https://sciwheel.com/work/bibliography/10826189)

[67. Stevens JP, Mechanic O, Markson L, O’Donoghue A, Kimball AB. Telehealth Use by Age and Race at a Single Academic Medical Center During the COVID-19 Pandemic: Retrospective Cohort Study. J Med Internet Res. 2021;23:e23905.](https://sciwheel.com/work/bibliography/14060796)

[68. Tobin ET, Hadwiger A, DiChiara A, Entz A, Miller-Matero LR. Demographic Predictors of Telehealth Use for Integrated Psychological Services in Primary Care During the COVID-19 Pandemic. J Racial Ethn Health Disparities. 2023;10:1492–8.](https://sciwheel.com/work/bibliography/15449877)

[69. Predmore ZS, Roth E, Breslau J, Fischer SH, Uscher-Pines L. Assessment of Patient Preferences for Telehealth in Post-COVID-19 Pandemic Health Care. JAMA Netw Open. 2021;4:e2136405.](https://sciwheel.com/work/bibliography/12104017)

[70. Vaughan RM, Moore JA, Moreno JS, Dyer KJ, Oluyomi AO, Lopez KN. Remote Care Adoption in Underserved Congenital Heart Disease Patients During the COVID-19 Era. Pediatr Cardiol. 2023;44:404–12.](https://sciwheel.com/work/bibliography/16086515)

[71. Walters J, Johnson T, DeBlasio D, Klein M, Sikora K, Reilly K, et al. Integration and impact of telemedicine in underserved pediatric primary care. Clin Pediatr (Phila). 2021;60:452–8.](https://sciwheel.com/work/bibliography/18004490)

[72. Weber E, Miller SJ, Astha V, Janevic T, Benn E. Characteristics of telehealth users in NYC for COVID-related care during the coronavirus pandemic. J Am Med Inform Assoc. 2020;27:1949–54.](https://sciwheel.com/work/bibliography/10218290)

[73. Weber E, Miller SJ, Shroff N, Beyrouty M, Calman N. Recent telehealth utilization at a large federally qualified health center system: evidence of disparities even within telehealth modalities. Telemed J E Health. 2023;29:1601–12.](https://sciwheel.com/work/bibliography/18004692)

[74. Wegermann K, Wilder JM, Parish A, Niedzwiecki D, Gellad ZF, Muir AJ, et al. Racial and Socioeconomic Disparities in Utilization of Telehealth in Patients with Liver Disease During COVID-19. Dig Dis Sci. 2022;67:93–9.](https://sciwheel.com/work/bibliography/11452356)

[75. Wong VSS, Williams MK, Akiona CK, Avalos LN, Taylor EJ, Stein AG, et al. Demographic and technological factors influencing virtual seizure clinic visit satisfaction before and during the Covid-19 pandemic in rural Hawaii. Epilepsy Behav. 2021;124:108374.](https://sciwheel.com/work/bibliography/14601760)

[76. Wood BR, Lan KF, Tao Y, Mose EY, Aas E, Budak JZ, et al. Visit Trends and Factors Associated With Telemedicine Uptake Among Persons With HIV During the COVID-19 Pandemic. Open Forum Infect Dis. 2021;8:ofab480.](https://sciwheel.com/work/bibliography/15141359)

[77. Ye S, Kronish I, Fleck E, Fleischut P, Homma S, Masini D, et al. Telemedicine Expansion During the COVID-19 Pandemic and the Potential for Technology-Driven Disparities. J Gen Intern Med. 2021;36:256–8.](https://sciwheel.com/work/bibliography/10575804)
