# Supplementary material for: Video-based telemedicine utilization patterns and associated factors among racial and ethnic minorities in the United States during the COVID-19 pandemic: A mixed-methods scoping review
Source: PLOS Digit Health. 2025 Jul 24;4(7):e0000952. doi: 10.1371/journal.pdig.0000952 (PMC12289041; doi:10.1371/journal.pdig.0000952)
Supplement: S2 File — (DOCX) [file pdig.0000952.s002.docx]

**Master coding index:** Thematic barriers to telemedicine adoption

| **Individual** | **Provider** | **Organizational** | **Community** | **Policy** |
| --- | --- | --- | --- | --- |
| Digital and healthcare access  Traditional, health, and digital literacy  Insurance status  Personal and language preferences  Disease severity and immobility  Loss of trust  **Intersectional**  **Barriers**  Race and ethnicity, age, insurance status, language preference, and  comorbidities | Attitudes and perceptions of telemedicine  Comfort and proficiency using telemedicine  Workflow practices  Cultural competencies  Implicit or explicit biases | Telemedicine and resource availability  Capacity for robust telemedicine implementation  Technological challenges  Systemic racism  Training and Education  Culturally and structurally appropriate digital tools | Low SES and income  Digital access and infrastructure  Cultural and religious preferences  Geographical location  Social support | Payor policies and practices  Ineffective digital expansion policies  Standardized protocols |

Thematic facilitators of telemedicine adoption

| **Individual** | **Provider** | **Organizational** | **Community** | **Policy** |
| --- | --- | --- | --- | --- |
| Enhanced awareness of telemedicine  Increased trust and confidence in digital health tools  Improved self-efficacy and an intrinsic desire to use telemedicine    Enhanced digital literacy skills  Access to patient portal communication | Education and training in telemedicine  Improved provider representativeness and competencies | Outreach and education  Ongoing development of digital and telemedicine infrastructure  Digital literacy training in virtual platforms  Integration of culturally and linguistically adapted digital tools and technologies  Robust telemedicine support team  User testing of new digital technologies with end-user involvement  Cross-sectoral collaborations to enhance digital equity | Increased digital equity channels, networks, and organizations  Improved digital infrastructure  Increased outreach efforts by local community leaders and health workers    Cross-sectoral collaborations to enhance digital equity | Increase subsidization of digital tools and technologies  Enhance provider representativeness  Continue telephone- and video-based telemedicine reimbursement  Cross-sectoral collaborations to enhance digital equity |
