# Supplementary material for: Video-based telemedicine utilization patterns and associated factors among racial and ethnic minorities in the United States during the COVID-19 pandemic: A mixed-methods scoping review
Source: PLOS Digit Health. 2025 Jul 24;4(7):e0000952. doi: 10.1371/journal.pdig.0000952 (PMC12289041; doi:10.1371/journal.pdig.0000952)
Supplement: S3 File — (DOCX) [file pdig.0000952.s003.docx]

**Preferred Reporting Items for Systematic reviews and Meta-Analyses extension for Scoping Reviews (PRISMA-ScR) Checklist**

| **SECTION** | **ITEM** | **PRISMA-ScR CHECKLIST ITEM** | **REPORTED ON PAGE #** |
| --- | --- | --- | --- |
| **TITLE** | | | |
| Title | 1a | Video-Based Telemedicine Utilization Patterns and Associated Factors among Racial and Ethnic Minorities in the United States During the COVID-19 Pandemic: A Mixed-Methods Scoping Review | 1 |
| **AUTHORS** | | | |
| Authors | 1b | John M Meddar  Ratnalekha Viswanadham  Defne L.Levine  Tiffany Martinez  Kendra Willis  Noah Choi  Jackson Douglas  Katharine Lawrence |  |
| **ABSTRACT** | | | |
| Structured summary | 2 | Objective This mixed-methods scoping review quantitatively and qualitatively characterizes patterns of telemedicine utilization among racial/ethnic minorities in the United States during the COVID-19 pandemic. Materials and Methods A comprehensive search across several databases was performed between 2020 and 2023 to identify relevant literature that examined telehealth and telemedicine utilization, particularly focusing on video visit utilization among racial/ethnic minorities. A team of reviewers screened, reviewed, and charted key information related to the research aim. . Results Of 1801 studies screened, 77 studies met our criteria for inclusion. We observed increased uptakes of telemedicine among racial/ethnic minorities across 31 studies (40.3%), mixed uptake across 15 studies (19.5%), and decreased uptake across 29 studies (37.6%). Most studies assessed disparities among non-Hispanic Black and Hispanic/Latinx populations (73 and 66 studies, respectively), while fewer examined disparities among Asian and other minority populations (up to 45 studies). Thematic barriers to telemedicine adoption were identified at varying socio-ecological levels of care. Identified barriers were mainly centered around limited digital access and literacy at the patient and community levels, inadequate digital capacity and infrastructure at the organizational level, implicit bias and telemedicine proficiency at the provider level. Conclusion This scoping review highlights that telemedicine adoption was heterogeneous across race/ethnic minority populations and suggests that differences in uptake were influenced by both disparities-related conditions as well as non-disparities-related factors. Disparities were most evident among Asian, Native American/Alaska Native, and Native Hawaiian/Pacific Islander groups. | 2 |
| **INTRODUCTION** | | | |
| Rationale | 3 | Rapid digital expansion in healthcare during the pandemic produced widespread changes how healthcare is accessed and utilized. However, there is limited research evaluating the impact of these changes on telemedicine adoption practices among racial/ethnic minorities and prevailing barriers and facilitators that impact telemedicine uptake. | 3 |
| Objectives | 4 | To comprehensively assess the COVID-19 pandemic literature concerning patterns of telemedicine adoption among racial/ ethnic minorities during the covid-19 pandemic. We also aim to qualitatively evaluate barriers and facilitators that influence telemedicine adoption practices. | 3 |
| **METHODS** | | | |
| Protocol and registration | 5 | https://osf.io/dvfcz/?view_only= | 4 |
| Eligibility criteria | 6 | - Study designs: Observational analyses, case reports, mixed methods papers (for quantitative sections), cross-sectional studies, panel studies, survey studies - Studies with patients of all ages - U.S-based studies published between 2020 and 2023 - Any demographic setting (rural, urban, or suburban) - All practice types. - All sexes and genders - Studies were only eligible for inclusion if they offered remote provision of care (patient-provider interactions) using telecommunication and/or information technology, synchronously. - Patient-provider interfacing studies that utilized video or tele-video conferencing or a combination of video and other forms of synchronous or asynchronous modalities | 4-5 |
| Information sources* | 7 | Literature searches were conducted in the highlight databases below.   - **PubMed** - **Embase** - **Cochrane Library** - **Web of Science (all dbs)** - **CINAHL** - **Engineering village** | 7-8 |
| Search | 8 | (Alabama OR Alaska OR Appalachian Region OR Arizona OR Arkansas OR California OR Los Angeles OR San Francisco OR Connecticut OR Delaware OR District of Columbia OR Florida OR Georgia OR Great Lakes Region OR Hawaii OR Idaho OR Illinois OR Chicago OR Indiana OR Iowa OR Kansas OR Kentucky OR Louisiana OR New Orleans OR Mississippi OR Maine OR Maryland OR Massachusetts OR Boston OR Michigan OR Mid-Atlantic Region OR Midwestern United States OR Minnesota OR Missouri OR Montana OR Nebraska OR Nevada OR New England OR New Hampshire OR New Mexico OR New York OR New York City OR Ohio OR North Carolina OR North Dakota OR Northwestern United States OR Ohio OR Oklahoma OR Oregon OR Pacific States OR Pennsylvania OR Philadelphia OR Rhode Island OR South Carolina OR South Dakota OR Southeastern United States OR Southwestern United States OR Tennessee OR Texas OR United States OR "USA" OR Utah OR Vermont OR Virginia OR Washington OR West Virginia OR Wisconsin OR Wyoming) AND    (Covid-19 OR Coronavirus disease 19 OR 2019-nCOV OR SARS-cov-2 infection OR corona virus)    AND    (racial minorities OR ethnicity OR ethnic groups OR nationality OR population groups OR hispanic OR latino* OR latina* OR racial group* OR Asian american* OR american indian* OR native american* OR alaska native* OR alaskan native* OR native hawaiian OR Pacific islander OR african american* OR black american* OR caribbean american* OR afro-american* OR latinx OR cuban american* OR spanish american* OR US latin american* OR mexican american* OR ethnic OR disparit* OR inequalit* OR medically underserved OR minorit* OR BIPOC OR race factors OR discrimination OR cultural competenc*)    AND    (utilized utilize OR utilization OR primary care access OR patient acceptance of health care OR delivery of health care OR Health equity OR healthcare disparities OR practice patterns OR facilities utilization OR services utilization OR healthcare utilization OR acceptance of healthcare OR health care utilization OR acceptance of health care OR health care seeking behavior OR healthcare seeking behavior OR acceptability of health care OR acceptability of healthcare OR patient participation)    AND    (telemedicine OR telehealth OR e-medicine OR e-health OR emedicine OR ehealth OR virtual medicine OR tele-referral OR tele-icu OR mobile health OR mhealth OR remote consultation) | 8 |
| Selection of sources of evidence† | 9 | Sources of evidence will be selected across 3 successive phases of screening and data extraction. The first 2 phases are the title and abstract and full text screening phases. During these phases, we will screen all selected articles to identify a final pool of eligible citations for analysis. The third and final phase is the data extraction phase where data will be extracted from final round of eligible studies into our extraction tool. | 8 |
| Data charting process‡ | 10 | Data will be charted on a calibrated charting tool provided by Covidence, a Cochrane technology platform, designed to screen and chart data from studies. Charting will be performed with consensus among reviewers and interrater reliability calculated | 8 |
| Data items | 11 | - Author/ Publication year - Study Characteristics (Design/Duration/Npatients/Nclinicans/time period) - Telemedicine Method: (telehealth or telemedicine/combinational/Video visit) - Utilization indicators by race and ethnicity (across video, telephone and in-person visits) - Minority Group(s) included in the study - Clinical specialty - Geographical region/state) - Qualitative evaluation of barriers and facilitators - Conclusion | 8-9 |
| Critical appraisal of individual sources of evidence§ | 12 | There will be no critical appraisals conducted in this scoping review. | x |
| Synthesis of results | 13 | We will analyze a sample of studies to ascertain compositional consistencies across study populations or by racial demographic breakdowns in the US. After doing this we’ll assess utilization proportions by race. If patterns are not recognizable, we will quantitatively characterize between group and within-group proportional differences in utilization across three modalities of care: video, telephone, and in-person visits. | 9 |
| **RESULTS** | | | |
| Selection of sources of evidence | 14 | Studies from database and manual screening = 1801  References remove = 572  Studies screened 1229 -> studies excluded = 873  Studies sought for retrieval 356 -> studies not retrieved n = 0  Studies assessed for eligibility 356 -> studies excluded 279   - Unable to find n = 11 - International study n = 1 - Telephone only n = 2 - Abstract/No manuscript n = 27 - Does not stratify by race/ethnicity n = 44 - Does not compare visit modalities n = 104 - Study is not within specified time window n = 4 - Study does not assess disparities n = 11   Studies included in review n = 77 | 11 |
| Characteristics of sources of evidence | 15 | Study characteristics reflect the data points listed above. Given the quantity of studies reviewed, please contact the corresponding author for a full reference list. | x |
| Critical appraisal within sources of evidence | 16 | Critical appraisal of studies was not performed. | x |
| Results of individual sources of evidence | 17 | A table with the full list of studies included in the review can be found in the supplementary materials. | x |
| Synthesis of results | 18 | A total of 1,786 studies were identified in the initial search, downloaded into Covidence, and screened by reviewers. Fifteen studies were manually screened by reviewers and included in the review. Of these, 77 studies met our inclusion criteria. A majority of papers analyzed patients (n = 53, aggregate patient count: n = n = 7,937,340). A significant number analyzed clinical encounters (n = 42, aggregate encounter count: n = 6,963,693). Racial/ethnic minorities breakdown across included studies were: non-Hispanic/Latinx Black (n = 73), Hispanic/Latinx (n = 66), Asians (n = 45), Native American and Alaskan Native (NA/AN) (n = 18), and Native Hawaiian and Pacific Islander (NH/PI) (n = 13). A majority of studies had a cohort design (n = 46). A minority of studies had a cross-sectional design (n = 31). Across all studies, the average length of the study period was 8.12 months (SE = 0.79 months) and a median of 6.30 months (IQR (3.30, 10.0)). A minority of studies collected data before and during the acute COVID-19 pandemic We observed varied definitions used for telemedicine and telehealth. A slight majority of studies defined remote service delivery using telecommunication technologies as telehealth (n =36 ). We observed increased uptakes of telemedicine among racial/ethnic minorities across 33 studies (42.9%), mixed uptake across 15 studies (20%), and decreased uptake across 29 studies (37.7%). Most studies assessed disparities among non-Hispanic Black and Hispanic/Latinx populations (73 and 66 studies, respectively), while fewer examined disparities among Asian and other minority populations (up to 45 studies). Thematic barriers to telemedicine adoption were identified at varying socio-ecological levels of care. Identified barriers were mainly centered around limited digital access and literacy at the patient and community levels, inadequate digital capacity and infrastructure at the organizational level, implicit bias and telemedicine proficiency at the provider level. | 9-10 and 20 |
| **DISCUSSION** | | | |
| Summary of evidence | 19 | Despite efforts to address disparities caused by telemedicine expansion during the pandemic, our analysis of visit dynamics across multiple care modalities shows decreased telemedicine uptake, corresponding to greater use of in-person visit utilization by racial/ethnic minority groups. While study heterogeneity makes drawing between study comparisons challenging, proportional differences in utilization likely reflect existing barriers, driven by a host of geographically distinct and patient population-specific factors, including socioeconomic standing, digital and infrastructural inequities, and provider and systems-level barriers that are influenced by the wider public health, policy, and digital infrastructure landscapes  Across race/ethnicity groups, Asians, NA/AN, and NH/PI had a greater proportion of studies that showed decreased telemedicine uptake relative to in-person uptake, which may reflect a lack of tailoring of service delivery to accommodate the unique needs of these population groups. This is likely due to their limited population composition relative to Hispanic/Latinx and non-Hispanic Black populations. These findings align with existing research showing that Asians, NA/AN, and NH/PI groups experience compounding barriers to accessing healthcare, including cultural, language , geographic, discrimination-related barriers.  A predominant barrier at the individual level was digital inequities, including lack of access to quality internet and broadband connection, and limited access to digital devices such as smartphones, laptops, computers, and tablets. Since digital inclusion is considered a super social determinant of health, particularly for racial/ethnic minorities and rural populations, increased and targeted efforts are needed to improve access and utilization for these populations.  Widely reported barriers to telemedicine utilization reflected systemic racism and trust in providers. While evidence on the impact of racism in virtual interactions is limited, the enduring legacy of systemic racism, having persisted throughout the decades, is shown to reduce minority individuals’ willingness to engage with the healthcare delivery system. Healthcare systems and organizations committed to addressing these barriers should consider enacting various community-based telemedicine initiatives with the dual aim of establishing goodwill, and fostering a sense of trust and connectedness while also providing proactive solutions to digital inequities, including improving telemedicine awareness, assessing end-user needs through participatory design processes for telemedicine and digital tool development, and providing digital literacy training. These efforts should be conducted in parallel with rigorous and extensive anti-bias training, thereby strengthening community-institution partnerships and improving patient engagement. | 20-22 |
| Limitations | 20 | This review has several limitations. First, there was little consensus in how studies defined "telehealth" and "utilization," thereby complicating comparisons and potentially inflating utilization estimates. To address this, we broadened our search terms. Additionally, the inconsistent or interchangeable use of the terms *telehealth* and *telemedicine* across studies posed a challenge for synthesis. Some studies used the terms synonymously, while others applied them to different modalities (e.g., video only versus video and telephone combined). This definitional ambiguity could have contributed to heterogeneity in findings and limited the clarity of modality-specific uptake trends. Future work would benefit from standardized definitions to facilitate more precise comparisons across studies. Second, many included studies were cross-sectional, and a large fraction had short study duration periods. These limitations restricted our ability to infer causality and assess longitudinal patterns of adoption among racial/ethnic minorities. Though high satisfaction with telemedicine was reported during the pandemic, recent data suggest a resurgence of in-person care, further highlighting the need for future research on post-pandemic acceptance of technology. Third, our review focused on synchronous telemedicine modalities, excluding asynchronous communication methods (e.g., email, secure messaging, patient portal). While synchronous formats are central to routine care delivery, excluding asynchronous modalities limits our capacity to more thoroughly assess the extent of telemedicine use disparities across all telemedicine formats. Fourth, a single coder performed the qualitative analysis; although thematic content was clear and consistent, subjective interpretation may have introduced bias. A single coder also extracted some data elements outside our Covidence workflow, which could have also introduced bias. Fifth, the exclusion of RCTs may have negatively impacted our study findings since we did not include rigorous comparisons of telemedicine adoption interventions. This imposed a challenge to establishing a causal link between telemedicine utilization patterns and sociodemographic disposition. Sixth, the review was restricted to U.S.-based studies. While findings from other geographical contexts may provide novel insights into minority telemedicine use patterns and associated factors, the unique complexities of the American healthcare delivery system, including its provider payment systems, extensive heterogeneity of the general U.S. population, and prevailing structural inequities, warrant focused examination. Seventh, only English-language, peer-reviewed studies were included, which may have limited the generalizability of our study. Finally, following PRISMA-ScR guidelines, we did not formally appraise study quality, which was consistent with scoping review conventions. However, studies examined in this review may be subject to several methodological limitations, including missing data from informal and unstructured EHR data collection (i.e., text messages, unstructured documentation, or undocumented phone calls), leading to underreported utilization estimates. Populations considered digitally disenfranchised, such as racial/ethnic minorities and geographically rural populations, may be significantly underrepresented in analyses due to digital access barriers, including limited digital literacy, inadequate internet access, and lack of device ownership. This has important implications for the internal and external validity of the studies under review. Cohort studies may be limited by the inability to assess the temporal influences that underlie technology utilization patterns, such as policy, COVID-19 variants, or rapid technology diffusion in response to pandemic-induced restrictions. | 22 |
| Conclusions | 21 | There was a significant upsurge in studies exploring telemedicine utilization during the COVID-19 pandemic. Findings from this work demonstrated that patterns of telemedicine use were heterogeneous across racial and ethnic minority populations in reported studies, suggesting that differences in uptake were likely influenced by disparities-related conditions, including socioeconomic status (SES) and technological barriers, as well as related factors such as availability and provider capability. Efforts to address inequitable access to telemedicine for race/ethnic minorities and other vulnerable populations will require a robust understanding of a constellation of factors that are involved in telemedicine use, including addressing barriers at every socio-ecological level of care. | 22 |
| **FUNDING** | | | |
| Funding | 22 | Mr. Meddar is supported by the Mark D Schwartz (T32HS026120-05S10) for the Agency of Healthcare Quality and Research. Dr. Viswanadham is supported by a Ruth L. Kirschstein Award (T32HP22238) from the Health Resources and Services Administration. | 23 |

JBI = Joanna Briggs Institute; PRISMA-ScR = Preferred Reporting Items for Systematic reviews and Meta-Analyses extension for Scoping Reviews.

* Where *sources of evidence* (see second footnote) are compiled from, such as bibliographic databases, social media platforms, and Web sites.

† A more inclusive/heterogeneous term used to account for the different types of evidence or data sources (e.g., quantitative and/or qualitative research, expert opinion, and policy documents) that may be eligible in a scoping review as opposed to only studies. This is not to be confused with *information sources* (see first footnote).

‡ The frameworks by Arksey and O’Malley (6) and Levac and colleagues (7) and the JBI guidance (4, 5) refer to the process of data extraction in a scoping review as data charting*.*

§ The process of systematically examining research evidence to assess its validity, results, and relevance before using it to inform a decision. This term is used for items 12 and 19 instead of "risk of bias" (which is more applicable to systematic reviews of interventions) to include and acknowledge the various sources of evidence that may be used in a scoping review (e.g., quantitative and/or qualitative research, expert opinion, and policy document).

*From:* Tricco AC, Lillie E, Zarin W, O'Brien KK, Colquhoun H, Levac D, et al. PRISMA Extension for Scoping Reviews (PRISMAScR): Checklist and Explanation. Ann Intern Med. 2018;169:467–473. [doi: 10.7326/M18-0850](http://annals.org/aim/fullarticle/2700389/prisma-extension-scoping-reviews-prisma-scr-checklist-explanation).
