# Supplementary material for: Video-based telemedicine utilization patterns and associated factors among racial and ethnic minorities in the United States during the COVID-19 pandemic: A mixed-methods scoping review
Source: PLOS Digit Health. 2025 Jul 24;4(7):e0000952. doi: 10.1371/journal.pdig.0000952 (PMC12289041; doi:10.1371/journal.pdig.0000952)
Supplement: S4 File — (DOCX) [file pdig.0000952.s004.docx]

### Search Strategy for Ovid MEDLINE: Epub Ahead of Print, In-Process & Other Non-Indexed Citations, Ovid MEDLINE Daily and Ovid MEDLINE 1946-Present

Date Run: October, 2021
Search Strategy:

| **#** | **Searches** | **Results** |
| --- | --- | --- |
| 1 | exp Telecommunications/ or internet-based intervention/ | 91461 |
| 2 | (remot* adj2 (consult* or interact* or diagnos* or monitor* or treat* or therap* or care)).tw,kw,kf. | 5854 |
| 3 | (telemonitor* or telemedicine* or telecommunication* or telehealth*).tw,kf. | 21131 |
| 4 | ((remote or online or video* or text message* or telephone* or phone or phones or email* or virtual* or technolog* or iphone* or smartphone* or mobile application* or mobile app* or teleconferenc* or messenger or whatsapp or skype or zoom or instant messag* or tablet* or e-mail* or asynchronous messag* or synchronous messag* or Videoconferenc* or hotline* or helpline* or call center*) adj3 (communicat* or engag* or discuss* or care or interact* or clinical guidance)).tw,kf,kw. and patient*.tw. | 11768 |
| 5 | ((remote or online or electronic* or video* or text message* or telephone* or phone* or email* or technolog* or virtual* or iphone* or smartphone* or mobile application* or mobile app* or teleconferenc* or messenger or whatsapp or skype or zoom or instant messag* or tablet* or e-mail* or asynchronous messag* or synchronous messag* or Videoconferenc* or hotline* or helpline* or call center*) adj3 (consult* or appointment* or meet or meeting* or visit*)).tw,kw,kf. or virtual tool*.tw,kf. | 8718 |
| 6 | ((virtual* or digital*) adj3 (healthcare or health care or health strategy)).tw,kw,kf. or (virtual care or virtual health).tw,kf. or (rapid* adj3 virtual*).tw,kf. | 1464 |
| 7 | ((online or digital* or virtual*) adj3 (doctor* or physician* or clinic or clinics or nurse or nurses or nursing or medicine or medical)).tw,kw,kf. | 5110 |
| 8 | (digital health or digital first).tw,kf. | 1853 |
| 9 | ("e health*" or ehealth* or evisit* or "e-visit*").tw,kf. | 6656 |
| 10 | (online adj3 (healthcare or health care)).tw,kf,kw. | 389 |
| 11 | ((virtual* or digital*) adj3 (healthcare or health care or health strategy)).tw,kf,kw. | 709 |
| 12 | telebased or "tele‐based" or telecancer or "tele‐cancer" or "tele‐cardiolo*" or telecardiolog* or teleconsult* or "tele‐consult*" or telecounselling or "tele‐counselling" or telecounseling or "tele‐counseling" or teledental or "tele‐dental" or telederm* or "tele‐derm*" or telediagnos* or "tele‐diagnos*" or teledialysis or "tele‐dialysis" or teleecho* or "tele‐echo*" or teleemerg* or "tele‐emerg*" or teleepileps* or "tele‐epileps*" or telefollow* or "tele‐follow*" or teleguidance or "tele‐guidance" or "tele‐health*" or telehome* or "tele‐home*" or teleICU or "tele‐ICU" or teleintervention* or "tele‐intervention*" or telemanag* or "tele‐manag*" or telemedicine or "tele‐medicine" or telemonitor* or "tele‐monitor*" or telenurs* or "tele‐nurs*" or teleoncolo* or "tele‐oncolo*" or teleopthalm* or "tele‐opthalm*" or telepalliat* or "tele‐palliat*" or "tele‐patholog*" ).tw,kf. | 23364 |
| 13 | (tele adj (care or counselling or counseling or diagnos* or health* or intervention* or medicine or medical or nursing)).tw,kw. | 384 |
| 14 | ("e‐care" or ecare or "e‐consult*" or econsult* or "e‐diagnos*" or ediagnosis* or "e‐medicine" or emedicine or "e‐nurse*" or enurse* or "e‐nursing" or enursing or "e‐physician*" or ephysician* tw,kf. | 4823 |
| 15 | ((online or video* or text message* or telephone* or phon* or email* or virtual* or technolog* or iphone* or smartphone* or mobile application* or mobile app* or teleconferenc* or messenger or instant messag* or whatsapp or skype or zoom or tablet* or e-mail* or asynchronous messag* or synchronous messag* or Videoconferenc* or hotline* or helpline* or call center*) adj2 (care or counselling or Counseling or diagnos* or health* or intervention* or manag* or therap* or treat* or medicine or medical or nursing)).tw,kw,kf. | 63528 |
| 16 | (virtual* adj3 monitor*).tw,kw,kf. | 139 |
| 17 | ((implant* sensor* or body sensor*) adj4 (diagnost* or monitor* or report*)).tw,kf. | 70 |
| 18 | mobile health monitor*.tw,kf. | 37 |
| 19 | computers, handheld/ or smartphone/ | 7796 |
| 20 | Mobile Applications/ | 5800 |
| 21 | exp Wearable Electronic Devices/ | 11582 |
| 22 | Computers/ or exp Microcomputers/ or Minicomputers/ | 69847 |
| 23 | internet/ or internet access/ or "internet of things"/ | 72614 |
| 24 | or/1-23 [telemedicine 1] | 316750 |
| 25 | Health Equity/ or (health adj3 (equit* or disparit*)).tw,kf. or Health Status Disparities/ | 34265 |
| 26 | communication barriers/ or digital divide/ or limited english proficiency/ | 6597 |
| 27 |  | 6921 |
| 28 | exp culture/ or (cultur* adj3 (communit* or person* or population* or disparit* or inequalit* or equity or factor* or ident*)).tw,kf,kw. | 192237 |
| 29 | Minority Groups/ or Minority Health/ | 14302 |
| 30 | (minorit* adj3 (group* or population* or communit* or people* or person* or patient* or health)).tw,kf,kw. | 23943 |
| 31 | Race Factors/ | 178 |
| 32 | exp Continental Population Groups/ or exp Ethnic Groups/ | 293023 |
| 33 | (ethnic* adj3 (group* or communit* or person* or patient* or population* or disparit* or inequalit* or equity or ident*)).tw,kw,kf. | 59202 |
|  |  | 242845 |
| 35 | exp Socioeconomic Factors/ or "Social Determinants of Health"/ | 449665 |
| 36 |  | 68936 |
| 37 | sociological factors/ | 555 |
| 38 | (socioeconomic adj2 (factor* or disadvantag*)).tw,kw,kf. | 21342 |
| 39 | "social determinants of health".tw,kf. | 5054 |
| 40 | (marital status or education status or salaries or salary).tw,kf. | 29253 |
| 41 | (low income or working poor or poverty or precarious work or homeless* or income or employment or employed or economic status or salar* or pension* or career mobility or ghetto* or food insecurit*).tw,kf. | 555932 |
| 42 | (street* adj2 (people* or person* or individual* or population*)).tw,kf. | 1100 |
| 43 | (street* adj2 (people* or person* or individual* or youth* or population* or child* or men or women or man or woman)).tw,kf. | 20113 |
|  | ((vulnerabl* or transient*) adj2 (people* or person* or individual* or child* or youth* or population* or worker* or men or women or man or woman or communit*)).tw,kf. |  |
| 44 | ("lack of housing" or substandard housing or unstabl* house* or underhoused or under housed or squatter*).tw,kf. | 691 |
| 45 | homeless persons/ | 8868 |
| 46 | Working Poor/ | 15 |
| 47 | exp social discrimination/ or social marginalization/ or social stigma/ | 14414 |
| 48 | (social adj3 (class or mobilit* or margin* or status or condition* or exclusion* or inequit* or equity or discriminat* or segretat*)).tw,kw. or social capital.tw,kf. | 34435 |
| 49 | Vulnerable Populations/ | 10424 |
| 50 | ((vulnerabl* or underserv* or disadvantag* or uninsured) adj3 (group* or population* or communit* or people* or person* or youth* or patient* or child* or men or man or woman or women)).tw,kw,kf. | 46060 |
| 51 | Refugees/ | 10122 |
| 52 | (refugee* or Asylum Seeker* or displaced person* or asylee*).tw,kf. | 11865 |
| 53 | "Emigration and Immigration"/ | 25126 |
| 54 | "Transients and Migrants"/ | 11542 |
| 55 | (immigrant* or immigration* or emigrant* or emigration*).tw,kf. | 37780 |
| 56 | (migrant* or incomer* or "in comer*" or "new comer*" or newcomer*).tw,kf. | 21136 |
| 57 | Prisoners/ | 16488 |
| 58 | Criminals/ | 4880 |
| 59 | (inmate* or jail or prisoner* or criminal*).tw,kf. | 35348 |
| 60 | Sex Workers/ or Sex Work/ | 7589 |
| 61 | (prostitut* or sex worker*).tw,kf. | 9079 |
| 62 |  | 4644 |
| 63 |  | 278222 |
| 64 |  | 207156 |
| 65 |  | 9792 |
| 66 |  | 143659 |
| 67 |  | 20014 |
| 68 |  | 2537 |
| 69 |  | 97727 |
| 70 |  | 108 |
| 71 |  | 156902 |
| 72 |  | 86647 |
| 73 |  | 71698 |
| 74 |  | 5362 |
| 75 |  | 41484 |
| 76 |  | 312851 |
| 77 |  | 546 |
| 78 |  | 42607 |
| 79 | Rural Health/ | 23349 |
| 80 | exp Rural Health Services/ | 12833 |
| 81 | Rural Population/ | 58942 |
| 82 | ((rural or remote) adj3 (group* or population* or communit* or people* or men or man or women or woman or person* or patient* or equity or disparit* or inequalit* or equity)).tw,kw,kf. | 52952 |
| 83 | exp disorders of sex development/ | 33057 |
| 84 | Health Services for Transgender Persons/ | 133 |
| 85 | exp Sexuality/ | 41445 |
| 86 | exp "Sexual and Gender Minorities"/ | 6163 |
| 87 | exp Gender Identity/ | 19575 |
| 88 | Gender Dysphoria/ | 468 |
| 89 | ((gender* or sexual*) adj3 (fluid* or minorit* or identit* or diverse or confirmation or non conform* or surg* or reassignment or change or disorder* or group* or population* or communit* or people* or person* or patient* or orientation)).tw,kw,kf. | 67419 |
| 91 |  | 3104139 |
| 92 |  | 43023 |
| 93 |  | 17659 |
| 94 |  | 844248 |
| 95 |  | 146858 |
| 96 |  | 6820 |
| 97 |  | 16733 |
| 98 |  | 1302 |
| 99 |  | 36152 |
| 100 |  | 3258 |
| 101 |  | 64577 |
| 102 |  | 355 |
| 103 |  | 411 |
| 104 |  | 415 |
| 105 | (Native* adj1 (American* or man or men or women or adult or people* or Indian* or Nation or tribe* or tribal or band or bands)).tw,kf,kw. | 5861 |
| 106 | Alaska Native*.tw,kf. | 2747 |
| 107 | turtle island.tw,kf. | 7 |
| 108 | (urban adj3 (Indian* or Native*)).tw,kf,kw. | 1117 |
| 109 | (indian* adj3 (north americ* or american*)).tw,kw,kf. | 7710 |
| 110 |  | 6026001 |
| 111 |  | 11058 |
| 112 |  | 218860 |
| 113 |  | 178572 |
| 114 |  | 1246 |
| 115 |  | 6832 |
| 116 |  | 592 |
| 117 |  | 554 |
| 118 |  | 3004 |
| 119 |  | 1278 |
| 120 |  | 36962 |
| 121 |  | 3788 |
| 122 |  | 10926 |
| 123 |  | 19505 |
| 124 |  | 211295 |
| 125 |  | 61 |
| 126 |  | 172 |
| 127 |  | 105 |
| 128 |  | 12 |
| 129 |  | 354 |
| 130 |  | 66 |
| 131 |  | 183 |
| 132 |  | 72 |
| 133 |  | 211 |
| 134 |  | 98338 |
| 135 |  | 31 |
| 136 |  | 3405 |
| 137 |  | 416561 |
| 138 |  | 110 |
| 139 |  | 29710 |
| 140 | (medline or pubmed or embase or psychlit or psyclit or psychinfo or psycinfo or cinahl or cinhal or science citation index or scopus or "web of science" or reference list* or bibliograph* or hand-search* or handsearch* or relevant journal* or manual search* or Covidence or DistillerSR or Rayyan or prospero or deduplication or de-duplication or reference manager* or endnote or refworks or zotero or mendeley or prisma or joanna briggs or revman or EPPI-Reviewer or SysRev).ab. | 248166 |
| 141 | (selection criteria or eligibility criteria or screening criteria or inclusion criteria or exclusion criteria).ab. | 147327 |
| 142 | (mantel haenszel or peto or der simonian or dersimonian or fixed effect* or latin square* or outcomes research or relative effectiveness).ab. | 32487 |
| 143 |  | 179789 |
| 144 |  | 12940 |
| 145 |  | 20112 |
| 146 |  | 524074 |
| 147 |  | 2774447 |
| 148 |  | 190835 |
| 149 |  | 484252 |
| 150 |  | 4042 |
| 151 | 2015* or 2016* or 2017* or 2018* or 2019* or 2020 or 2021*).dt,ez,da,dp. | 15833687 |
| 152 | 150 and 151 | 3713 |
