# Supplementary material for: Video-based telemedicine utilization patterns and associated factors among racial and ethnic minorities in the United States during the COVID-19 pandemic: A mixed-methods scoping review
Source: PLOS Digit Health. 2025 Jul 24;4(7):e0000952. doi: 10.1371/journal.pdig.0000952 (PMC12289041; doi:10.1371/journal.pdig.0000952)
Supplement: S7 File — (DOCX) [file pdig.0000952.s007.docx]

Color codes:

Red: conflicting findings

Bolded: Information relevant to the analysis

Brown: Interesting and potentially relevant

Grey: Important to note

Study 1. Bustamante et al. 2022

Link: <https://www.frontiersin.org/journals/public-health/articles/10.3389/fpubh.2023.1222203/full>

**Introduction:** Telehealth can potentially improve the quality of healthcare through increased access to primary care. While telehealth use increased during the COVID-19 pandemic, racial/ethnic disparities in the use of telemedicine persisted during this period. Little is known about the relationship between health coverage and patient race/ethnicity after the onset of the COVID-19 pandemic.

**Objective**: This study examines how differences in patient race/ethnicity and health coverage are associated with the number of in-person vs. telehealth visits among patients with chronic conditions before and after California's stay-at-home order (SAHO) was issued on 19 March 2020.

**Methods:** We used weekly patient visit data (in-person (N = 63, 491) and telehealth visits (N = 55, 472)) from seven primary care sites of an integrated, multi-specialty medical group in Los Angeles County that served a diverse patient population between January 2020 and December 2020 to examine differences in telehealth visits reported for Latino and non-Latino Asian, Black, and white patients with chronic conditions (type 2 diabetes, pre-diabetes, and hypertension). After adjusting for age and sex, we estimate differences by race/ethnicity and the type of insurance using an interrupted time series with a multivariate logistic regression model to study telehealth use by race/ethnicity and type of health coverage before and after the SAHO. A limitation of our research is the analysis of aggregated patient data, which limited the number of individual-level confounders in the regression analyses.

**Results:** Our descriptive analysis shows that telehealth visits increased immediately after the SAHO for all race/ethnicity groups. Our adjusted analysis shows that the likelihood of having a telehealth visit was lower among uninsured patients and those with Medicaid or Medicare coverage compared to patients with private insurance. **Latino and Asian patients had a lower probability of telehealth use compared with white patients.**

**Discussion**

One of our study's main strengths is that the sample includes a diverse number of patient visits from the main race/ethnic groups in California. However, since our study was conducted in a specific commercial group, it is not fully representative of the state's population or of individuals who receive care at other health systems, which limits the generalizability of our findings. Another strength is that we illustrate telehealth utilization across different categories of race/ethnicity and insurance in a commercial setting, while previous studies only provided findings on resource-scarce settings.

A limitation of this study is the use of aggregate patient visit data rather than individual patient-level data. The use of aggregate patient visit data limits the ability to determine patient-level associations with clinical demographics and/or other variables, such as age, gender, and language use at each primary care visit. However, we were able to operationalize age and gender as a proportion at the clinic level. Although individual patient-level data are preferable and are more flexible for statistical analysis, aggregated data have been shown to be appropriate for statistical analysis when patient-level data are unavailable ([29](https://www.frontiersin.org/journals/public-health/articles/10.3389/fpubh.2023.1222203/full#B29), [30](https://www.frontiersin.org/journals/public-health/articles/10.3389/fpubh.2023.1222203/full#B30)). **Moreover, this study did not address other potential barriers that underrepresented minority groups could experience while accessing virtual care, such as limited access to technology-enabled resources faced during the pandemic (**[**5**](https://www.frontiersin.org/journals/public-health/articles/10.3389/fpubh.2023.1222203/full#B5)**,** [**8**](https://www.frontiersin.org/journals/public-health/articles/10.3389/fpubh.2023.1222203/full#B8)**,** [**11**](https://www.frontiersin.org/journals/public-health/articles/10.3389/fpubh.2023.1222203/full#B11)). In this study, we were unable to disaggregate access to telehealth visits due to COVID-19 or other health conditions. Additional limitations of our study are the potential for selection bias and unmeasured factors such as socioeconomic status and other confounders.

In this study, we sought to examine the relationship between patient race/ethnicity and health coverage, with telehealth visits among patients with chronic conditions before and after California's SAHO, **issued on 19 March 2020.** Our results confirm that as the norm was across primary care in the US prior to the pandemic, most visits by patients with chronic conditions were conducted in person before the SAHO, and there was an immediate shift to telehealth use once the SAHO was enacted. Thus, our findings are consistent with the existing literature on telehealth, accounting for a low percentage of healthcare delivery before the COVID-19 pandemic ([5](https://www.frontiersin.org/journals/public-health/articles/10.3389/fpubh.2023.1222203/full#B5), [8](https://www.frontiersin.org/journals/public-health/articles/10.3389/fpubh.2023.1222203/full#B8), [11](https://www.frontiersin.org/journals/public-health/articles/10.3389/fpubh.2023.1222203/full#B11)). **When examining telehealth use by race/ethnicity, we found that telehealth visits by Black patients exceeded visits by white patients, irrespective of the type of health insurance coverage**. This finding supports the results from previous findings, **where interviewees perceived that Black and white patient populations experienced fewer technology hurdles with telehealth use** ([5](https://www.frontiersin.org/journals/public-health/articles/10.3389/fpubh.2023.1222203/full#B5)).

**Our findings also point out that patient visits by Latino and non-Latino Black, Asian, or white patients with private insurance had the highest share of telehealth visits when compared with patients with Medicaid or Medicare and those uninsured. Collectively, Asian patients with Medicaid coverage had the lowest percentage of telehealth use compared with Latino, Black, or white patients.** Other studies have found that **Asian patients adapted well to technology and have higher rates of video visits compared with Black and Latino patients** ([31](https://www.frontiersin.org/journals/public-health/articles/10.3389/fpubh.2023.1222203/full#B31)). One study found tha**t East and Southeast Asian patients specifically had overall lower telehealth utilization rates compared with non-Latino whites** ([32](https://www.frontiersin.org/journals/public-health/articles/10.3389/fpubh.2023.1222203/full#B32)). Additionally, uninsured individuals (compared with those with private insurance) and those with limited broadband coverage engaged less with telehealth during the pandemic ([32](https://www.frontiersin.org/journals/public-health/articles/10.3389/fpubh.2023.1222203/full#B32)).

Our findings suggest that telehealth access barriers exist for uninsured Latino, Black, and white patients and Asian and Pacific Islander patients with Medicaid coverage. Factors that can account for these disparities could be discomfort or lack of familiarity with telehealth, digital literacy, limited English proficiency, access to devices and broadband, and barriers to healthcare access that are a result of income or low socioeconomic status ([12](https://www.frontiersin.org/journals/public-health/articles/10.3389/fpubh.2023.1222203/full#B12), [33](https://www.frontiersin.org/journals/public-health/articles/10.3389/fpubh.2023.1222203/full#B33)–[35](https://www.frontiersin.org/journals/public-health/articles/10.3389/fpubh.2023.1222203/full#B35)). **While previous research on this topic has been conducted within safety net clinics, we also found differences in access to telehealth services based on race/ethnicity and the type of health coverage in a commercial healthcare system**. T**his study also shows that even though patients are receiving care from a well-resourced and integrated healthcare system, race/ethnicity disparities in telehealth use for the continuity of primary care are present.**

Immediately following California's SAHO, telehealth became a prominent mode of delivery of primary care services for patients living with chronic conditions. Future telehealth state and federal legislation can address gaps in telehealth use by race/ethnicity and by health coverage type. The expansion of telehealth made it possible for millions of adults with chronic conditions to have continuity of healthcare during a pandemic. Recently, Congress expanded funding and telehealth flexibilities, and permanent changes were made through 31 December 2024, continuing to allow Medicare patients to use telehealth services without geographic limitations ([2](https://www.frontiersin.org/journals/public-health/articles/10.3389/fpubh.2023.1222203/full#B2)). Future research should investigate the evolution of telehealth use by race/ethnicity and health insurance coverage after more in-person activities resumed starting in 2021 to inform the future funding and service model of telehealth services.

Our study shows that telehealth visits increased after California's stay-at-home order was issued. However, the likelihood of telehealth utilization decreased over time by the end of 2020. Our findings also highlight the gaps in telehealth use among Latino, Black, and Asian patients compared with white patients. Our results from evaluating differences in health coverage show that patients with Medicaid or Medicare, and those uninsured, consistently showed lower telehealth use compared with patients with private health insurance coverage. We also confirmed that non-English speakers were less likely to conduct visits *via* telehealth. However, language was not an additional barrier to the effect of race/ethnicity and vice versa.

Future research should investigate how to increase telehealth access to underrepresented minority and underserved patient populations, particularly among those who are uninsured or have limited health coverage. Future research should also determine barriers associated with telehealth visits, especially for patients with Limited English Proficiency (LEP) and with multiple comorbidities, an**d evaluate the impact of shifting to telehealth on continuity of care, especially among individuals experiencing a cultural-linguistic divide and/or challenges with technology literacy (i.e., older adults; patients requiring American Sign Language interpreters)**.

Study 2. Chen et al. 2021

Link: <https://www.ncbi.nlm.nih.gov/pmc/articles/PMC8415734/>

**Purpose:** To identify disparities in the use of telemedicine during the coronavirus disease 2019 (COVID-19) pandemic.

**Design:** A cross-sectional study of completed clinical encounters in an academic ophthalmology center from March 2020 through August 2020.

**Participants**: A total of 5023 patients comprising 8116 ophthalmic clinical encounters.

**Methods:** Medical charts were abstracted for demographic information. We identified zip code-level socioeconomic characteristics, which were drawn from the 2019 American Community Survey 5-year estimates.

**Main Outcome Measures:** The completion of a synchronous video encounter, the completion of a telephone (audio-only) encounter in the absence of any video encounters, or the completion of in-person encounters only.

**Results**: During the study period, 8116 total clinical encounters were completed for 5023 unique patients. Of these patients, 446 (8.9%) participated in a video encounter, 642 (12.8%) completed a telephone encounter, and 3935 (78.3%) attended clinical appointments in person only. In adjusted analysis, patients who were Black (odds ratio [OR], 0.65; 95% confidence interval [CI], 0.52e0.80; P < 0.001) or Hispanic/Latino (OR, 0.65; 95% CI, 0.49e0.85; P 1⁄4 0.002) were significantly less likely to complete a video or telephone appointment. Older patients (OR, 0.99; 95% CI, 0.98e0.99; P < 0.001), patients whose primary language was not English (OR, 0.49; 95% CI, 0.28e0.82; P 1⁄4 0.01), Black patients (OR, 0.45; 95% CI, 0.32e0.62; P < 0.001), and Hispanic/Latino patients (OR, 0.56; 95% CI, 0.37e0.83; P 1⁄4 0.005) were significantly less likely to complete a video encounter. Finally, among patients completing any type of telemedicine encounter, older age, (OR, 1.02; 95% CI, 1.01e1.03; P < 0.001), Medicare insurance (OR, 1.55; 95% CI, 1.11e2.17; P 1⁄4 0.01), and Black race (OR, 1.97; 95% CI, 1.33e2.94; P < 0.001) were associated with using only phone visits.

**Conclusions**: Ethnic/racial minorities, older patients, and non-English-speaking individuals were significantly less likely to complete a video telehealth encounter. With the expansion of telemedicine and the need to reduce the disparate impact of COVID-19 on minorities, it will be increasingly important to identify barriers to telehealth use and opportunities to improve access. Ophthalmology 2022;129:15-25 a 2021 by the American Academy of Ophthalmology

**Discussion**

**Our study found that in an urban tertiary care center, ethnic and racial minorities as well as older patients were significantly less likely to use synchronous video visits for Historically, synchronous methods of telehealth, such as video-based encounters, have been used sparingly in ophthalmology**.16 However, the COVID-19 pandemic has spurred significant growth in ophthalmologists’ capacity to provide virtual care.2 , 4 In our study, telehealth encounters constituted more than half of all visits in an entire month after the American Academy of Ophthalmology’s recommendation to reduce in-person visit frequency on **March 18, 2020**, and a statewide stay-at-home order issued on **March 20, 2020.**17 Although increased knowledge of COVID-19 transmission prevention facilitated a return to primarily in-person clinical encounters, telemedicine continued to represent a significant proportion of ophthalmic visits, particularly for patients seeing oculoplastic subspecialists and comprehensive ophthalmologists. However, the long-term role of telemedicine in ophthalmology is uncertain. Numerous studies during the pandemic have demonstrated patients’ interest in this approach and ophthalmologists’ ability to incorporate telehealth in clinical encounters for which examination and imaging are not necessary. Even before the pandemic, research indicated patient’s willingness to use telehealth for eye care: a study of glaucoma patients at a single institution found that nearly half of those surveyed expressed favorable attitudes toward using telemedicine.18 With Medicare payment for services delivered via video telehealth extended until at least the end of 2021, the establishment of telemedicine infrastructure among clinical practices, and trends in patient preference for remote care, telemedicine is likely to become a permanent method of care delivery for many patients seeking ophthalmic care.

However, continued expansion of telehealth for routine care requires careful scrutiny regarding accessibility. The COVID-19 pandemic already has been demonstrated to impact the health and financial status of vulnerable communities disproportionately, and several recent studies have identified socioeconomic disparities in telehealth use in other medical specialties.5, 6, 7 , 9 , 20 Our findings mirror many of these reports and show that older age was associated significantly with a lower likelihood of completing a video-based appointment but not a phone-based visit. *This is unsurprising, given that telephone visits are more easily accessible for patients inexperienced with technology and that increasing age is associated with lower internet access via broadband or cellular network, as well as lower ownership of digital devices necessary for video interfacing, including laptops and smart phones*.21 , 22 In contrast, greater parity exists in the ownership rates of any type of telephone among age groups in the United States.23 Technological literacy is another limiting factor for elderly patients. In a recent study of adults older than 65 years in the United States, technological inexperience was reported more frequently as the reason for unreadiness to use telemedicine, rather than infrastructural limitations.24 In a recent survey of patients with glaucoma, those older than 70 years had significantly lesser knowledge about all types of telemedicine compared with younger patients.18 Generational differences in attitudes about healthcare technology, including trust of diagnoses provided in a virtual setting or concerns about privacy, also may impact uptake among older patients.25, 26, 27 Finally, elderly patients are more likely to *have motor or sensory limitations impacting telemedicine accessibility. Visual impairment is of particular concern in patients seeking eye care, and telephone calls are likely far easier to use for these patients than video encounters.*

Our study also found that Black and Hispanic or Latino patients showed significantly lower uptake of both video as well as audio-only telehealth, a disparity that has been described in reports before and during the pandemic.8 , 9 , 13 Similar to elderly individuals, **ethnic minorities have slightly lower rates of smartphone ownership (82% among Whites, 80% among Blacks, and 79% among Hispanics), but significantly lower rates of broadband access (79% among Whites, 66% among Blacks, and 61% among Hispanics) in the United States.**11 , 12 , 21 These differences reflect wealth disparities among racial groups in the United States, and the **additional financial strain imposed by the COVID-19 pandemic may exacerbate further disparate technology access**. Because of the importance of **in-person examinations in ophthalmic care, it is likely that telehealth is used more frequently for follow-up visits.** However, **both Hispanic and Black patients are less likely to use outpatient ophthalmology services than non-Hispanic** White patients.14 Therefore, ethnic and racial minorities may have been more likely to require new, rather than follow-up, appointments during the pandemic, which are less able to be conducted in telemedicine settings. **Telehealth underuse among minorities also may be related to awareness and the advertising of such services**. A study of qualitative interviews in an underserved Hispanic population before the COVID-19 pandemic revealed **that more than 90% of participants had not heard of telehealth**.28

We also identified that patients for whom English was not their primary language showed lower use of synchronous video encounters even after controlling for other demographic and socioeconomic markers. Difficulties in accessing health care and underuse of clinical services in the United States among non–English-speaking patients is well documented, and it is unsurprising that language barriers persist in virtual settings.29 , 30 **Difficulties for these patients can include the lack of multilingual web applications and inconsistent availability of translation services from setup to appointment completion.** We did not find that the association between telemedicine use and ethnicity or race or primary language differed by the type of ophthalmology subspecialist seen. These results suggest that minority patient populations are significantly less likely to use telehealth regardless of the type of ophthalmic care they are seeking and that barriers to use are not subspecialty-specific

Differences in rates of telehealth access among patient populations were not as prevalent when including the completion of either a video or telephone visit. Indeed, on univariate analysis, older age was associated with use of any type of telehealth, which likely is reflective of the more limited technological requirements of audio-only encounters. *However, expanded reimbursement for audio-only calls are likely to be discontinued at the end of the COVID-19 public health emergency.31 This has important implications for patients who may prefer or only have access to telephone-based clinical encounters*. Our study specifically found that older patients**, Black patients,** those with Medicare, and patients seeing neuro-ophthalmologists were **significantly more likely to have completed only phone visits after controlling for demographic and SES factors**. Furthermore, in univariate analysis, **lower household income and educational status were associated with phone, rather than video, use**. Although research has demonstrated that video conferencing facilitates a higher quality of care,32 **discontinued reimbursements for phone-based visits may limit the accessibility of virtual care for these patients**. Continued expansion of telemedicine services will require simultaneous research **efforts to determine if these patient populations prefer phone visits or have limited literacy and technology to use video methods**. In the meantime, policies affect phone-based reimbursements, and policymakers should remain cognizant of potential gaps in patient access.

Differences in the use of telehealth identified in this study suggest that certain patient populations also may have been unable to access any ophthalmic care during the COVID-19 pandemic, particularly during the early phase of the pandemic when in-person encounters were severely limited. Future research specifically should examine appointment cancellations and should identify whether vulnerable populations demonstrated *lower rates appointment rescheduling, as in-person appointments were resumed.* These studies will help to identify patients who may have been lost to care and subsequently are at higher risk of vision loss.

Efforts to address existing barriers to telehealth uptake among vulnerable populations require an approach cognizant of **systemic racism and discrimination in medicine and should include strategies to improve patient trust, telemedicine literacy, and technology accessibility.** Among elderly and certain ethnic and racial minority patients, **targeted promotion and education about telehealth services via local networks (e.g., churches, grocery stores, barber shops), bilingual mediums, and nonelectronic mediums such as newspapers could increase awareness and knowledge of telemedicine**.33 **Establishment of regulatory bodies or federal policies to enhance patient privacy in virtual mediums, particularly for consumer-facing apps and devices**, is important to increase trust among populations that historically have mistrusted the health care system.34 Beyond establishing patient acceptance of telehealth, efforts should be made to increase **device access among socioeconomically disadvantaged populations.** Reduction of this digital divide will require **subsidies for internet access, which currently is being explored by the federal government in their recent proposal to increase broadband affordability for rural and ethnic minority populations**.35 Telehealth accessibility also might be improved by **health plan coverage of telecommunication devices with video capacity because of medical necessity.** Facilitating the introduction of telehealth to these patients through these changes may result in significant long-term uptake. Indeed, studies have found that patient satisfaction among elderly patients and ethnic and racial minorities after completing telehealth visits is extremely high and that after a telemedicine encounter, the majority of patients are willing to use telehealth again in the future.

Study 3. Chen et al. 2023

Link: <https://pubmed.ncbi.nlm.nih.gov/37308801/>

Understanding patient characteristics associated with scheduling and completing telehealth visits can identify potential biases or latent preferences related to telehealth usage. We describe patient characteristics associated with being scheduled for and completing audio and video visits. We used data from patients at 17 adult primary care departments in a large, urban public healthcare system from August 1, 2020 to July 31, 2021. We used hierarchical multivariable logistic regression to generate adjusted odds ratios (aOR) for patient characteristics associated with having been scheduled for and completed telehealth (vs in-person) visits and for video (vs audio) scheduling and completion during two time periods: a telehealth transition period (N = 190,949) and a telehealth elective period (N = 181,808). Patient characteristics were significantly associated with scheduling and completion of telehealth visits. Many associations were similar across time periods, but others changed over time. Patients who were older (≥ 65 years old vs 18–44 years old: aOR for scheduling 0.53/completion 0.48), Black (0.86/0.71), Hispanic (0.76/0.62), or had Medicaid (0.93/0.84) were among those less likely to be scheduled for or complete video (vs audio) visits. Patients with activated patient portals (1.97/3.34) or more visits (≥ 3 scheduled visits vs 1 visit: 2.40/1.52) were more likely to be scheduled for or complete video visits. Variation in scheduling/completion explained by patient characteristics was 7.2%/7.5%, clustering by provider 37.2%/34.9%, and clustering by facility 43.1%/37.4%. Stable and dynamic associations suggest persistent gaps in access and evolving preferences/biases. Variation explained by patient characteristics was relatively low compared with that explained by provider and facility clustering.

**Discussion**

In this observational study of scheduled and completed primary care visits at a large, urban, public healthcare system, we found that there were modest differences between demographic groups in patterns of telehealth scheduling and completion and variation in these differences over time. However, while there were some differences in telehealth usage based on patient characteristics, far more variation could be explained by clustering at provider or facility levels, with patient-level variation contributing the least.

Demographic differences were present in scheduled visits, suggesting potential latent preferences by patients, clinicians, or facilities or biases in offering telehealth modalities. In the telehealth transition period, patients who were older than 65 years old, **Black, Hispanic, or Other Race, or who preferred Other Languages were** l**ess likely to have telehealth visits scheduled relative to patients in their comparator groups**. In the telehealth elective period (compared with the telehealth transition period), some differences became more pronounced; for example, patients older than 65 years old were even less likely relative to younger patients to scheduled telehealth visits. New differences also emerged; for example, during the telehealth transition period, patients at all levels of comorbidity had similar propensities to schedule telehealth visits, but in the elective telehealth period, **patients with two or more comorbidities were less likely than those with no comorbidities to schedule telehealth visit**s. Overall, this **suggests that there is selection happening at the time of scheduling that may influence demographic differences in telehealth utilization and that such selection may change over time.**

**Our findings show decreased propensity to complete telehealth visits and to participate in video telehealth for older, Black, Hispanic, other race, non-English speaking, and uninsured patients and higher propensity for patients who were female, Asian, had an activated patient portal, more comorbidities, and more scheduled visits**. Prior literature on demographic factors associated with telehealth usage has generally been mixed, with some factors being positively associated in some studies but negatively associated in others [2-10]. While the relevant factors remain relatively constant, this variation hints that there ma**y be contextual elements that may be more influential than demographic block identifiers.**

In both time periods, the factor most positively associated with audio or video telehealth scheduling and completion was the number of scheduled visits during the period of study. To our knowledge, this study is among the first to report on this association [11]. This suggests that patients can, **over time and with exposure, learn to become telehealth users (and video users) despite any differences related to demographic variation**. Additionally, it suggests that for patients who have multiple visits over the course of a year, it may make sense for both the provider and patient for some of those visits to be done using telehealth.

Consistent with a study of a large, integrated health system that reported proportions of observed variation in telehealth completion explained by patient factors versus clinician- **or facility-level clustering [3], we saw that a majority of observed variation is due to clinician and facility clustering**. While much discussion has been centered around disparities or inequities in care delivery driven by patient demographic factors or proxies for digital access and literacy, additional focus is needed to examine and address gaps **in clinician comfort with telehealth, workflows/practices, and potential biases therein**. These issues may partly relate to **design of telehealth platforms, and presence or absence of critical functions for diverse patient, clinician, and staff user populations such as interpreter services integration and a simple user experience. Beyond this, more clinician and staff training and usage of structured assessments for patients’ telehealth capabilities or preferences may help minimize variation in practice due to biases from within the health system.**

Study 4. Chumbler et al. 2022

Link: <https://pubmed.ncbi.nlm.nih.gov/36895424/>

Abstract

**Introduction:** The COVID-19 pandemic led to a major transition for patients from routine ambulatory-care-based in-person primary care visits to telehealth visits to manage chronic diseases. However, it remains unclear the extent to which individuals access telehealth services and whether such utilization varies along neighborhood characteristics, especially among racial minorities. This study aims to examine the association of outpatient telehealth utilization with sociodemographic, clinical, and neighborhood characteristics among adults with ambulatory care sensitive conditions (ACSCs) during the COVID-19 pandemic.

**Methods**: We included adults treated for an ACSC between March 5, 2020, and December 31, 2020, at a single ambulatory-care-based healthcare system, which serves a large population of low-income patients in the South region of the United States (i.e., Memphis, TN, Metropolitan Statistical Area). Telehealth utilization was defined by outpatient procedural codes and providers' notes on the type of visits. Generalized linear mixed models were used to examine the association of sociodemographic, clinical, and neighborhood factors with telehealth utilization in the overall cohort and the racial subpopulations.

**Results:** Among the 13,962 adults with ACSCs, 8583 (62.5%) used outpatient telehealth services. Patients who were older, female, with mental disorders, and who had more comorbidities h9ad higher rates of telehealth services (p < .05). Controlling for covariates, we observed 75.2% and 23.1% increased use of telehealth services among Hispanics and other race groups, respectively, compared to Whites. Patients who commuted more than 30 minutes to health facilities were slightly less likely to use telehealth services [OR: 0.994 (0.991,0.998)]. **Racial minorities (Blacks and Hispanics) with mental disorders were more likely to use telehealth service when compared to Whites.**

**Discussion:**

It is well known that the COVID-19 pandemic not only substantially increased the use of telehealth services, but also caused an extraordinary situation for healthcare.9,25 COVID-19 disproportionately affected individuals with multiple physical comorbidities, obesity, advanced age, and ethnic minorities,9 all of which represent clinical and sociodemographic characteristics that were found to be associated with worse COVID-19 health outcomes and have been found to be associated with an increased use of health services. Even though telehealth services significantly increased since the inception of COVID-19, it was unclear the extent to which clinical and sociodemographic factors were associated with individuals’ access of telehealth services, especially those with multiple comorbidities and especially among racial subpopulations. In fact, the few studies have reported inconclusive findings. For instance, prior to the COVID-19 pandemic, studies tended to report that Hispanic and Black patients were less likely to use telehealth services as compared to White patients, whereas other studies reported that there were no racial and ethnic disparities in telehealth service use.26–28

Systematic racism could have been associated with racial and ethnic minorities being more likely to suffer from COVID-19 and therefore being at greatest need for telehealth services.29,30 Thus, telehealth services could be extremely beneficial for people of color who had poor health outcomes from COVID-19 to not only to receive medical advice about symptoms related to the virus, but also to manage chronic physical comorbidities because they are more likely to have such conditions (e.g., diabetes) that not only require ongoing medical care, but may lead them to telehealth services.29,31 **However, compounded with this challenge is that people of color with both access to and comfort with technology is lower than White individuals**.32 Most of the previous studies have failed to consider community and neighborhood characteristics as covariates. These socioeconomic and community level factors are key variables of social determinants of health and can serve as key covariates in the generalized linear models. **To fill an important void in the literature, we employed variables that gauge levels of poverty, percent of population who had at least a college education, and a key indicator of access to care (i.e., percent of population who commute more than 30 minutes to the nearest health facility).**

During the first 9 months of the COVID-19 pandemic, we found that 62.5% patients with ACSCs utilized outpatient telehealth services. Our findings underscored several disparities in telehealth service utilization. We found differences by gender, age, race and ethnicity, presence of mental disorders, comorbidity, as well as drive time from patients’ place of residence to the closest health facility. **More specifically, GLMMs found that patients who were Hispanic and from other race groups (not including Black patients) were significantly more likely to use telehealth services as compared to White patients,** findings that were consistent with a recent secondary analysis of self-report data from national, probability-based online panel of adults (18 or older) living in US households.29 That is, Campos-Castillo and Anthony (2020) found that as compared to White respondents, H**ispanic and those from other race groups (excluding Black respondents) reported using telehealth services more often**. This finding from our study was also congruent with a retrospective cohort study of older members of Kaiser Permanente Southern California system. **Qian and colleagues (2021), found that those in the Hispanic group had the largest increase in telehealth utilization in response to the COVID-19 pandemic**.33 However, once multivariate analyses were applied to their dataset, only Black respondents were more likely than Whites to report using telehealth services.29 Another study from a non-profit healthcare system in seven states in the West and Southwest of the US, found that compared to White patien**ts, Hispanic/Latino patients were significantly less likely to use telehealth services before and after COVID-19 diagnosis**.34

Other recent research found that differences between Black and non-Black patients in terms of primary care appointment completion rates narrowed significantly due to the increased use of telehealth services.35 However, in contrast to these findings, Chunara and colleagues (2021), who conducted a study from a large academic healthcare system in New York City, found that after controlling for individual and **community level attributes of telehealth patients, the Black patients were significantly less likely to access care through telemedicine compared to White patients**.36 Similarly, Friedman et al. (2022) from an urban tertiary hospital in the Midwest, found that Black race and participants of other races were less likely to use telehealth compared to Whites.37 Thus, **the current state of the literature has found mixed results on racial and ethnic differences in telehealth service utilization.**

Even though our multivariate analyses found that Black patients were comparable to white patients in terms of telehealth utilization, on the other hand, we found that two specific racial minorities (Blacks and Hispanics) with mental disorders were more likely to use telehealth services than their white counterparts. To our knowledge, there have been no previous studies that have specifically examined how the interplay between mental illness and racial minorities is associated with increased telehealth use. However, recent research did report racial/ethnic disparities in outpatient visits for mental health and/or substance use disorders during the COVID surge and partial re-opening in Massachusetts. More specifically Yang (2020) found that Hispanics and non-Hispanic Blacks reported decreases in visits for mental health and/or substance use disorders.38 Future research should further investigate these relationships, since telehealth services play an important role in access to mental health services, especially in terms of racial minorities.39 **This need is particularly important because there is a “digital divide” with reference to access to computer and home broadband availability for Black and Hispanic adults**.38,40 In fact during the pandemic, Black and Southern residents were less likely to use telehealth due to lack **of broadband access.41**

Consistent with studies both prior to and during the COVID-19 pandemic,25 we found that in both the main and subgroup analyses, advanced age was correlated with increased telehealth service use. This is consistent with a longstanding body of research that has found that telehealth services offer an important role in managing symptoms of chronic health conditions for older individuals.42 In both the main and subgroup analyses, higher CCI scores was associated with increased telehealth utilization rates, with Black patients having higher rates than their White counterparts. A previous study found that Black citizens tend to have higher rates of disabling chronic health conditions (e.g., diabetes) than their White counterparts.43 **Thus, our findings were encouraging that Black patients who had more comorbidities had greater odds of using telehealth services.**

The study has some limitations. First, EMR data were used from practices serving predominantly African American populations in the Memphis MSA, and the results may not be generalizable to larger racially diverse populations. Second, it is an observational study, and the analyses could only identify the association of demographic, clinical, and neighborhood-level factors with the outpatient telehealth use and not causality. Third, this study assesses the telehealth service utilization rates during the first year of the COVID-19 pandemic. A longer study period would be essential to examine changes in the rates of outpatient telehealth use after the onset of COVID-19 pandemic. Fourth, our study was limited to low-income zip codes that surrounded the Memphis MSA. Therefore, it is unclear how our findings can be generalizable to other regions, especially those areas with residents who have more income or who reside in suburban and/or rural areas.

Study 5. Chunara et al. 2021

Link: <https://www.ncbi.nlm.nih.gov/pmc/articles/PMC7499631/>

Abstract

**Objective:** Through the coronavirus disease 2019 (COVID-19) pandemic, telemedicine became a necessary entry point into the process of diagnosis, triage, and treatment. Racial and ethnic disparities in healthcare have been well documented in COVID-19 with respect to risk of infection and in-hospital outcomes once admitted, and here we assess disparities in those who access healthcare via telemedicine for COVID-19.

**Materials and Methods:** Electronic health record data of patients at New York University Langone Health between March 19th and April 30, 2020 were used to conduct descriptive and multilevel regression analyses with respect to visit type (telemedicine or in-person), suspected COVID diagnosis, and COVID test results.

**Results:** Controlling for individual and community-level attributes, Black patients had 0.6 times the adjusted odds (95% CI: 0.58–0.63) of accessing care through telemedicine compared to white patients, though they are increasingly accessing telemedicine for urgent care, driven by a younger and female population. COVID diagnoses were significantly more likely for Black versus white telemedicine patients.

**Conclusion:** Telemedicine access disparities reflect those in in-person healthcare access. Roots of disparate use are complex and reflect **individual, community, and structural factors, including their intersection—many of which are due to systemic racism.** Evidence regarding disparities that manifest through telemedicine can be used to inform tool design and systemic efforts to promote digital health equity.

**Discussion:** In this prospective cohort study, which included 140 184 patients who sought care at NYULH during the acute pandemic period (between March 19, 2020 and April 30, 2020), we found that the proportion of Black patients accessing care through telemedicine increased compared to the same time period in 2019. Supplementary analysis showed that this change was driven primarily by a younger and more female population. However, after controlling for individual and community-level attributes of telemedicine patients, Black patients were significantly less likely to access care through telemedicine compared to white patients in 2020, aOR 0.60 (95% CI, 0.58–0.63). These adjusted odds of Black patients using telemedicine as compared to in-person office or ED visits in comparison to white patients was also lower than that of other race/ethnicity groups (Table 3). Having accessed care, the odds of a suspected COVID diagnosis after a visit was higher for Black patients who access care through telemedicine compared to white patients, aOR 1.07 (95% CI, 1.02–1.13), and lower compared to white patients when considering in-person and telemedicine visits, aOR 0.91 (95% CI, 0.87–0.95). Further, of the patients who accessed care through telemedicine, and were subsequently tested at NYULH, the odds of a Black patient testing positive for the virus (of those who got tested) were increased compared to white patients, aOR 1.63 (95% CI, 1.36–1.94). Considered together, these findings suggest that, while Black patients accessed telemedicine at higher numbers during the acute pandemic period than prior, they are utilizing at lower levels when compared to white patients and may be sicker when seeking care through telemedicine compared to white patients. We conclude this because Black patients are more likely to receive a suspected COVID diagnosis after a visit, and also to test positively if tested. It could also be that white patients are more likely to get tested, which could be because of easier access or due to access based on concern, information seeking, or a milder illness that may or may not be COVID. Finally, it should be noted that in supplementary analyses, we observed that the proportion of patients seeking urgent care through telemedicine from 2019 to 2020 who are Black increased by 4.6% (95% CI, 2.6% to 6.7%), while the percent seeking care through the ED increased by 2.0% (95% CI, 1.2% to 2.8%) showing increased use of telemedicine for urgent care by Black populations

**Comparison to existing literature**

Racial and ethnic disparities in healthcare have been well-documented in COVID-19, specifically with respect to risk of infection and in-hospital outcomes once admitted.4,5 While recent research has found significant disparities in COVID-19-related healthcare among specific populations—including Black, Latinx, migrant workers, and elderly—much of the focus on this work has been on outcomes, access, and equity in in-person care. As yet, we have not seen any studies empirically demonstrating how the disparities faced by these populations have been impacted by virtual care services such as telemedicine during the pandemic. Overall, our study adds to the literature through an investigation of disparities, specifically related to healthcare access through telemedicine use, during a period when the impetus for remote healthcare access was high.

Our institution is uniquely suited to explore the impact of race, racism, and digital **disparities on telemedicine given its well-developed digital health infrastructure, including digital patient platforms, high levels of patient portal (MyChart) participation, and robust telemedicine program.** These programs were heavily **invested in and socialized to patients during both the prepandemic and acute pandemic period**.26 As a result of this investment, the potential for confounding of telemedicine technical capacities (**such as technical glitches, compatibility/interoperability challenges, and provider technical competence) from an institutional perspective was likely minimized**.15 Our work suggests that disparate access to and utilization of digital healthcare technologies is driven, at least in part, **by features and processes beyond the digital infrastructure limitations of healthcare systems (such as the availability of telemedicine-capable clinicians), and further suggests that the mere availability of digital health services may not be enough to address disparities in digital access and utilization.** Similarly, the high rates of insured patients in our population (as compared to city averages) suggest that the disparities we observe are **unlikely to be linked solely to insurance status or coverage type.** This conclusion is supported by findings of disparities in COVID-related infections and deaths across several metropolitan areas, although the study only considered community-level factors.27

Moreover, the observed intersectionality of disparities,14 that is, in Black and older patients, echoes well-documented challengesin healthcare due to institutional sexism and ageism (**due to bias and cultural competencies among providers, access to resources, and other reasons**);13,28 and again our study highlights that these forces manifest in healthcare access via telemedicine. In the next section, we discuss how this mechanism behind disparities (racism, ageism) can be addressed specifically for telemedicine systems.

At the community level, our findings, regarding **median income being associated with decreased odds and median household size with positive odds of positive COVID tests from our cohort which accessed care through telemedicine**, echo findings from other COVID studies that aren’t limited to patients who access care through telemedicine.19 Further, we found that **mean income and median household size of the home zip code had decreased odds for use of telemedicine versus seeking care in-person;** evidence from patient surveys and other research should be used to investigate reasons for these associations.

**How this study informs design of telemedicine systems**

**Identifying where in the healthcare process disparities manifest is essential in order to inform effective programs, empower patients, and improve health outcomes. While systematic racism and ageism are well-documented in healthcare and healthcare access, this study gives evidence that such processes may also occur in telemedicine use.** Here we describe how the design of telemedicine systems can be improved to decrease barriers to diverse populations.29

Findings from this and other digital disparities work can inform the design of telemedicine systems through the development of **culturally and structurally appropriate tools and technology, representative provider presence and capacity, positive targeted outreach, and research.** In developing tools and technology, efforts should be made to incorporate knowledge of the myriad factors affecting healthcare access and utilization at patient- and community-level, **including knowledge, attitudes, cultural beliefs, health behaviors, adherence, language, health literacy, social support, religious beliefs, self-efficacy, preferences, and psychosocial factors**. This can be achieved by drawing on methods that leverage participatory design; for example, it is known that information technology interfaces that include **cultural and linguistic adaptations can aid in helping patients manage online systems, and prior studies have included patient and community participants to culturally tailor online systems.**9,30–33 **Building provider representativeness and capacity in telemedicine can draw from abundant existing data showing that Black, Latinx, and Native American physicians are more likely than white physicians to practice in underserved communities and to treat larger numbers of minority patients irrespective of income**,34 and that Black and Latinx physicians, as well as women, are more likely to provide care to the poor and under- and uninsured.35,36 It is reasonable to suggest that ensuring similar **representativeness among telemedicine providers would improve the telemedicine experience for diverse patients, reduce disparities, and improve health outcomes.** Positive targeted outreach should ensure that communities identified as experiencing barriers to care via telemedicine **are actively contacted, connected, empowered, and protected.** For example, it has been shown that the **intentional dissemination of health information to older Black adults can increase their utilization of preventive health services.**37 Accordingly, **communication of information on the use and availability of telemedicine could be studied in terms of how it may appeal to diverse populations in trustworthy ways.** Another possible solution includes identifying which specific subgroups have the most difficulty accessing care **through telemedicine and using this information to prioritize in-person care in situations (such as pandemics) when in-person interactions should otherwise be limited**. Other systematic approaches to lowering barriers for diverse populations include: **the continuing collection of data on disparities and publication of results**; **the systematic identification of biases and the development of educational and training programs to address them; and removing as much individual discretion of providers as possible (eg, specifically during this period, standardized and ready testing availability would help**).38

Suggestions for future work

Given findings of decreased odds of seeking care via telemedicine, but increased odds of testing positive for Black patients compared to white patients at NYULH (controlling for individual-and community-level attributes), it appears that Black patients are accessing care through telemedicine when they are sicker compared to white patients. As well, patients with preferred language of Spanish had lower odds of telemedicine use. These findings reflect in-person care-seeking behavior that has been observed at other institutions during COVID-19,3 and indicate that factors related to in-person care access disparities such as **trust in providers, health literacy, and language barriers could be relevant in telemedicine as well.** Efforts to mitigate these challenges may be different in telemedicine versus in-person care (eg, the use of a virtual instead of in-person translator), thus research on specific factors related to decreased care-seeking through telemedicine are needed to best mitigate these challenges. Research on subgroup-specific challenges is also needed, given our observation that telemedicine access disparities are most prominent for intersectional groups (the larger increase in the proportion of patients who are Black and sought care from 2019 to 2020 through virtual urgent care compared to in-person in the ED, was driven by a younger and more female population). The finding that, controlling for patient- and community-level attributes, Black patients were more likely to be given a diagnosis of suspected COVID after a telemedicine visit compared to those who are white, while less likely when considering both telemedicine and in-person visits, should also be investigated further. For instance, the reason for this could be that the online platform normalizes interactions between the clinician and patient through the virtual medium39 and decrease healthcare diagnosis biases, offering a possible mitigating role for telemedicine with respect to healthcare disparities.40

Study 6. Cousins et al. 2022

Link: <https://pubmed.ncbi.nlm.nih.gov/36395112/>

Abstract

**Introduction:** The COVID-19 pandemic drove rapid adoption of telehealth across oncologic specialties. This revealed barriers to telehealth access and telehealth-related disparities. We explored disparities in telehealth access in patients with cancer accessing oncologic care.

**Materials/methods:** Data for all unique patient visits at a large academic medical center were acquired pre- and intra-pandemic (7/1/2019-12/31/2020), including visit type (in-person, video, audio only), age, race, ethnicity, rural/urban (per zip code by Federal Office of Rural Health Policy), distance from medical facility, insurance, and Digital Divide Index (DDI; incorporates technology/internet access, age, disability, and educational attainment metrics by geographic area). Pandemic phases were identified based on visit dynamics. Multivariable logistic regression models were used to examine associations of these variables with successful video visit completion.

**Results:** Data were available for 2,398,633 visits for 516,428 patients across all specialties. Among these, there were 253,880 visits from 62,172 patients seen in any oncology clinic. Dramatic increases in telehealth usage were seen during the pandemic (after 3/16/2020). In multivariable analyses, patient age [OR: 0.964, (95% CI 0.961, 0.966) P<0.0001], rural zip code [OR: 0.814 (95% CI 0.733, 0.904) P = 0.0001], Medicaid enrollment [OR: 0.464 (95% CI 0.410, 0.525) P<0.0001], Medicare enrollment [OR: 0.822 (95% CI 0.761, 0.888) P = 0.0053], higher DDI [OR: 0.903 (95% CI 0.877, 0.930) P<0.0001], distance from the facility [OR: 1.028 (95% CI 1.021, 1.035) P<0.0001], black race [OR: 0.663 (95% CI 0.584, 0.753) P<0.0001], and Asian race [OR: 1.229 (95% CI 1.022, 1.479) P<0.0001] were associated with video visit completion early in the pandemic. Factors related to video visit completion later in the pandemic and within sub-specialties of oncology were also explored.

**Conclusions:** **Patients from older age groups, those with minority backgrounds, and individuals from areas with less access to technology (high DDI) as well as those with Medicare or Medicaid insurance were less likely to use video visits.** With greater experience through the pandemic, disparities were not mitigated. Further efforts are required to optimize telehealth to benefit all patients and avoid increasing disparities in care delivery.

Digital divide index (DDI)

**Discussion**

Through analysis of all patient visits in a large academic medical center, we noted dramatic shifts in telehealth utilization during the early days of the COVID-19 pandemic. **Among those seeking cancer care, we found that older individuals, African Americans, those utilizing Medicaid, and those from areas with higher DDI were less likely to complete video visits**. These factors support a number of considerations for future telehealth applications across oncologic specialties. First, older individuals who make up the bulk of oncology patients may need additional help in accessing telehealth. Second, racial inequities in cancer care may be exacerbated through telehealth, suggesting the need for effective means to mitigate telehealth-related disparities in the post-COVID-19 world. Third, **those from rural areas may require additional support in order to access telehealth resources**. Fourth, Digital Divide Index is related to telehealth use, **suggesting that investments in broader access to the internet and quality education may have important positive implications for oncologic care access.** There is great interest in preserving telehealth advances to facilitate care delivery in the future. Our analyses supports the findings of others who have suggested that addressing barriers and inequality concerns will be critical to ensure full access for all oncology patients [15,18,21].

**Visit dynamics during the pandemic**

Institutional guidance borne out of efforts to protect patients and providers from COVID-19 led to initial shifts in visit types during the pandemic in the general patient population and among patients seen by oncologic specialties at our institution. Others have shown dramatic shifts in visit type in response to COVID-19 as visit dynamics have been reported extensively in the growing COVID-19 pandemic literature [22,23]. Across the US and around the world, guidance came at slightly different timepoints in the pandemic, accounting for locoregional variation in viral case numbers and resources. While generalizations across the United States with regard to the precise date selections that we have made in Fig 1 are not possible, it is likely that review of data from many institutions in the United States would demonstrate large shifts in visit composition at timepoints specific to individual healthcare systems as they responded to the needs of their patient populations. **Though large shifts occurred universally, the nature of these shifts was likely dictated by many features of individual institutions such as technological resources and telehealth expertise as well as other contextual factors beyond individual health systems**. **Generally, the timing seen at our institution in March 2020 is consistent with timing reported at other institutions** [23].

Outside of health system and institutional decisions made in the context of an understanding of the pandemic locally, there are other factors that should be considered at different levels that impact whether an individual patient might complete a video visit. These might vary across the pandemic period. **Payor practices have guided telehealth use and enabled dramatic increases in telehealth use** [24], **highlighting the fact that any approach to telehealth in oncology will require collaboration between medical institutions and payors** [25]. Additionally, patients can be taught, so **it is likely that some patients might have gained the capacity to complete telehealth visits during the pandemic. Providers and institutions might also have varied skill sets and resources to enable patients’ efforts to engage in telehealth**. **Patient and provider skills as well as payor guidance are all in flux along with the pace of the pandemic itself**. It will be important for institutions, payors, providers, and patients themselves to have a voice in the process of refining telehealth as a component of cancer care delivery. Additionally, it will be important to consider all of the many factors that impact telehealth use in future studies designed to optimize its fair and ethical application.

**Predictors of telehealth access in oncology**

**Older individuals As age increased, patients were less likely to use video visits. It is known that many older adults lack access to the technology and expertise required to participate fully in telehealt**h [26]. Younger patients with cancer are more likely to prefer telehealth visits than older patients with cancer [27]. Outside of oncology, analyses across primary and specialty practices have shown that older adults are less likely to participate in video visits [22,28]. A study of patients at another cancer center similarly found that older individuals were less likely to use video visits [29]. In a previous non-oncology focused analysis from our institution during a shorter time frame than that outlined in our manuscript, the mean ages for video visit users, phone visit users, and non-telehealth users were 42, 56, and 41, respectively [28]. However, in our analysis, the mean ages for video visit users, phone visit users, and non-telehealth users were 54, 63, and 56, respectively. Because the oncology patient population is older than the general population, any age-related burdens or barriers will negatively impact patients with cancer to a greater degree. Patients seen in radiation oncology were the oldest of the three oncologic specialties, suggesting that age-related telehealth access difficulties may be most challenging in radiation oncology, among oncologic providers. Our work agrees with the findings of others who have noted that special attention must be paid to older patients to facilitate access to telehealth [30,31].

Race People of color have been shown to have greater difficulty accessing telehealth in multiple prior studies [31–33]. **In the data described in this report, the impact of race on video visit completion varied somewhat depending on whether the analysis included all oncology patients or various subspecialties that make up oncology**. It is likely that these differences are the result of variable patient numbers and power to obtain statistical significance given the magnitude of odds ratios shown in Fig 3 (also S1 Appendix). Our findings in the aggregate analysis of all oncology visits resulted in similar findings to those of Shao et al who studied a population from an NCI designated cancer center in the state of Alabama (in a population with greater minority representation than that seen in our study population) and found that patients of color were less likely to use video visits [29]. This supports the concern that telehealth might exacerbate existing inequities in healthcare and cancer care specifically if countermeasures are not developed [19].

Gender Some have found an impact of gender on video visit completion, specifically male gender [29]. We did not see an impact of gender in our aggregate analysis of oncology visits. However, an effect of gender was noted variably across pandemic phases and specialties such that it is difficult to draw strong conclusions on the impact of gender using our data (S1 Appendix). Females were less likely to complete video visits in Phase 2 within radiation oncology and Phase 1 within surgical oncology. Further exploration of the impact of gender is warranted as efforts to optimize telehealth access proceed.

DDI The Digital Divide Index predicted video visit completion in the present study. This metric is complex in that it incorporates access to broadband internet, computing devices, download speed, upload speed, age, education, poverty rate, and disability within a geographical area. It was highly significant in all analyses in models containing age. Therefore, it is likely that the effect is driven by other components of the score. Many of the components relate to available infrastructure. Further enhancing access to broadband is likely to increase access to telehealth. Likewise, the significance of DDI may also point to the potential for telehealth to worsen educational attainment-related disparities in health outcomes in oncology. Patients from areas with lower income have been shown to be less likely to complete video visits by others [29]. Regional socioeconomic status and DDI are also clearly quite closely entwined. DDI could be easily adapted to guide interventions to address barriers to telehealth access.

Insurance In our study, those with Medicare or Medicaid were less likely than those with private insurance to engage in video visits.

This is similar to the results of an examination of patients at another cancer center, where patients with public insurance were less likely to engage in video visits [29].

Rural zip code Those from rural zip codes were less likely to complete video visits. These findings generally parallel findings of previous research on factors that are related to lower rates of patient portal usage [31]. Efforts to improve oncologic care delivery through the use of telehealth are being studied; these ongoing efforts will need to carefully account for telehealth access-related concerns of rural patients with cancer [34].

Distance Increasing distance from the medical facility was associated with increased ability to complete video visits. This was somewhat unexpected and may have been a product of a **greater perceived incentive in the form of avoiding a lengthy trip to receive care.** For example, those patients at greater distance from healthcare facilities might **be more likely to organize approaches to take part in telehealth and to plan ahead prior to virtual visits with providers in hopes of avoiding the extra cost and effort required to attend visits in person**. It is also possible that **individuals living in remote areas were more likely to have engaged in video visits with another system prior to their encounter in our system.** These previous visits might then have served as practice sessions, **allowing patients time to train themselves prior to their initial encounters with our cente**r. A better understanding of factors t**hat motivate patient engagement with telehealth will be important to the further adoption of telehealth in the future.**

**The future of telehealth in oncologic care**

Dramatic changes in oncology department operation occurred during the COVID-19 pandemic [13,35]. Great efforts were undertaken in radiation oncology practices toward reducing the number of patients under treatment [18,36]. Changes were suggested for infusion protocols in medical oncology practices [13]. Surgeons were encouraged to consider non-operative management where possible [14]. As oncologic care has reached a new steady state, physicians express high levels of satisfaction with the use of telehealth [16,37,38]. These providers report that they will continue offering telehealth visits, and practice guidelines for telehealth have been developed [15,39–41].

Despite this, support is not uniform; skeptics caution that telehealth may lead to lower quality patient care [18] or exacerbate care disparities. Additionally, patients with positive impressions of telehealth still recognized the importance of in-person physical exams for detection of cancer recurrence [42]. This dialog supports discussion of approaches to balance in-person and telehealth visits to achieve patient and provider goals. Some have envisioned ways that telehealth could help reduce disparities [17,21]. As elements of telehealth continue beyond the COVID-19 pandemic in the area of on treatment monitoring in radiation oncology, long term follow up in all specialties, and in selected pre-treatment settings [17,25,43], developing models of telehealth that balance in-person and remote patient care priorities while addressing disparities will be necessary prerequisites for quality care delivery across oncologic specialties.

Were improvements observed over the course of the pandemic?

At our institution, two distinct pandemic phases were noted. Though many have examined telehealth during the pandemic and found inequities early in the pandemic, we show that these inequities persisted beyond the initial phase of the pandemic. There is little difference in significance of many key features from Phase 1 and Phase 2. Specifically, the odds ratios for the impact of race, DDI, Medicaid enrollment status, and age are largely unchanged between Phase 1 and Phase 2. This illustrates that there is still much work to be done to improve access to telehealth-based oncologic care.

Study 7. Duan et al. 2022

Link: <https://www.ncbi.nlm.nih.gov/pmc/articles/PMC9115291/>

Abstract

**Background/Objectives:** The COVID‐19 pandemic necessitated rapid implementation of telemedicine at medical centers across the United States. As telemedicine is expected to persist beyond the pandemic in subspecialties like pediatric dermatology, there is growing concern that socioeconomic factors may contribute to inequitable telemedicine access. This study aims to identify factors associated with disparities in telemedicine use among pediatric dermatology patients during the pandemic.

**Methods:** In this single‐center cross‐sectional study, patients less than 18 years old who completed a visit with a pediatric dermatologist via a video telemedicine call or in‐person office visit during the specified time periods were included. Univariate and multivariable analyses were performed to compare demographic factors for (1) patients who had a telemedicine visit versus office visit during June 1, 2020, to January 22, 2021, and (2) patients who had either visit type during June 1, 2020, to January 22, 2021, versus June 1, 2019, to January 2020.

**Results:**The independent factors associated with lower odds of telemedicine include identifying as Black/African American, having a non‐English preferred language, and having public insurance, whereas the independent factors reducing overall access to pediatric dermatology care during the pandemic include identifying as Hispanic/Latino and having public insurance.

**Conclusions:** Differential telemedicine use in vulnerable communities may be attributed to **disparities in technology access and digital literacy** and should be addressed at a structural level. If such disparities are identified and adequately remedied, telemedicine can serve as an important tool for expanding access in the field of pediatric dermatology.

**Discussion**

Our work reveals the existence of disparities in telemedicine access during the COVID‐19 pandemic among pediatric dermatology patients at our medical center. Specifically, we found that the independent factors associated with lower rates of telemedicine use include identifying as Black/African American, having a non‐English preferred language, and having public insurance. These findings corroborate studies in other medical fields, demonstrating the prevalence of inequitable telemedicine access across specialties and patient populations. 3 , 4 , 5 , 6

Disparities in telemedicine use among patients of color **could be attributed to the intersection of individual and systemic factors**. Particularly, **structural racism may contribute to lower technology access and digital literacy in Black communities**. 13 , 14 We found that, although the odds of Black/African American patients seeking care during the pandemic **was similar to pre‐pandemic**, the odds of using telemedicine was significantly less relative to White patients. This suggests that barriers to telemedicine access disproportionately affected Black patients seeking care during the pandemic, leading to a greater reliance on in‐person visits than other groups. We hypothesize that **technology‐associated disparities including access to video‐enabled devices and digital literacy m**ay drive lower telemedicine use, 15 supported by our results that Black patients were significantly less likely to have an **activated patient portal and email addresses** on record relative to White patients. Finally, possible **mistrust of the health care system and technology in Black communities may contribute to lower rates of telemedicine use** 3 , 14 and may influence patients of color to prefer in‐person medical visits, especially if meeting with a new provider.

Next, **we found that patients who were Hispanic/Latino and non‐English speaking were disproportionately affected by the COVID‐19 pandemic**. While having a non‐English preferred language factors into likelihood of telemedicine use, identifying as Hispanic/Latino played a larger role in accessing care during the pandemic overall regardless of visit type, even after adjusting for covariates. We hypothesize that factors other than language barriers influenced the ability of Hispanic/Latino patients to seek care during the pandemic, while language barriers specifically made telemedicine challenging for this group despite the availability of medical interpretation during virtual visits. 7

Finally, patients with public insurance had significantly lower odds of using telemedicine despite widely expanded coverage for telehealth services by U.S. health insurance plans. 16 **Thus, we propose that public insurance as a proxy for income rather than insurance type alone predicts decreased telemedicine use. Telemedicine disparities among low‐income households are likely attributable to gaps in access to the technology and internet connectivity necessary to complete virtual visits**

This study has several limitations. First, the cross‐sectional design allows only for broad analysis of the defined time periods and does not show how changes may have gradually occurred. The retrospective data availability made some results difficult to interpret, such as how patients of unspecified race and ethnicity appear to have significantly higher odds of accessing care during the pandemic. One explanation could be that there were logistical challenges that reduced the completeness of documenting patients' race and ethnicity data during the pandemic. Finally, the single‐institution setting may challenge the generalizability of the findings, though an advantage to this design is the elimination of interinstitutional variability with respect to telemedicine protocols and implementation timelines.

Study 8 Ekwegh .et al. 2023

Link: <https://www.ncbi.nlm.nih.gov/pmc/articles/PMC9915549/>

Abstract

**Background**: The COVID-19 pandemic transformed healthcare delivery with the expansive use of telemedicine. However, health disparities may result from lower adoption of telehealth among African Americans. This study examined how under-resourced, older African Americans with chronic illnesses use telehealth, including related sociodemographic and COVID-19 factors.

**Methods**: Using a cross-sectional design, 150 middle-aged and older African Americans were recruited from faith-based centers from March 2021 to **August 2022.** Data collected included sociodemographics, comorbidities, technological device ownership, internet usage, and attitudes toward COVID-19 disease and vaccination. Descriptive statistics and multivariable regression models were conducted to identify factors associated with telehealth use.

**Results**: Of the 150 participants, 32% had not used telehealth since the COVID-19 pandemic, with 75% reporting no home internet access and 38% having no cellular/internet network on their mobile device. **Age, access to a cellular network on a mobile device, and wireless internet at home were significantly associated with the utilization of telehealth care. Higher anxiety and stress with an increased perceived threat of COVID-19 and positive attitudes toward COVID-19 vaccination were associated with telehealth utilizatio**n.

**Discussion**: Access and integration of telehealth services were highlighted as challenges for this population of African Americans. To reduce disparities, **expansion of subsidized wireless internet access in marginalized communities is necessitated. Education outreach and training by healthcare systems and community health workers to improve uptake of telehealth currently and post-COVID-19 should be considere**d.

**Discussion:**

**The findings of this study revealed that increased perceived risk of COVID-19 and positive attitudes toward COVID-19 vaccination were associated with telehealth utilization**. In a sample of middle-aged and older African Americans with chronic health issues, our results showed participants who were **not Medicare beneficiaries, had home internet, possessed a cellular phone, and were chronologically older were more likely to utilize telehealth services.** Combined, these findings advance the literature for emphasizing the promising role **of relevant culturally sensitive, theory-based telehealth services and interventions as a tool to improve the health status of an under-resourced minority population.**

**Our findings revealed a significant relationship between older age and higher utilization of telehealth among this under-rescourced population**. Elam and colleagues (2022) uncovered that older adults in the US were less likely to have telehealth visits than in-person visits during the height of the COVID-19 pandemic [38]. However, older adults are likely to be interested in participating in a first-time telehealth visit [39], **which should prompt healthcare systems to offer more assistance and guidance for telehealth to the older population**. Additional multiple lines of data suggest that COVID-19 **was a particular threat to older adults with numerous comorbidities [40,41], which may have contributed to their interest in telehealth services**. It is important to note that 40% required assistance to join telehealth services, which should prompt providers to ensure that this population has comfort with telehealth **services or provide technological assistance.** Furthermore, undesired COVID-19 outcomes such as hospitalization are highest among older adults with underlying diseases [25], as COVID-19-related hospitalization and death rates increase with age [25]. Therefore, efforts to engage older adults in telehealth practices may be beneficial to increase their engagement.

This study showed that positive attitudes toward COVID-19 vaccination and higher **levels of perceived risk of a COVID-19 infection were associated with telehealth care utilization**. It has been evidenced that having a greater degree of understanding of COVID-19 was connected **with having more favorable views toward preventive actions, such as using a mask and adhering to social distancing measures. Compared to non-Hispanic Whites**, African Americans **were at an increased risk of experiencing severe COVID-19 outcomes [**42,43]. In addition, the increased incidence of severe COVID-19 among African Americans is partly due **to higher infection rates, which suggests that COVID-19 disparities most likely result from increased vulnerability** [42,43]. **Thus, those who may have perceived themselves at risk may have been more likely to communicate with a healthcare provider**.

Medical insurers have noted that there was an increase in telehealth billing claims for residential areas that are predominantly African American [2]. Our results supported this claim, with 19% reporting a video call with a provider, compared to 49% who had a telephonic visit with their provider. Our findings show that those who had strong attitudes toward protecting themselves against a COVID-19 infection were more likely to continue with care visits with their provider. However, 30% reported no usage of telehealth. **Therefore, it should be a priority that providers continue to assess attitudes toward COVID-19 and other major chronic illnesses**, and collective efforts should be made to increase positive messaging about COVID-19 and health status, especially during all telehealth and in-person care visits.

**Our results also revealed that having internet at home and having a cellular network were significantly associated with the utilization of telehealth**. A recent Pew Research Center report revealed the widening of the national digital divide, as those with lower incomes have **lower rates of ownership of a smartphone, home internet, or desktop/laptop/tablet computer, resulting in decreased technological access** [44]. In our entire study population, **25% indicated that they have no internet at home, and 38% had no cellular network on their cellphone.** One of the major obstacles to the widespread use of telehealth is the influence **of socioeconomic status**, resulting in lower access to and proficiency with various forms of technology, e**specially for impoverished populations**. **Some contributing factors include financial hardship, illiteracy, a lack of enthusiasm or desire, and an absence of necessary resources to make use of available technologies** [45,46]. A recent study found that compared to their White counterparts, African Americans were less likely to participate in telehealth and telemedicine visits [8]. The COVID-19 pandemic led to healthcare providers shifting to online and remote modalities for clinical care, as opposed to in-person visits [47].

Additionally, we documented that not being enrolled in Medicare was also associated with telehealth use. **Webber and colleagues found that individuals with commercial or private health insurance were likelier to have appropriate technological devices for telehealth use compared to Medicare patients with a prepaid phone or no phone at all** [48]. Further studies support our findings that **commercial insurance** holders utilize telehealth services more frequently than Medicare beneficiaries [49]. **During the COVID-19 pandemic, the Centers for Medicare and Medicaid Services approved telehealth waivers as a component of the Public Health Emergency** **framework**, which likely increased the utilization of telehealth services by Medicare beneficiaries [50]. Even though the offering of telehealth services among Medicare beneficiaries increased from 18% to 63%, pre-pandemic to November 2020, minorities and older adults **had less availability of telehealth equipment.** Additional studies reported lower adoption of telehealth services among African American and Latino Medicare beneficiaries [51].

Our data also showed a relationship between th**e total number of diagnosed chronic diseases and telehealth utilization at the bivariate level.** With only 30% of the study population rating their physical health as very good or excellent, **participants may have sought medical care via telehealth methods for their chronic health issues and quality of medical life.** Ng and Park (2021) **found that individuals with multiple comorbidities were likely to have telehealth services offered by their primary care provider compared to an in-person visit** [52]. Supporting evidence has confirmed our findings that individuals with an increasing number of comorbidities are **likely to be offered and use telehealth services** [53]. Moreover, telehealth interventions have benefited African Americans for various diseases, including diabetes and glycemic control [54], gastrointestinal diseases [55], and anxiety and depression [56]. Social isolation, combined with other risk factors such as depression, may contribute to worsening disease management for these individuals with chronic diseases [40]. Due to the consistent messaging of practicing social distancing, an increase in telehealth utilization and treat**ments may be forecasted as a preferred method for the management of chronic diseases. However, those with multiple comorbidities and psychosocial needs may struggle to engage in telehealth visits and face difficulty navigating services** [57]. It is crucial to study the effectiveness of treatments to know which chronic diseases may be managed remotely and which need personal contact.

**It is important to highlight that over two-thirds of the study population believed that the processes of scheduling and participating in a telehealth visit were not well integrated**. A study that explored telehealth perspectives among under-resourced communities in the South Los Angeles area found that African Americans were more concerned regarding th**e lack of privacy and confidentiality and the physical absence of the provider, compared to Latinos** [58]. This may contribute to our finding of 20% reporting that their questions were not answered during telehealth visits. Wegerman and colleagues (2021) propose that healthcare systems must assess and ensure that there **is full support for telehealth with both internal and external partners prior to implementation for integration ease** [59]. Various healthcare providers, including physicians and nurses, have exposed telehealth disparities in areas such as access to care, **bandwidth connectivity, availability of devices to perform telehealth, and socioeconomic and language barriers** [60]. Moreover, they discuss that smartphone **interfaces and funding for patient advocacy is essential for the success of telehealth.**

4.1. Implications for Programs, Practice, and Research

These findings have significant implications for issues such as the introduction of telehealth, patient satisfaction, and the interaction between healthcare practitioners and patients. In addition, telehealth can benefit under-resourced populations, including those experiencing **transportation challenges** **or other socioeconomic disparities**. Meeting the needs of African American older adults during the COVID-19 pandemic may show innovation that can be translational for local governments and traditional safety net providers within the social work milieu [61]. Moreover, access can be increased for these populations to have health encounters with providers of different specialties, as waiting times are likely to decrease.

To increase health equity, **health systems and providers must develop and continuously improve infrastructure for greater access and availability of telehealth for all individuals,** which includes compatibility of various technological devices for audio and video visits. With the increased usage of telehealth, many providers could maintain their clinical priorities while maintaining social distance from their patients during the height of the pandemic. As projected, telehealth will continue to rise significantly following the COVID-19 pandemic, necessitating expanded access and accessibility, especially for under-resourced minorities. Additionally, these telehealth visits should incorporate a multidisciplinary approach, in which various providers can meet with the patient in a single visit, such as the physician, pharmacist, and social worker.

Healthcare policies centered on incentivizing telehealth adoption for providers and patients should address the inequalities in mobile technology among under-resourced minorities. **Technological devices owned by lower-income individuals, such as prepaid cellphones, may have fewer features and poorer capability to access telehealth components, such as video call visits or reviewing their online health records.** Moreover, these populations may reside in communities with **environmental and structural inequalities, with little or no wireless internet services, also known as “WiFi deserts”**, primarily required for technological device use. Future policies should increase internet access, broad infrastructure, and available intelligent devices that will economically and technologically benefit under-resourced populations.

When establishing strategies to promote and implement telehealth in urban, underserved African American populations**, it will be crucial to examine their attitudes and receptiveness toward the healthcare innovation of telehealth**. The COVID-19 pandemic catalyzed fundamental shifts in healthcare delivery, leading to rapid integration in facilities frequented by marginalized populations and exacerbating health disparities [61]. Therefore, certain population groups, including our population of older African Americans and Latino groups, may have yet to receive in-person guidance on how to use telehealth services, learning on their own during the pandemic. This likely precipitated barriers toward telehealth, including specific patient factors, such as time **inconvenience, cognitive or sensory impairment, and lack of perceived benefit.** **However, this may be compounded by other chronic health issues faced by African American and Latino groups in South Los Angeles,** including **low back pain [62], poor nutritional status [63], and frequent emergency care utilization** [64]. Future research should explore racial or ethnic variations among patients who used various telehealth options during the COVID-19 outbreak. In addition, literature has found that racial/ethnic minorities and older adults are less likely to participate in telehealth [65,66,67,68]. As African American and Latino groups are underrepresented in studies surrounding telehealth, implementing interventions to improve clinical outcomes with telehealth care delivery methods may be effective.

**Due to the high vulnerability of this population, the reduction of COVID-19 risk and the management of chronic diseases in older African Americans with a propensity for multiple comorbidities require urgent attention**. Our national healthcare system was unprepared with the onset of the COVID-19 pandemic, which worsened the health status of under-resourced communities. As rates of COVID-19 are decreasing, providers are still managing the long-term effects of COVID-19, coupled with care management of other major chronic diseases. To combat future crises of new infectious diseases, innovative interventions to increase telehealth use among this population is critically warranted. As health-based technological systems and interfaces continue to advance rapidly, certain populations may not gain access to these innovations and instead face worsening health effects. Current telehealth methods, such as emailing/texting providers or sharing results from wearable technology are not utilized by all individuals, increasing disparities. **Efforts to increase telehealth utilization among older African Americans with multiple comorbidities in the context of COVID-19 are warranted to improve health access and status.**

**4.2. Limitations**

This study had several limitations. First, this study had a small sample size, which limits our confidence regarding the generalizability of findings, particularly negative associations. A second limitation was the study’s cross-sectional design. All our study variables were measured at an individual level, and we did not collect neighborhood-level data or data from the healthcare system. We also did not measure mistrust in the healthcare system nor any stigma associated with it. Also, this study did not assess the frequency of in-person visits with providers or availability of various types of delivery methods offered by their provider (e.g., in-person, telehealth, home visit). Moreover, we also did not differentiate between different chronic diseases. While we **pooled all chronic diseases together, some conditions may correlate differently to telehealth utilization**. Additionally, participant enrollment and data collection period lasted from March 2021 to August 2022, which occurred during various waves and surging strains of the COVID-19 pandemic. However, the COVID-19 vaccination had been nationally available since the inception of the study, and our study reflects attitudes and behaviors among an under-resourced group who has been negatively impacted overall with severe COVID-19 outcomes. According to the findings of this study, African Americans who live in different regions of the United States of America have varying rates of telehealth utilization in their respective regions. The findings of Adepoju and colleagues (2022) revealed minority groups like African Americans and Hispanics who lived further away from healthcare facilities found telehealth very beneficial [69]. However, our study focused on older African Americans, who reside in one of the under-resourced urban communities in the nation and face multiple other socioeconomic and health disparities [70,71,72,73]. **Moreover, the high expense of technology, inadequate internet connectivity, poor accessibility for the impaired, and the prevalence of low-performing gadgets all contribute to a general lack of access to technology.**

Study 9. Ennis et al. 2021

Link: <https://pubmed.ncbi.nlm.nih.gov/34312740/>

Abstract:

As the threat of COVID-19 on vulnerable populations continues, mitigation protocols have escalated the use of telehealth platforms, secure 2-way video platforms with audio capabilities. The goal of the current study was to examine factors associated with successful completion of video telehealth appointments in HIV care. We utilized a random effects logistic model to assess characteristics of patient encounters that predicted completed telehealth visits. Results show that factors such as identifying as black (AOR = 0.30, 95% CI 0.23-0.40, p < 0.01), identifying as heterosexual (AOR = 0.40, 95% CI, 0.29-0.55, p < 0.01), identifying as Hispanic/Latinx (AOR = 0.67, 95% CI, 0.48-0.95), having public insurance (e.g., Ryan White funding, Medicare/Medicaid) (AOR = .25, 95% CI 0.19-0.33, p < .001), and having detectable viral load (AOR = .049, 95% CI, 0.31-0.76) are negatively associated with completion of telehealth appointments. Results suggest that greater efforts to address the digital divide are needed to increase access to video telehealth

**Discussion:** This study sought to examine the factors associated with scheduling and completing video telehealth appointments. Results suggest that video telehealth appointments are a feasible alternative to in-person health visits for a certain subset of patients living with or at risk for HIV and is associated with optimal appointment adherence. However, there is clear evidence that not all patients in the CAN Community Health system have access to optimal telehealth services (i.e., 2-way secure audio and video digital connection) that allow healthcare professionals to visually examine, assess, and treat patients in the course of a routine visit. In the current sample, **approximately one-third of all video telehealth appointments were discontinued due to the patient’s lack of digital access needed to complete the appointment**. Factors associated with unsuccessful completion of a video telehealth appointment include: race, ethnicity, insurance status, viral load, and sexual orientation. These results support our hypothesis that race, ethnicity, and insurance status are primary factors associated with unsuccessful video telehealth appointment completion in this patient population. In the current study insurance status was used as a marker of socioeconomic status. Previous studies have demonstrated that patients of lower socioeconomic status and racial and ethnic minorities are more likely to miss HIV care appointment and have worse clinical outcomes [2, 4, 6, 19, 20]. In the current study, patients’ inability to connect to video telehealth appointments due to technology barriers led to incomplete video telehealth appointments that became phone-only appointments. Phone only appointments limit healthcare professionals’ ability to visually assess patient complaints. **Patients were adherent to their medical appointments and attempted to connect to the appointments but were then forced to convert the video telehealth appointment to phone-only appointments due to technology difficulties**. This demonstrates one way the digital divide can limit the range of services available to those without the technology needed to access 2-way secure video and audio platforms. T**hose who often have the most to gain from telehealth approaches are also the least likely to have access to broadband [21, 22] and/or cannot afford the necessary technology. Currently, approximately three-in-ten adults with household incomes below $30,000 a year do not own a smartphone, and more than four-in-ten do not have home broadband services (44%) or a computer (46%) [22] Additionally, at least 162 million Americans and 1.4 million Native Americans living on Tribal lands [23] have little-to-no broadband access.** The digital divide can be characterized as lack of access to a high-speed internet connection and the personal computers and smart phones needed to use the connection [24]. Lack of access to digital resources is generally due to a combination of factors such as: la**ck of access to broadband due to geographical location, lack of access to technology needed such as lower-performance computer, lower speed wireless connections, and lower-priced connections such as dial up [25, 26].The lack of access is problematic because of the rise in services such as video telehealth that require access to high speed reliable networks using adequately powered devices.**

Additional factors associated with completion of video telehealth appointments that we did not account for include age, viral load, and sexual orientation. Previous literature has found that older age is associated with greater challenges related to the use of digital services [27]. While older adults are not a monolithic group as it relates to internet usage, within a range of demographic sub-categories such as race, education, income, and sex, older adults have lower rates of internet usage compared to younger adults [28]. In the current sample, the finding that HIV status and sexual orientation are were associated with not completing video telehealth visits is likely due to socioeconomic resources. Patients who are HIV negative are seen for PrEP services which requires self-pay or private insurance in most cases, indicating greater socioeconomic resources. The finding that heterosexuals were less likely to complete a video telehealth appointment is unclear and likely due to a combination of factors such as race and socioeconomic status, and that heterosexual men tend to be less engaged in care [1]. While in this sample, those identifying as lesbian, gay, or homosexual were more likely to be white and have private insurance indicative of greater socioeconomic resources.

The finding that detectable viral load is associated with not completing a video telehealth appointment is supported by prior literature [13, 29, 30]. **In the US those of lower socioeconomic status are more likely to have detectable viral load due to lack of access to needed care** [19, 31]. While video telehealth appointment completion is not sufficient to achieve optimal treatment outcomes, it is a component of HIV care that may help improve clinical outcomes. Therefore, the relationship between unsuccessful video telehealth appointment completion and viral load suggests that access to care is an essential component to achieving optimal disease management. Results suggest that efforts are needed to increase access to video telehealth care and are warranted because it improves access to care.

Results of the current study should be viewed in light of certain study limitations. First, these analyses were based on EMR data, which is observational, and therefore cannot be used to establish causality. Second, the data reflects encounters of which the majority (92%) occurred in the state of Florida, which had a different approach than other states regarding lockdown restrictions. Generally, Florida eased lockdown restrictions faster than other states; therefore, patients returned to in clinic appointments at a faster rate (see supplementary Fig. 1 for details). Due to lack of power we were unable to run state by state comparisons. Finally, because patient income data was not available in the EMR data, insurance status was used as a proxy for socioeconomic status. Despite the limitations this study has noteworthy strengths. Study strengths include the use of a large community clinic encounter dataset with a diverse population. Additionally, due to the swift response of the CAN Community Health team, we were able to obtain data from the start of the pandemic and telehealth services in April 2020 through October 2020 when restrictions eased in Florida and throughout much of the country. Further, this study examined a wide range of income and racial ethnic backgrounds due to the CAN Community Health population served.

Study 10. Eruchalu et al. 2022

Link: <https://pubmed.ncbi.nlm.nih.gov/35213441/>

**Background:** Surgical patients with limited digital literacy may experience reduced telemedicine access. We investigated racial/ethnic and socioeconomic disparities in telemedicine compared with in-person surgical consultation during the coronavirus disease 2019 (COVID-19) pandemic.

**Study design:** Retrospective analysis of new visits within the Division of General & Gastrointestinal Surgery at an academic medical center occurring between March 24 through June 23, 2020 (Phase I, Massachusetts Public Health Emergency) and June 24 through December 31, 2020 (Phase II, relaxation of restrictions on healthcare operations) was performed. Visit modality (telemedicine/phone vs in-person) and demographic data were extracted. Bivariate analysis and multivariable logistic regression were performed to evaluate associations between patient characteristics and visit modality.

**Results:** During Phase I, 347 in-person and 638 virtual visits were completed. Multivariable modeling demonstrated no significant differences in virtual compared with in-person visit use across racial/ethnic or insurance groups. Among patients using virtual visits, Latinx patients were less likely to have video compared with audio-only visits than White patients (OR, 0.46; 95% CI 0.22-0.96). Black race and insurance type were not significant predictors of video use. During Phase II, 2,922 in-person and 1,001 virtual visits were completed. Multivariable modeling demonstrated that Black patients (OR, 1.52; 95% CI 1.12-2.06) were more likely to have virtual visits than White patients. No significant differences were observed across insurance types. **Among patients using virtual visits, race/ethnicity and insurance type were not significant predictors of video use.**

**Conclusion:** Black patients used telemedicine platforms more often than White patients during the second phase of the COVID-19 pandemic. Virtual consultation may help increase access to surgical care among traditionally under-resourced populations.

**Discussion:**

We evaluated demographic disparities in the use of virtual compared with in-person surgical consultation at a tertiary academic medical center during the Public Health Emergency (Phase I, March 24 through June 23, 2020) and second period (Phase II, June 24 through December 31, 2020) of the COVID-19 pandemic. Importantly, this analysis is among the first investigations of telemedicine use in general surgery after the initial COVID-19 Public Health Emergency. During Phase I, Latinx patients were significantly less likely to have video telemedicine visits than phone visits compared with White patients. Notably, we found that during Phase II, when surgical visit volume largely normalized, Black patients were more likely than White patients to use virtual surgical consultation. In both phases, insurance type was not significantly associated with visit type, and women were more likely to use virtual visits than men. During Phase II, older patients, patients with lower education level, and patients with non-English primary language were less likely to use video streaming during virtual visits.

Previous surgical literature investigating telemedicine access during the pandemic has largely focused on operational and logistical factors affecting the adoption of telemedicine and has been limited to the early Public Health Emergency period.11,15,16 In multi-center and national studies in orthopedic surgery, patients who are **Hispanic, low-income, have Medicaid insurance, and who speak languages other than English or Spanish were less likely to have telemedicine visits during the Public Health Emergency and were more likely to face barriers to navigating online platforms.**17,18 In a single-center study in otolaryngology, race/ethnicity was not significantly associated with visit modality during the Public Health Emergency, consistent with the present Phase I analysis.10 Patients with lower median household income, Medicaid insurance, and older age had decreased odds of virtual visit and video streaming use.10 In a single center analysis in general surgery, non-White patients were less likely to complete post-operative telehealth visits than White patients.19 T**hese disparities in telemedicine surgical consultation parallel inequities observed in general and subspecialty internal medicine, where racial/ethnic minorities, those with Medicare or Medicaid insurance, older patients, and patients with non-English primary language were less likely to have virtual visits and less likely to have video visits during the Public Health Emergency**.9,20-22 Although many of these previous findings were not apparent in the Phase I analysis, the Phase II analysis revealed that several of these disparities manifested in the general surgery population as the pandemic progressed.

As the healthcare system shifts to a new normal, the digital divide and structural discrimination against vulnerable groups may continue to impair access to surgical care and create inequities in telemedicine utilization.21,23 **Previous work has demonstrated that racial/ethnic minority and low-income patients are less likely to use the internet to obtain health information**.24 **Furthermore, patients who lack broadband internet access and those with lower digital literacy tend to have fewer telemedicine visits and are less likely to use patient portals to communicate with providers**.25-27 **Decreased use of video virtual visits by patients who are Latinx, older, have lower education level, and who have non-English primary language may reflect lower digital literacy and digital access in these populations**.2,21,24

***The associations between Medicare insurance and visit modality on bivariate analysis were likely mediated by older age of the Medicare population, because these associations did not persist after adjustment for age***.

Women have been repeatedly found to have increased engagement with mobile health platforms.9,10,28 This may reflect the relative convenience of attending virtual visits given a typically increased burden of domestic responsibilities in comparison with men.28

The novel finding that Black patients were more likely to use virtual surgical consultation during the Phase II period may reflect institutional efforts to expand digital health access for communities of color in addition to disproportionate access barriers to in-person care in this population. **The decreased logistical barriers to telemedicine participation make this visit modality more feasible for patients with significant time and resource constraints**.29 For example, telehealth platforms have been previously used to provide vascular surgery care to patients living in rural areas during initial consultation, perioperative visits, and long-term follow-up.30,31 In response to state and national policies that dramatically affected surgical care delivery in March 2020, this academic hospital implemented initiatives to reduce disparities in telemedicine access, address structural racism, and promote equity in patient-facing digital health platforms. These institutional efforts, which were enacted in spring 2020, may have contributed to increased virtual visit use among Black patients during the Phase II months. Of note, demographics of the patient population in the Division of General and Gastrointestinal Surgery at this academic hospital in 2020 were similar to previous years, before the implementation of these digital equity initiatives

The present work suggests that telemedicine platforms may provide a critical mechanism for racial/ethnic minority patients to maintain healthcare access during the pandemic. **Thus, policies to expand digital access and promote digital literacy in vulnerable communities are urgently needed to reduce disparities in telemedicine engagement and promote high-quality surgical care delivery during virtual visits,** particularly for patients who are under-resourced.23 For example, broadband subscribership and data charge subsidies will help socioeconomically disadvantaged patients engage with mobile health platforms.1 **Health systems can also collaborate with local and national governments to support policies that expand access to broadband internet and technological devices, which will enable participation in virtual visits.**3,21,32 **Health systems should also purposefully enroll under-resourced patients in telemedicine platforms. Digital literacy training as well as cultural and linguistic inclusivity in mobile health platform development will further help vulnerable patient populations engage with telemedicine.**23,32,33 **Finally, expanding reimbursement incentives for virtual surgical consultation, including phone visits and patient portal communications, will promote equitable telemedicine access**.6,34,35

Although digital health platforms may help expand access to under-resourced surgical populations, there may be clinical limitations to telemedicine. Virtual and in-person visits may not provide a comparable level of quality, particularly for new consultations or visits that require a site-sensitive physical exam. These potential limitations are likely exacerbated in an audio-only encounter. **Additionally, patients may have privacy or security concerns related to discussing health information over a virtual platform**.29 For follow-up surgical encounters, there is evidence that patient satisfaction and perceived visit quality are similar between virtual and in-person modalities.36-38 However, certain patients have expressed that trust and comfort with surgical providers are better cultivated by an in-person visit, and patients may prefer in-person evaluation for more complex surgical diseases.37,39 Notably, previous studies have also demonstrated similar post-discharge outcomes for patients who use virtual compared with in-person follow-up visits after low-risk surgical procedures.40,41 **Nevertheless, less is known regarding patient and provider perceptions of the quality of initial surgical consultation that is conducted through video or audio-only modalities. Institutional policies that promote digital access and literacy should focus on facilitating effective use of video-enabled technology.**

**Importantly, telemedicine use patterns continue to evolve during the pandemic. Virtual visit engagement is affected by patient and provider factors, which are influenced by the public health landscape as well as institutional, state, and national policies that govern healthcare delivery.** For example, telemedicine use dramatically increased after the Centers for Medicare and Medicaid began reimbursing virtual visits commensurate with in-person visits in March 2020.6 However, virtual visit use at this institution decreased during the Phase II months as the Department of Public Health relaxed restrictions on in-person consultation. As the pandemic progressed in 2021, virtual visit use at this institution has remained closely associated with healthcare policy, and video visits continue to provide an important tool for care delivery. **To promote equity, surgical providers can collaborate with their institutions and local policy makers to advocate for parity in reimbursement across visit modalities. Although providers and departments may become more selective in their use of digital health platforms as policies change, telemedicine will likely remain a valuable method of surgical care delivery.**

Limitations of this study include missingness of the race/ethnicity and education level variables in the database, which reduced the sample size available for the multivariable models and thus decreased the power of the analysis. Notably, our institution implemented systematic efforts to collect more robust patient demographic data early in the pandemic, which resulted in decreased proportions of missing race/ethnicity and education level data during Phase II. These equity-focused initiatives may also limit the generalizability of the results to healthcare systems in which similar efforts are not being performed. **Additionally, this study did not include qualitative analysis of the fundamental drivers of differences in visit modality usage. For example, increased engagement of Black patients with virtual consultation and increased use of phone visits by Latinx patients may reflect personal or cultural preference,** **concerns about potential COVID-19 exposure during a clinic visit, or privacy concerns regarding video visits**.29 Finally, the generalizability of these results is limited by the demographics of the patient population at this academic medical center. Similar to the overall Massachusetts population, this patient population is predominantly White, highly educated, and nearly universally insured, which limits the ability to analyze visit modality preferences among under-resourced groups.42,43

Future research should use qualitative methodology to analyze patient-, provider-, and system-level drivers of visit modality preferences among under-resourced general surgical patient populations, particularly racial/ethnic minority groups and patients with limited English proficiency. **Additional quantitative studies are needed to assess potential disparities in use of virtual surgical consultation in other healthcare systems, particularly those serving a larger proportion of racial/ethnic minority, non–English-speaking, and uninsured patients.** These studies should explore which initial visits can be effectively conducted virtually vs in-person from the clinical perspective. Future work should also examine the influence of community-level social factors, such as COVID-19 prevalence, on telemedicine use for surgical consultation across geographic regions.44 Further analysis of the second phase months, when surgical consultation volume returned to near baseline levels and surgical providers likely grew more comfortable incorporating virtual visits into their clinical practice, will be particularly illuminating.

Study 11. Esper et al. 2021

Link: [https://pubmed.ncbi.nlm.nih.gov/33725762](https://pubmed.ncbi.nlm.nih.gov/33725762/)1

## **Abstract**

**Objective:** Telemedicine has rapidly gained momentum in movement disorder neurology during the coronavirus disease (COVID-19) pandemic to preserve clinical care while mitigating the risks of in-person visits. We present data from the rapid implementation of virtual visits in a large, academic, movement disorder practice during the COVID-19 pandemic.

**Methods:** We describe the strategic shift to virtual visits and retrospectively examine elements that impacted the ability to switch to telemedicine visits using historical prepandemic in-person data as a comparator, including demographics, distance driven, and diagnosis distribution, with an additional focus on patients with deep brain stimulators.

**Results:** A total of 686 telemedicine visits were performed over a five-week period (60% of those previously scheduled for in-office visits). The average age of participants was 65 years, 45% were female, and 73% were Caucasian. Men were more likely to make the transition (p = 0.02). Telemedicine patients lived farther from the clinic than those seen in person (66.47 km vs. 42.16 km, p < 0.001), age was not associated with making the switch, and patient satisfaction did not change. **There was a significant shift in the distribution of movement disorder diagnoses seen by telemedicine compared to prepandemic in-person visits** (p < 0.001). Patients with deep brain stimulators were more likely to use telemedicine (11.5% vs. 7%, p < 0.001).

**Conclusion:** Telemedicine is feasible, viable and relevant in the care of movement disorder patients, although health care disparities appear evident for women and minorities. Patients with deep brain stimulators preferred telemedicine in our study. Further study is warranted to explore these findings.

**Discussion**

In this study, we demonstrated the successful and rapid implementation of a telemedicine healthcare delivery model across our Movement Disorders division, transitioning to virtual visits within one week and demonstrating a continuous and steady rise in the number of visits. Several factors contributed to this, **including the availability of centralized training modules, the documentation of standard work for all individuals participating in the visit process, and iteration over best practices within our group**. The 60% conversion rate of patients who were scheduled for in-person visits during this time was on par with all neurology subdivisions. Among the reasons that it was not higher a**re patient desire to wait for in-person appointment, lag in scheduling new patient visits to be able to accommodate existing patients of the practice and to minimize new testing (e.g., MRI) due to social distancing restrictions, being unable to contact the patient or surrogate, and not scheduling procedural visits that were not able to be performed by telemedicine.**

Contrary to prior reports [11,12], age did not play a role in converting to a telemedicine visit. In fact, the older age groups (70–79 years) and (60–69 years) comprised the largest patient populations, at 32.4% and 25.8%, respectively. This may be in part due to the older age of movement disorder populations. However, when the telemedicine subgroup was analyzed further, patients opting for a telephone visit were older than those opting for an audiovisual visit (p < 0.001). This finding may reflect the discomfort or inexperience of older patients with the audiovisual platform. Further studies regarding the impact of age in establishing telemedicine visits are warranted

Men were more likely to transition to a telemedicine visit. This trend has been previously demonstrated in a number of publications [13-16], particularly in developing countries where gender disparities may be more profound. It has been shown that some women face social barriers that may inhibit their participation in telemedicine [13], while in more rural settings, engaging in telemedicine visits can influence gender relations in a positive way by providing new modes of communication for a couple’s health as well as enabling greater male participation in health areas typically targeted towards women [14]. Further studies on gender disparities in telemedicine in developing countries would be of interest

We observed apparent healthcare disparities in access to telemedicine visits, as Blacks and minorities were less likely to engage in virtual care than Caucasians. It is well documented in the literature that racial/ethnic minorities who are socioeconomically disadvantaged face significant barriers to receiving healthcare [17-19]. Much of this disparity is thought to be due to lack of timely access to appropriate healthcare [13,20], and this is likely further compounded with subspecialty care that many movement disorders patients require. Additional problems may include access to technology to conduct visits, including both devices and broadband or cellular capability. We anticipate further study to define the reasons for these disparities so they can be mitigated.

Patients who chose to participate in a telemedicine visit were from a farther average distance than our prepandemic in-person patient population. This is of particular interest in movement disorder patients, where the combination of disease progression often leads to limited mobility and subsequent challenges with in-person visits, as well as limited access to specialty care, is paramount. Current care models often require travel to tertiary medical centers, increasing the burden on patients as well as caregivers [4,21]. Telemedicine improves access to trained subspecialists, allowing earlier diagnosis as well as skilled management of movement disorders [3,22]. Outcome studies for various movement disorder diseases using telemedicine care models would be an important area of future research

While the feasibility of telemedicine in PD has been previously demonstrated [5-8,23-26], our study implemented telemedicine across all movement disorders. The top diagnoses seen in our study included PD, ET, dystonia, and Huntington’s disease. As previously discussed, while no significant differences were observed among the diagnoses of patients opting for audiovisual vs. telephone visits, there was a significantly different shift in the distribution of diagnoses seen in the telemedicine period overall. However, this is likely partly driven by a longer baseline clinical period (12 months), which included many diseases that are infrequently seen and may have not been amenable to a telemedicine visit. This is of particular importance in dystonia patients, as many require botulinum toxin injections and were not included in this study. A longer study period would be indicated to determine if this finding is maintained over time.

For PD, the Movement Disorder Society-Unified Parkinson’s Disease Rating Scale (MDS-UPDRS) [27] is commonly used to document key features of the examination, including tremor, rigidity, bradykinesia, gait, and postural instability. While previous studies have confirmed the validity of remote assessments in PD [5,28-30], there are limitations. Specifically, rigidity cannot be assessed without touching the patient, and postural instability cannot be measured without a trained health professional. Most recently, Goetz et al. [31] reassessed their prior publication regarding handling missing values in the MDS-UPDRS [32] and concluded that the motor examination can accommodate the consistent loss of 3 values on any given visit to maintain a validated total score. While the instance of a telemedicine visit will result in 6 missing values (5 rigidity, 1 postural reflex), a very recent letter by the same authors in the setting of COVID-19 stated “We are very comfortable providing video-based or telemedicine care of high quality and compassion in PD.” Abdolahi et al. [33] conducted a study to compare whether administering the UPDRS without rigidity and postural instability would make a significant difference in trial outcomes. The results indicated that the same clinical outcomes were demonstrated, and high internal consistency was found. Another advantage of telemedicine in PD is the ability to observe patients in their own natural home environment [4], which can potentially help eliminate fall risks or other contributing factors.

**In our study, a higher percentage of patients with DBS chose to convert to telemedicine than patients without DBS. These findings may hint at the influence of disease severity, although we did not measure this variable directly.** DBS requires both clinical and surgical expertise, which requires advanced infrastructure and highly specialized skills and is usually limited to large urban centers. Additionally, of note, the population of DBS patients who converted to a telemedicine visit type was more representative of the typical historical prepandemic clinic population without a disparity in gender or race. This may reflect that to obtain DBS, patients must overcome typical barriers to care. In addition, the rapid cessation of DBS therapy due to battery failure in a time of limited surgical procedures during a pandemic can be life-threatening in certain cases [34-36]. Telemetry monitoring and remote care for device-based therapies are well established in cardiac pacemakers [24,37]; however, this technology is lacking in DBS programming devices. Two studies [38,39] confirmed the feasibility of using telemedicine for DBS in patients with PD. Moreover, both patients and physicians reported a high degree of satisfaction with telemedicine [38]. However, with the current technology, the changes allowed are limited to only checking the DBS settings via the patient programmer, as well as limited setting adjustments if patient parameters are set in advance, which is not always the case. This was of importance in our patient population during the pandemic, as in-person visits were unavailable and/or limited. Our providers were able to assess both PD and ET patients via telemedicine. Advanced technology in DBS hardware to truly program patients remotely is necessary in the future to fully implement a DBS telemedicine visit. It remains to be seen whether the shift towards telemedicine in DBS patients will continue to be seen as in-person visits resume.

Our finding that patient satisfaction was not compromised with the implementation of telemedicine as a care model is consistent with findings from multiple studies [8,26,29,40] that reported greater satisfaction in PD patients with telemedicine due to convenience and accessibility. Previous studies have also supported greater patient satisfaction with internet-based UPDRS assessment compared to in-person assessments [28].

The limitations of our study include a short study period of 5 weeks. A longer follow-up period would be helpful in adequately evaluating the benefit/limitations of telemedicine in movement disorders. In addition, further details regarding why certain patients opted out of a telemedicine visit would be useful to see if we can overcome whatever barriers exist and provide better specialized care to our patients. Last, more detailed patient and provider satisfaction variables are needed to adequately assess quality and patient satisfaction in a telemedicine movement disorder model. We plan to implement these changes to follow our patient population in a long-term prospective study.

In summary, telemedicine is both a viable and relevant care model in the treatment of movement disorder patients. If the policy factors that enabled telemedicine payments persist, it is likely that virtual care will become a mainstay of diagnosis and ongoing treatment. Additional models including provider-to-provider telemedicine for more rapid diagnosis and institution of therapy will become options as well. This would enable appropriate care and treatment sooner in the continuum of movement disorders with the opportunity to affect disease outcomes.

**Study 12: Friedman et al. 2022**

[**https://pmc.ncbi.nlm.nih.gov/articles/PMC9256787/**](https://pmc.ncbi.nlm.nih.gov/articles/PMC9256787/)

The COVID-19 pandemic resulted in widespread telehealth expansion. To determine telehealth uptake and potential sociodemographic differences in utilization among people with HIV (PwH), we examined HIV care appointments at the University of Chicago Medicine, an urban tertiary hospital. Visits between March 15th and September 9th for 2019 and 2020 were categorized as in-person, telehealth, and within telehealth, video, and phone. Differences in visit types were modeled using logistic regression to examine associations with demographics, insurance type, and HIV risk transmission category. **Telehealth appointments were more likely for those aged 46–60 versus those 31–45 [46–60; AOR 1.89 95% CI (1.14, 3.15)]. Black race and participants of other races were less likely to use telehealth compared to whites [Black: AOR 0.33 95% CI (0.16, 0.64), other: AOR 0.10 95% CI (0.02, 0.34)].** Future studies should continue to examine potential disparities in telehealth use among PwH, including age and racial differences

​​Discussion

In our examination of telehealth patterns and disparities among PwH during the COVID-19 pandemic, the number of PwH who attended in-person appointments was almost the same as those who were seen via telehealth. This ratio is similar to the mix of in-person and telehealth usage reported by **the Kaiser Family Foundation in their survey of Ryan White providers [**19]. Although other HIV care providers have expressed concern regarding possible disparities in telehealth usage among PwH or have measured their acceptability of telehealth, our study aimed to confirm and quantify these disparities [3, 20–23].

In our study, **we found that older persons were more likely to attend virtual visits compared to younger patients, unlike some studies of non-HIV populations [4, 7, 10]. However, other studies also found that older persons were more likely to use telehealth than younger people** [2, 8]. Additionally, we found that the preference for telehealth among older patients was driven by telephone rather than video visits, which has been seen in studies of the general population as well [4, 6, 8, 24]. **Older patients’ preference of telehealth over in-person visits could have resulted from early and persistent news coverage highlighting the risk of severe COVID-19 disease among older persons with comorbidities, as well as the unclear risk of contracting SARS-CoV-2 infection for PwH [25**]. The preference of these same patients for telephone rather than video telehealth visits could be due to discomfort with the newer technology of video calls as opposed to the more traditional audio phone calls [26, 27]. In our study, the age group that most preferred video visits was the one that contained patients aged 31–45 years.

We also observed racial disparities in terms of visit type, with Black patients and patients classified as other race being less likely to use telehealth compared to white patients. Our hospital serves a majority Black population from the south side of Chicago, an area with economic challenges that could have impacted appointment attendance and type. Lack of **internet may have prevented telehealth usage, while use of public transit to attend in-person appointments, as well as fears of increased SARS-CoV-2 risk associated with the hospital setting may have resulted in overall lack of healthcare utilization among PwH during the pandemic.** **It is also possible that PwH acquiring SARS-CoV-2 was a factor that drove telehealth use among PwH.** We have previously examined patterns in SARS-CoV-2 testing among PwH at our institution during this time period and found that overall SARS-CoV-2 positivity among PwH was 7.2% [28]. Most of these patients were Black (90.3%) and had a median age of 49 (34–59) years of age, suggesting to us that it is unlikely that infection with SARS CoV-2 is responsible for the racial disparity in telehealth use that we observed. Unfortunately, SARS CoV-2 test result data were not available in the dataset used for the current study, so we cannot confirm this finding.

Other studies have also found racial associations with appointment type in non-HIV populations, with white patients representing a higher proportion of telemedicine visits than other racial/ethnic groups [9] and Black race being associated with nearly twice the odds of completing a telephone visit versus a video visit when compared to whites [6]. For COVID-19 care in particular, fewer Black patients accessed telemedicine compared to white patients, even though Black and Latinx populations were at greater risk for COVID-19 morbidity and mortality [29]. T**hese racial and ethnic differences in telehealth use are likely due to structural factors including smart phone ownership (which is lower among Black patients) and lack of broadband access [30–32]. It may also be due to factors such as inability to obtain privacy at home or at work for a telehealth visit or cultural preference for in-person appointments**.

**These financial and technological barriers can be addressed by interventions at the federal, state and local level, as well as by promoting and incorporating feedback regarding telehealth services among disadvantaged minorities** [33].

Other than age and racial differences, we did not find any additional disparities among telehealth usage. These findings are reassuring, as additional telehealth barriers, such as technical issues, low technological literacy, lack of human connection, and concerns regarding privacy on sexual health matters could have presented problems for PwH [3, 20]. Moreover, it is also reassuring that we did not observe a change in insurance coverage between the pre-pandemic and pandemic periods, as has been reported by some HIV care sites [19]. **Other studies have found that patients with public insurance were less likely to use telemedicine** [5, 7] **and were more likely to have telephone encounters rather than video encounters** [6, 8, 24]. However, our findings suggest that Medicaid and Medicare coverage for telehealth successfully facilitated continuity of HIV care during the pandemic, which is critical given the high rate of Medicaid insurance utilization in our clinic [34]. We did observe some differences in appointments between the pre-pandemic and pandemic periods, including a decrease in appointments for patients aged 46–60 years in 2020, as well as reduced appointments among those whose HIV transmission category was other and an increase for those whose transmission category was unknown. It is possible that these effects were due to low sample size, especially for those whose risk was unknown, and should be examined further in larger HIV positive cohorts. In fact, we saw few demographic differences between study periods other than the decrease in appointments for patients aged 46–60 years in 2020.

**Our study was conducted over a longer time period (6 months) than other studies examining disparities in telehealth usage among the general population during the COVID-19 pandemic, which may have lessened the impact of the initial disparities seen at the sudden onset of the pandemi**c. However, our study highlights disparities that still remain after the initial implementation period of telehealth expansion at a large healthcare system. Furthermore, early in the pandemic, it was unclear whether PwH were uniquely vulnerable to SARS-CoV-2 infection, which may explain the decrease in appointment attendance, as well as appointment type.

Future studies should continue to examine disparities in telehealth usage, specifically among PwH. Most experts recommend keeping telehealth as a permanent option for HIV care [3] and including it when measuring retention in care [21]. Although our study found patients did not often utilize both in-person and telehealth visits, this observation likely resulted from suspension of normal laboratory testing of PwH during the pandemic [22]. Telehealth in non-pandemic times may be more interspersed with in-person visits to monitor CD4 T cell counts and HIV viral load levels.

**Study 13: Govier et al. 2022**

<https://www.ncbi.nlm.nih.gov/pmc/articles/PMC9012053/>

**Background**

Research exploring telehealth expansion during the COVID-19 pandemic has demonstrated that groups disproportionately impacted by COVID-19 also experience worse access to telehealth. However, this research has been cross-sectional or short in duration; geographically limited; has not accounted for pre-existing access disparities; and has not examined COVID-19 patients. We examined virtual primary care use by race/ethnicity and community social vulnerability among adults diagnosed with COVID-19 in a large, multi-state health system. We also assessed use of in-person primary care to understand whether disparities in virtual access may have been offset by improved in-person access.

**Methods**

Using a cohort design, electronic health records, and Centers for Disease Control and Prevention Social Vulnerability Index, we compared changes in virtual and in-person primary care use by race/ethnicity and community social vulnerability in the year before and after COVID-19 diagnosis. Our study population included 11,326 adult patients diagnosed with COVID-19 between March and July 2020. We estimated logistic regression models to examine likelihood of primary care use. In all regression models we computed robust standard errors; in adjusted models we controlled for demographic and health characteristics of patients.

**Results**

In a patient population of primarily Hispanic/Latino and non-Hispanic White individuals, and in which over half lived in socially vulnerable areas, likelihood of virtual primary care use increased from the year before to the year after COVID-19 diagnosis (3.6 to 10.3%); while in-person use remained stable (21.0 to 20.7%). In unadjusted and adjusted regression models, compared with White patients, Hispanic/Latino and other race/ethnicity patients were significantly less likely to use virtual care before and after COVID-19 diagnosis; Hispanic/Latino, Native Hawaiian/Pacific Islander, and other race/ethnicity patients, and patients living in socially vulnerable areas were also significantly less likely to use in-person care during these time periods.

**Conclusions**

Newly expanded virtual primary care has not equitably benefited individuals from racialized groups diagnosed with COVID-19, and virtual access disparities have not been offset by improved in-person access. Health systems should employ evidence-based strategies to equitably provide care, including representative provider networks; targeted, empowering outreach; co-developed culturally and linguistically appropriate tools and technologies; and provision of enabling resources and services.

**​​**Discussion:

Our study is one of the first to examine disparities in virtual and in-person primary care use among individuals diagnosed with COVID-19. Findings from our research suggest that, although there was a substantial increase in access to virtual primary care in the wake of the initial wave of COVID-19 infections and telehealth expansions, this did not substantially alter pre-pandemic disparities in access to primary care. Overall, only 12% and 27% of individuals diagnosed with COVID-19 used any virtual and in-person primary care, respectively, during the two-year study period. Disparities in use of virtual primary care were observed by race/ethnicity, and for some racialized groups, may have been compounded by concurrent disparities in use of in-person primary care. Specifically, Hispanic/Latino and non-Hispanic other race/ethnicity individuals were less likely to use virtual and in-person primary care compared with non-Hispanic White individuals in the year prior to COVID-19 diagnosis, and these disparities persisted in the year following COVID-19 diagnosis. Individuals who identified as Non-Hispanic NH/PI were no more likely to use virtual primary care compared with non-Hispanic White individuals, and were less likely to use in-person primary care. On the other hand, we found that during the study period, n**on-Hispanic Black individuals were more likely to use virtual primary care than non-Hispanic White individuals.** These differences were observed when accounting for community social vulnerability and after controlling for demographic and health characteristics.

Previous research on racial and ethnic disparities in access to virtual care during the pandemic provides mixed results. For example, some studies have found that Black individuals had less access to urgent and ambulatory telehealth encounters and family medicine telehealth visits, compared with White individuals [31, 32], while others have found that Black individuals were more likely than White individuals to self-report using telehealth generally because of the pandemic [52]. **However, previous studies examined shorter time periods or were narrower in geographic focus**. Moreover, previous studies included general patient populations **rather than those diagnosed with COVID-19, making direct comparisons to our own findings challenging.** In addition, they did not distinguish between Hispanic and non-Hispanic Black patients, and most did not distinguish types o**f virtual care use (e.g., primary care versus telehealth care more broadly),** further compounding comparison issues. That said, our study adds to the growing body of evidence demonstrating that White, **non-Hispanic individuals generally enjoyed greater access to virtual care than non-White and Hispanic/Latino individuals during the COVID-19 pandemic—a particularly concerning finding given that racialized groups generally experience greater chronic disease burden than White and non-Hispanic population**s [53] and increased COVID-19 infection rates, both of which may necessitate increased access to care. In addition, our study highlights what pre-pandemic research has shown: that racial/ethnic disparities in access to virtual care have existed since the advent of telehealth care, and rather than improving disparities through expansion of telehealth, the pandemic has only exacerbated and/or shed more light on them [48, 49, 54, 55].

Overall, we found no significant differences in use of virtual primary care by community social vulnerability. Yet, we did observe disparities in the use of in-person primary care that were not improved by increased access to virtual care. Specifically, regression analysis revealed that individuals living in areas characterized as **vulnerable based on minority status and language, and housing type and transportation were less likely to use in-person primary care and no more likely to use virtual primary care than individuals living in areas not characterized as vulnerable in these ways**. These findings held in both models that adjusted only for race/ethnicity and in models that additionally adjusted for age, sex, and chronic conditions.

To the best of our knowledge, previous studies have not used the CDC’s Social Vulnerability Index to explore disparities in access to virtual care during the pandemic. **However, existing studies have examined factors related to community social vulnerability, including increased poverty and rural geography, finding pandemic-related disparities in access to virtual care along these dimensions [32–34]. In addition, evidence has clearly demonstrated how mechanisms of social stratification at the community level (e.g., segregation, community disinvestment, geographical concentration of poverty)** result in differential access to care, and that community characteristics directly and indirectly shape what and where care is available, and the quality of that care [56]. Our findings support and add to existing literature on the association between community-level vulnerability and access to care, indicating that even after accounting for race/ethnicity and controlling for demographic and health characteristics, those living in areas characterized by greater rates of non-White and non-English speaking individuals, and by crowded housing and lack of transportation, experienced disparate access to primary care prior to and during the first year and a half of the pandemic.

Taken together, our findings highlight the urgent need to ensure equitable access to virtual (and in-person) primary care. This is particularly important given additional surges in cases and the emergence of new COVID-19 variants, all of which signal that the pandemic is not likely to end soon and that virtual care will remain an important care modality. Furthermore, there is a growing “care debt” that has the potential to lead to deleterious downstream consequences such as complications from unmanaged health conditions and incapacitation of an already overwhelmed healthcare system [29]. To create health systems that can effectively manage these contingencies, it will be crucial to transition telehealth services from a crisis intervention tool to an equitable and sustainable system for providing proactive patient care.

Evidence-based strategies exist for creating more equitable telehealth and primary care infrastructure [31]. **First, conducting targeted patient outreach and actively connecting with individuals and groups who experience barriers to care has been shown to improve access to and utilization of care [57]. For example, in a study by Ospina-Pinillos et al. [58], participatory design methods were used to tailor the website of a virtual mental health clinic to improve outreach to Spanish-speaking individuals, which led to adequate acceptability levels in the website’s homepage, and triage, booking, and video visit systems for Spanish-speakers, and also enabled the clinic to identify the need for tailored assessment tools and greater integration with Spanish-speaking services and communities.** In addition, a systematic review of interventions aimed at modifying the healthcare system to better outreach to and serve racialized groups and communities revealed that these interventions were associated with both improved processes of care delivery and reduced access disparities [59]. However, **health systems must first be able to identify those experiencing barriers to care and find ways to create meaningful connections with them. This necessitates leveraging our current understanding of the multiple intersecting individual and community factors affecting access to care and addressing them in outreach materials and methods**. Furthermore, **at a systems level, this means deconstructing current systems which are inherently racist, overtly discriminatory, and implicitly biased, and rebuilding them into more just and healing systems that are acceptable and comfortable for diverse patient populations. For virtual care, this also means conducting additional research on what constitutes effective and trustworthy outreach and communication to diverse populations** [31].

Next, developing representative provider networks can improve capacity of and access to care, while at the same time improving quality of care for underserved individuals and communities. **Racial/ethnic concordance between patients and providers is associated with improved use of preventive services, satisfaction with care, patient-provider communication quality, and patient participation in care and decision-making** [60, 61]. **In addition, evidence shows that clinicians from racialized groups are more likely to treat patients from racialized groups, including those who live in medically underserved and vulnerable areas** [62]. However, **policymakers and health systems must purposefully devote financial and other resources to improving provider representativeness and dismantling racist and discriminatory practices including those that have resulted in a current provider supply that is more White and socioeconomically advantaged than the general U.S. population** [63].

**Designing culturally appropriate tools and technology that enable and improve access requires adaptations to systems predominantly designed for White, English-speaking individuals**. To that end, **health systems can collect and incorporate input on telehealth tools and technologies from racialized groups and those with limited English proficiency [64], as evidence indicates that cultural and linguistic tailoring can improve healthcare access and outcomes** [58, 65–67]. **Data collection and user testing should be done in a participatory manner in which cultural adaptations, and knowledge and language translation are co-designed with patients and/or research participants** [58]. **Health systems can also increase robust adoption of the National Culturally and Linguistically Appropriate Services Standards developed by the U.S. Department of Health and Human Services [68], which are intended to provide health workers and systems with a blueprint for developing equitable, understandable, respectful systems of care.**

Policy solutions are also needed to address systemic barriers to care, such as inequitable distribution of healthcare and enabling resources. A recent survey found that limited broadband connectivity and related technology (e.g., computers and smart phones) has created barriers to telehealth during the pandemic [69]. This issue has particularly impacted individuals in rural areas and those over the age of 65. **One policy solution is to provide funding for broadband expansion in medically underserved communities. Several initiatives are underway to accomplish this: As part of the American Rescue Plan Act of 2021, the Federal Communications Commission is launching the $3.2 billion Emergency Broadband Benefit program to help Americans with qualifying household incomes obtain high-speed internet** [70]. In addition**, a $100 million federal pilot program has been implemented to cover eligible costs of broadband connectivity, network equipment, and information services needed to provide connected care services to patients; and the COVID-19 Telehealth Program included $200 million in Congressional appropriations to help healthcare providers provide connected care to patients at their homes or in mobile locations** [71, 72]. Time and future research will tell whether these policy solutions have reduced disparities in access to telehealth care.

Other policy and systems-level solutions that have been shown to improve access to primary care among underserved populations and communities include expanding scope of practice laws for and increasing the use of non-physician clinicians; expanding the supply of non-hospital-based clinics such as Federally Qualified Health Centers (FQHCs); increasing the availability of after-hours primary care services; and removing cost-related barriers to primary care such as cost-sharing [73].

Limitation

This study has some limitations worth noting. First, our study sample is limited to Providence patients in six mostly Mid−/Western U.S. states, which may limit generalizability to Southern and North−/Eastern states. That said, this study provides data on patients across a large, multi-state geographic area that includes both rural and urban areas, enhancing generalizability compared with existing research on smaller geographic areas and largely urban centers. Next, our sample is comprised of patients who tested positive for COVID-19, yet evidence has demonstrated disparities in COVID-19 testing rates among racialized groups and those with limited English proficiency, even as they experience higher COVID-19 infection rates [74–76]. Therefore, our sample likely does not include all Providence patients who contracted COVID-19. If patients from racialized groups who contracted COVID-19 were tested at a lesser rate than non-Hispanic white patients, our results likely underestimate disparities in access to care. Despite this, the fact that our COVID-19 positive sample was largely comprised of Hispanic/Latino patients while the larger Providence patient population is primarily comprised of non-Hispanic white patients enhances confidence in our findings. Finally, various issues arise in analyses of electronic health record data and should be taken into consideration when interpreting our findings. For example, if Providence patients received care outside of a Providence setting, it is not recorded in the EHR or included in our analyses. In addition, the EHR data does not contain information on other relevant factors such as socio-economic status or access to enabling resources. However, we did include census tract-level socioeconomic and resource-related variables via the SVI, and thus captured at least some of the variability in these factors and their association with access to care.

**Conclusion**

The pandemic has further illuminated the persistent inequities that lead to poorer access to care and health outcomes among racialized groups and vulnerable communities. Our study adds to the mounting body of evidence that lays bare these inequities. Using data from a large health system across multiple states, we found disparities in utilization of virtual and in-person primary care by both race/ethnicity and community social vulnerability among individuals diagnosed with COVID-19, some of the same groups of people who have been hit hardest by COVID-19 infections, morbidity, economic consequences, and mortality. The importance of primary care, together with widespread telehealth expansion brought about by the COVID-19 pandemic highlight both an urgent need and unprecedented opportunity to address these disparities, but only if solutions are purposefully designed and implemented to address their root causes [[31](https://www.ncbi.nlm.nih.gov/pmc/articles/PMC9012053/#CR31), [34](https://www.ncbi.nlm.nih.gov/pmc/articles/PMC9012053/#CR34)].

**Study 14: Grefe et al 2023**

<https://pubmed.ncbi.nlm.nih.gov/37186764/>

Abstract

**Background**: Children of minority race/ethnicity face barriers to accessing specialty services. During the COVID pandemic, health insurance companies reimbursed telehealth services. Our objective was to evaluate the effect of audio versus video visits on children's access to outpatient neurology services, particularly for Black children.

**Methods**: Using Electronic Health Record data, we collected information about children who had outpatient neurology appointments in a tertiary care children's hospital in North Carolina from March 10, 2020, to March 9, 2021. We used multivariable models to compare appointment outcomes (canceled vs completed, and missed vs completed) by visit type. We then conducted a similar evaluation for the subgroup of Black children.

**Results:** A total of 1250 children accounted for 3829 scheduled appointments. Audio users were more likely to be Black and Hispanic, and to have public health insurance than video users. Adjusted odds ratio (aOR) for appointments completed versus canceled was 10 for audio and 6 for video, compared to in-person appointments. Audio visits were twice as likely as in-person visits to be completed versus missed; video visits were not different. For the subgroup of Black children, aOR for appointments completed versus canceled for audio was 9 and video was 5, compared to in-person appointments. For Black children, audio visits were 3 times as likely as in-person visits to be completed versus missed; video visits were not different.

**Conclusions**: Audio visits improved access to pediatric neurology services, especially for Black children. Reversal of policies to reimburse audio visits could deepen the socioeconomic divide for children's access to neurology services

**Discussion**

Our study results point to the potential usefulness of audio visits in mitigating access problems for children needing outpatient neurology services. If flexibilities for audio visits were not an option during the pandemic, 59% of children in our study cohort would not have had pediatric neurology access for at least one appointment; 16% would not have had any pediatric neurology access. Our results show that audio visits are especially helpful for increasing access to pediatric neurology appointments for children of Black race.

We found that that both audio and video appointments, compared to in-person visits, are less likely to be canceled. However, only audio appointments are less likely to be missed when compared with in-person encounters. While there is support for telehealth use for pediatric neurology clinic visits,9,13,24,25 there is no consensus on which telehealth modality is best suited for different conditions or aspects of pediatric neurology care provision.9,10,26 Patients seen in pediatric neurology clinics may require frequent follow-up visits, typically for medication management for epilepsy, headaches, tic disorders or other chronic conditions. However, for these types of visits, video assessments may not inherently be necessary. **The best approach may be a shared decision-making model, wherein providers and families together decide on the best type of outpatient visit (audio vs. video vs. in-person) for the child given the type of care needed. Health systems caring for children should create structures and processes (such as clinic schedules and workflow) so that telehealth services remain an option for children for outpatient visits beyond the pandemic**. Canceled or missed appointments contribute to burden on providers and health systems as well.27 **Hence, flexibilities in the type of appointments offered (audio, video, or in-person) could reduce the strain on the health system by reducing canceled or missed appointments**.

Consistent with prior studies,11,13,14 our study shows that children of vulnerable groups – Black race, Hispanic ethnicity, and public insurance – are more likely to use audio visits than video visits. We also found that Black race and public insurance to be significant factors in missed appointments. Prior studies have noted Black patients have higher missed clinic appointments.19,20 Many factors including economic and logistic factors, mistrust in the healthcare system, and systemic racism have been attributed to these differences.10,14,28–30 In our study, **audio visits reduced the odds of missed appointments for all children; this association was more pronounced for children of Black race**. Similar moderating effect was not seen with video visits. Thus, our study provides evidence for the helpfulness of audio visits in reducing missed appointments for children, especially for those of Black race. Prior studies have shown that telehealth visit completion rates were higher for Black patients compared to White patients.14 **Thus, telehealth (especially audio encounter) has the potential to address some of the existing inequities in healthcare access**.

**As states are evaluating discontinuation of telehealth flexibilities offered during the pandemic, policy makers should consider the effect of reversing these flexibilities in worsening disparities in children’s access to specialty care. Additionally, continued lower reimbursement for audio visits compared to video visits could negatively affect access to specialty services for children belonging to vulnerable groups**.

Our study has certain limitations. EHR data were limited to a single institution and not generalizable to other institutions or regions. However, our study findings are consistent with pediatric and adult studies conducted in other regions of the country. Since data was obtained from one institution’s EHR, we could have missed children’s clinical encounters elsewhere. Also, we did not obtain detailed information about the encounter **(e.g. initial vs. follow-up, diagnoses associated with the encounter etc.) and could not determine how telehealth type varied by appointment characteristics. One of our study objectives was to understand the effect of audio visit on appointment outcomes specifically for children of Black race; however, there are well-established challenges in using race and ethnicity variables in EHR data.**31 Since this study used secondary data, **we did not have other socio-demographic variables that could have affected appointment outcomes**, **such as transportation, internet access, or ability to access EHR portal. We did not collect data specifically for research purposes.** Hence, it is possible that there were unmeasured confounders. Finally, since this is an observational study, the association between the type of telehealth and appointment outcomes do not indicate causation.

**Study 15: Jallow et al. 2023**

[**https://pmc.ncbi.nlm.nih.gov/articles/PMC9633105/**](https://pmc.ncbi.nlm.nih.gov/articles/PMC9633105/)

The COVID-19 pandemic has caused significant changes in dermatologic care, likely exacerbating health disparities for specific minority populations. The use of teledermatology has also become more prevalent during this period. The aim of this study was to determine if the proportion of teledermatology versus office-based visits varied significantly during three study periods of the COVID-19 pandemic. The secondary objective was to determine whether there are significant differences in the use of office-based dermatology versus teledermatology care across the following demographic subgroups: insurance type, race/ethnicity, age, and language during the same periods. A chart review of dermatology visits in electronic medical records at a tertiary referral center in Washington, DC, was conducted. The overall telehealth visit rate was 0% in the prequarantine period, 61.12% during the quarantine period, and 10.59% in the postquarantine period. After assessing telehealth utilization rates among the demographic subgroups, we noted that Medicaid users, Black patients, 64-year-olds or older, and English speakers may benefit the least from telehealth services. Teledermatology use necessitated by the COVID-19 pandemic may have promoted health care disparities for specific marginalized populations.

**Discussion**

Utilization by visit type

In the three months before the COVID-19 quarantine, telehealth was not used at the clinic sites included in this study; however, there was a significant overall shift to telehealth during the quarantine. Once mandates restricting in-person services were lifted in COVID-19 vaccine availability, there was an overall shift back to primarily office visits (89.41%) (Table 1).

Utilization by insurance type

Before the quarantine, private insurance users accessed dermatologic services at Medstar Health more than any other insurance group. Medicare users were the most underrepresented among the three major insurance types (Table 1).

Comparing telehealth visit rates versus office visit rates by insurance type across quarantine and postquarantine periods, our data revealed that the self-pay insurance group had the highest telehealth visit rate both during and after the quarantine (Fig. 1). This finding could be explained by the fact that telehealth visits are more cost-efficient than office visits, an important consideration for patients paying for services exclusively out of pocket.1 , 11 **Alternatively, the ​​Medicaid insurance type had the lowest telehealth visit rate (excluding other/unknown) both during and after the quarantine** (Fig. 1). **These findings suggest that the widespread use of telehealth instead of in-person visits during the pandemic decreased access to dermatologic care for the Medicaid insurance group. This finding is consistent with propositions in the literature suggesting disparities in access to telehealth among lower socioeconomic groups for whom this insurance type is intended**.1 , 5, 6, 7 Additionally, Medicare patients were the most represented in total telehealth visits during and after quarantine (Table 1). This finding could be a result of telemedicine services eliminating mobility challenges faced by the elderly populations and increasing accessibility of services for this subset of patients.1

Utilization by race/ethnicity

Prequarantine office visits broken down by race/ethnicity reveal that White patients had the lowest rate of telehealth usage during the quarantine (57.99%). In comparison, Black and Hispanic populations had the lowest rates of telehealth use in the postquarantine period (8.38% and 9.57%, respectively) (Fig. 2).

**Consistent with our hypothesis, these results emphasize the difficulties with maintaining access to technology in a group that is already considered to be disproportionately disadvantaged**.9 A cross-sectional study evaluating patient satisfaction with teledermatology showed that **non-White patients were more concerned with conversation privacy and inappropriate access to their data.**12 **Black and Hispanic patients are also less likely to own a computer or have broadband internet access at home.**9 Additionally, **negative cultural perceptions of the telemedicine model may influence their distrust of the telehealth environment**.6

**The Asian patient population had the highest telehealth visit rate during and after the quarantine (73.44% and 18.09%, respectively) (Fig. 2).** This finding is inconsistent with other findings in the literature suggesting that Asian populations have a lower desire for telehealth visits especially compared with their White counterparts.12 **This result may have occurred because the English-speaking Asian population has higher rates of technology-based, technically skilled jobs than the rest.9 Our data could reflect this population's comfort and familiarity with the abrupt transition to telemedicine technology.**

Utilization by language

Prequarantine office visits stratified by language reveal that prior to the quarantine, English speakers accessed dermatologic services at Medstar Health more than non-English speakers (Table 1). The non–English-speaking patient population had the highest telehealth visit utilization rate after the quarantine (13.5%) (Fig. 3).

**Our findings are inconsistent with other findings in the literature suggesting that non-English-speaking populations would have trouble connecting with telehealth care.8 One hypothesis is that these patients may find Medstar Health's teledermatology system relatively user-friendly.** **Virtual interpreters are a potential barrier for non-English speakers**. Therefore, having a robust medical interpretation system for telehealth visits is essential. Accessing medical translators (either through designated medical translator devices or via cell phone) during in-person office visits can be cumbersome, and using one can be time-consuming when aiming for smooth patient-provider communication.9 Ultimately, with a well-integrated and user-friendly virtual visit platform, non-English speakers may benefit from teledermatology.13

Utilization by age

To assess variations in telehealth use by age, the study population was stratified into three age groups. Patients between the ages of 19 and 64 comprise the most significant percentage of the patient population during the prequarantine period (Table 1). During the quarantine period, 0- to 18-year-olds used teledermatology at the lowest rate (79.61%) but used it at the highest rate after quarantine (20.39%) (Fig. 4). We posit that most of this age group are minors and likely cannot independently commute to their office visits. As a result, they may prefer telehealth visits as a more convenient option for their guardians.

During the postquarantine period, patients 64 years and older used telehealth at the lowest rates compared with their counterparts from other age groups (5.57%) (Fig. 4). This result may be because members of this age group are less likely to find comfort with new technology and prefer the less complex, traditional in-office visits when readily available. The transition to telehealth services during the COVID-19 pandemic may have created health care barriers for older age groups while benefiting younger age groups, further supporting the utility of maintaining teledermatology services and office visits in the future.1 , 14

Study limitations

The racial designations in our study were obtained from self-reported electronic medical records data. Approximately 20% of study participants categorized their race and ethnicity as unknown, making it impossible to draw conclusions from that subset. Similarly, 19% of study participants were non-English speaking patients whose primary language was unknown. For this group, it is impossible to make inferences about how language or translation access might have played a role in their telehealth utilization. As the literature describes, **the unknown population may mask health care disparities among minority groups**.15 Additionally, our results regarding ethnicity/race are specific to MedStar Health and do not necessarily reflect the ethnic representations in other health systems. Lastly, only synchronous teledermatology visits were included in this study, so the results from our study may not be generalizable to practices that utilize asynchronous or hybrid modalities of teledermatology.

Go to:

Conclusion

Use of teledermatology services during and after the quarantine differed significantly across the insurance, race/ethnicity, language, and age subgroups. Medicaid patients were the least represented in total telehealth visits during and after the quarantine period in this study. Black and Hispanic patients returned to traditional office visits during the postquarantine period at a higher rate than their counterparts from other races. The non–English-speaking patient population had the highest rate of telehealth visit use both during and after the quarantine. Patients over 64 years of age used telehealth at a lower rate than other age groups during and after the quarantine period. Specific patient groups such as Medicaid users, the elderly, and Black or Hispanic patients may experience fewer telehealth benefits than other patients.

Further studies are needed to characterize the demographic of patients most likely to access and benefit from telehealth services. Additional research regarding disaster preparedness and contingency planning for telehealth during future pandemics may be beneficial.

**Study 16: Kolb et al 2021**

[**https://pubmed.ncbi.nlm.nih.gov/33079014/**](https://pubmed.ncbi.nlm.nih.gov/33079014/)

**Objective**. To determine the rates and primary causes of missed appointments (MAs) for telehealth visits and present remedies for improvement.

**Methods**. This cross-sectional survey was conducted at a ter- tiary care pediatric otolaryngology practice during expan- sion of telehealth-based visits. A review of questionnaire responses was performed for 103 consecutive patients with MAs over 50 business days from March 20, 2020, to May 29, 2020. Families were asked a brief survey regarding the cause of the MA and assisted with technical support and resche- duling. MA rates and causes were analyzed.

**Results.** The overall MA rate during the initiation of tele- health services was significantly increased at 12.4% as com- pared with clinic-based visits of a similar duration before COVID of 5.2% (P \ .001). Technical issues were the most common causes of MAs (51.3%). Of the caregivers, 23.8% forgot or reported cancellation of the appointment. **Five percent of patients were non–English speaking and sched- uled without translator support**. **Minorities and patients with public insurance represented 53.6% and 61.9% of MAs, respectively.**

**Discussion.** Technical difficulties were the most commonly reported cause of missed telehealth appointments. **Optimization of applications by providing patient reminders, determining need for translator assistance, and reducing required upload/download speeds may significantly reduce rates of MAs and conversions to other communication**.

Implications for Practice. **Clear, concise education materials on the technical aspects of telehealth, platform optimization, and robust technical and administrative support may be necessary to reduce missed telehealth appointments and sup- port large-scale telehealth operations. An assessment of institutional capacity is critical when considering telehealth expansion.**

**Discussion**

No-show and MAs result in longer wait times for access to care, lost productivity, and higher health care costs. The University of California–San Francisco system estimated $7 million annually in lost revenue due to 67,000 no-show appointments. Another study cited a cost of approximately $210 in lost revenue for every no-show event.7,8 Office-based no-show appointments are frequently caused by patients forgetting their appointments and miscommunication. Multiple studies have demonstrated that patients with no-show appointments are more likely to be younger, be African American or Hispanic, have lower socioeconomic status, have Medicaid or public insurance, and have prior no-show activity. A longer distance of **residence from the clinic**, non- surgical appointments, and longer lead times prior to the appointment also correlate with higher no-show rates. 4,9

There are few studies on MA rates in otolaryngology practices and fewer in pediatric otolaryngology subspecialty practices. These studies show similar findings but also demonstrate higher no-show rates for adult female patients and **appointments before 9 AM and after 2 PM** in pediatric patients.10,11 Although some studies of adult practices demonstrate lower rates of no-show appointments in tele- health, the present study shows significantly higher rates of missed telehealth appointments in pediatric otolaryngology patients.5,6 However, the previous studies were also con- ducted in established telemedicine practices, which may have suffered similar growing pains during the initiation of this service.

Our study also supports previous findings that African Americans and patients with public insurance are more likely to miss appointments in a pediatric otolaryngology clinic setting. Although we were not able to compare our typical, pre-COVID telehealth population, a comparison of missed telehealth appointments and kept telehealth appointments suggests a disparity along similar lines, with African American and publicly insured patients demonstrating higher than expected rates of MAs (Table 2).

Compared with in-office patients with MAs, telehealth patients had similar race/ethnicity backgrounds and similar payer profiles. However, compared with the typical patient population at our institution, telehealth MAs were signifi- cantly more likely to be new **patients from minority groups with public insurance.** This may indicate that more new patients desired telehealth appointments during the COVID pandemic compared with pre-COVID operations and that new patients are more likely to miss telehealth appointments compared with established patients. It also appears that minority patients with public insurance are much more likely to miss telehealth appointments, which is similar to the office-based no-show demographics described in other stud- ies. These findings may be attributable to **education gaps and the inability to afford devices and Wi-Fi capability within the home. Further study into the cause of these potential socioeconomic and ethnic disparities is warranted.**

Technical problems represented the predominant reason for caregiver-reported reasons for no-show appointments. Many patients did not receive the **email containing the instructions for the download and setup of the telehealth platform (or app), which had been diverted to spam folders. Instructions were initially not available in languages other than English,** and **some caregivers were unable to understand the instructions for using the application.** Beginning on approximately telehealth day 8 after stay-at-home orders were issued, instructions in Spanish were included in the introductory email. In addition, **caregivers who spoke only Spanish were initially scheduled without translator support, which was later incorporated into the scheduling workflow on telehealth day** 34. **Three of 6 patients who missed multi- ple telehealth appointments were Hispanic or Latino, which may support the concern that language barriers were a frequent cause of MA**s.

**For caregivers who were able to access the virtual waiting room but unable to connect to the provider within the app, the optimization of telehealth platforms is necessary**. **The required upload and download speeds set by the telehealth platform may play a large role in the ability of patients to connect with and maintain the connection to their provid**er. **All major telehealth platforms use end-to-end encryption, which requires higher bandwidth and open network ports. CareConnect requires 3 Mbps for a stable 2-person (ie, physician and patient) connection but this may increase to 6 Mbps when a translator or other user is added into the call. Zoom** (San Jose, California) **and many other videoconferencing technologies require less than 1 Mbps, which correlates with higher rates of successful videoconferencing, but they do not have end-to-end encryption**.

Although download speeds are rarely a factor in success- ful telehealth videoconferencing, **one major Internet service provider in the United States guarantees only upload speeds of 768 kb/s for their least expensive Internet plan. This may significantly disadvantaged families with lower budgets for home Internet service. We recently upgraded our telehealth package to allow speeds as low as 2 Mbps when using a computer, but speeds are unchanged for phone and tablet devices**. **A backup platform for families incorporating a web link sent directly via text message, which will automatically connect to the patient waiting room and requires only 2 Mbps, may help to decrease the technology-driven issues in some families. T**his update will be initiated at our institu- tion in the near future.

**Other identified causes of technological failure include multiple devices using the Internet simultaneously, poor Wi- Fi connection, and firewall or antivirus software delaying, interrupting, or denying telehealth traffic. Providers and families should be given troubleshooting tips in their native language, including the use of hard-wired connections in lieu of Wi-Fi when possible, reducing firewall gateway settings to the lowest level that will allow peer-to-peer applications, using Wi-Fi with a minimum of 3 bars, moving devices closer to the router to avoid microwave and interference from other 2.4 GHz devices, and disconnecting other devices from the Internet during the telehealth visit. Education mate- rials regarding early application set up in advance of the appointment and the technological aspects of telehealth should be available to families to promote success.** **For fami- lies with large education or technologic gaps, technical sup- port assistance may be necessary for success.**

**Other institutions have been able to proactively contact telehealth patients who do not check in on time.** Under reduced manning, our division did not have sufficient man- power to directly contact each patient who missed a tele- health appointment. **We also did not have enough portable workstations to allow all office personnel to work from home. For patients new to telehealth, setting up the app with the patient can take 20 minutes or longer; therefore, it was not feasible to attempt the visit that day, even if families could be contacted. Since patients could be rescheduled within 24 to 72 hours in nearly all cases, it was likely not a significant problem for most families**. **It is noteworthy that even in a large tertiary care facility, there was insufficient manpower and portable workstations to optimize a large- scale telehealth practice. This problem would likely be com- pounded in smaller practices.**

**We also found that our technical support capability was initially overwhelmed by the large number of patients who did not receive or were unable to use the instructions.** Only 57.1% of patients who attempted to contact technical support were able to reach a representative. **Encouragement of set- ting the application up at the time of appointment scheduling may result in longer call times but decreased technical sup- port resources and late and MA rates.**

There was a high rate of caregivers who forgot about the scheduled appointments. Before the office appointments, families typically receive 2 text reminders at 3 to 5 days and 24 hours prior to the appointment. **At the onset of large-scale telehealth operations, families were sent an email to prepare them for the visit with instructions to assist with download- ing and setting up the application.** They were also sent another email reminder at 3 to 5 days prior to the appoint- ment; however, many patients were able to obtain appointments in shorter periods of time. **Patient reminders via both email and text messaging are encouraged to prevent the loss of reminder emails to spam.** **We later incorporated text reminders at 72 hours and 30 minutes prior to the appoint- ment to reduce MAs. Some studies showed decreased rates of missed office appointments when patients were scheduled within 2 to 3 weeks**12,13; however, further study is needed to assess the impact for telehealth appointments.

The limitations of the present study include the reliance on caregiver honesty and accuracy in assessing the primary cause of a MA, nonresponse bias, retrospective nature, and possible generalizability to all telehealth platforms and health care systems. **We acknowledge that these findings may vary depending on institution-specific telehealth plat- forms, registration, and scheduling processe**s. Because of multiple modifications made at various time points to dyna- mically improve the scheduling process and quality of CareConnect, we were not able to quantitatively assess the impact of the improvements, other than to demonstrate a trend of decreased conversion to alternative communication in patients with technical difficulties noted after a successful initial connection.

**Implications for Practice**

Technical difficulties were by far the most common reason for missed telehealth appointments. Clear, **concise education materials on the technical aspects of telehealth in the care- giver’s primary language may help to improve MA rates. Optimization of telehealth processes by providing patient reminders, application setup at the time of appointment scheduling, determining the need for translator assistance, and reducing the required upload and download speeds for tele- health platforms may significantly reduce the rates of MA and conversion to other communication.** **When considering telehealth expansion, an assessment of institutional capabil- ity is critical, as robust technical support capability may be necessary to address shortfalls in education.** **Disparities in access to telehealth care may exist for patients with public insurance or minority groups with language or technology barriers.** Although the trend of MAs improved over the short course of this study, future research is necessary to assess barriers to telehealth care and the impact of individual changes to the scheduling process.

**Study 17: Kummer et al. 2022**

[**https://pmc.ncbi.nlm.nih.gov/articles/PMC8873082/#:~:text=Results,up%20during%20the%20COVID%20period.**](https://pmc.ncbi.nlm.nih.gov/articles/PMC8873082/#:~:text=Results,up%20during%20the%20COVID%20period.)

**Background:** Patient groups traditionally affected by health disparities were less likely to use video teleneurology (TN) care during the initial COVID-19 pandemic surge in the United States. Whether this asymmetry persisted later in the pandemic or was accompanied with a loss of access to care remains unknown.

**Methods:** We conducted a retrospective cohort study using patient data from a multicenter healthcare system in New York City. We identified all established pediatric or adult neurology patients with at least two prior outpatient visits between June 16th, 2019 and March 15th, 2020 using our electronic medical record. For this established pre-COVID cohort, we identified telephone, in-person, video TN or emergency department visits and hospital admissions for any cause between March 16th and December 15th, 2020 (“COVID period”). We determined clinical, sociodemographic, income, and visit characteristics. Our primary outcome was video TN utilization, and our main secondary outcome was loss to follow-up during the COVID period. We used multivariable logistic regression to model the relationship between patient-level characteristics and both outcomes.

**Results:** We identified 23,714 unique visits during the COVID period, which corresponded to 14,170 established patients from our institutional Neurology clinics during the pre-COVID period. In our cohort, 4,944 (34.9%) utilized TN and 4,997 (35.3%) were entirely lost to follow-up during the COVID period. In the adjusted regression analysis, Black or African-American race [adjusted odds ratio (aOR) 0.60, 97.5%CI 0.52–0.70], non-English preferred language (aOR 0.49, 97.5%CI 0.39–0.61), Medicaid insurance (aOR 0.50, 97.5%CI 0.44–0.57), and Medicare insurance (aOR 0.73, 97.5%CI 0.65–0.83) had decreased odds of TN utilization. Older age (aOR 0.98, 97.5%CI 0.98–0.99), female sex (aOR 0.90 97.5%CI 0.83–0.99), and Medicaid insurance (aOR 0.78, 0.68–0.90) were associated with decreased odds of loss to follow-up.

**Conclusion**: In the first 9 months of the COVID-19 pandemic, we found sociodemographic patterns in TN utilization that were similar to those found very early in the pandemic. However, these sociodemographic characteristics were not associated with loss to follow-up, suggesting that lack of TN utilization may not have coincided with loss of access to care.

**Discussion**

In this retrospective study of over 14,000 established neurology clinic patients from a large, urban, multicenter, tertiary care health system in the 9 months prior to and following the onset of the COVID-19 pandemic in New York City, we found differences in TN utilization according to age, race, income, insurance coverage, comorbidity, preferred language, and utilization of ED care. **We found that only non-English preferred language, Black or African-American race, and Medicare or Medicaid insurance coverage were significantly associated with decreased odds of TN utilization during the pandemic.** Furthermore, we found that loss to follow-up differed according to age, sex, income, preferred language, and medical comorbidity. Interestingly, older age, female sex, and Medicaid insurance were significantly associated with a decreased odds of loss to follow-up. Additionally, we found that patients that had telephone, office, ED, or hospitalization visits during the COVID-19 pandemic were more likely to be Medicare-insured and harbor greater medical comorbidity than patients who did not use these care modalities.

The 34.9% rate of TN utilization we found is similar to that found in two recent studies, (14, 17) although the 14.8% telephone utilization rate we report is significantly lower than one study. This finding may be related to our design of excluding patients with only one visit, as well as these preceding studies’ smaller cohorts and earlier studied period during the COVID-19 pandemic. Our study builds on this prior work by including a longer follow-up period that includes both initial and later stages of the COVID-19 pandemic where in-person visits began occurring more regularly, and investigates multiple health utilization outcomes, including loss to follow-up. Furthermore, our study attempts to establish patterns of association between patient-level sociodemographic and clinical characteristics with TN utilization and loss to follow-up.

**We had initially hypothesized that patients who did not have any TN visits would more likely be older, non-White, non-English speaking, non-commercially insured, have greater medical comorbidity,** l**ive in areas with lower household incomes, and seek care through ED visits or hospital admissions** for care during the COVID pandemic than patients that had a TN visit.

Although non-TN utilizing population demonstrated all of the characteristics we had hypothesized, hospitalization rates were not different between TN and non-TN utilizing groups. We therefore could not accept our first hypothesis.

Despite this, many of our findings are consistent with prior investigations. **Notably, studies of neurological patient populations during the early COVID-19 surge have demonstrated that Black or African-American, (5, 14) lower- income, (5) and Medicare- or Medicaid-insured (14) patients were less likely to complete TN video visits rather than telephone visits.** **In another comparable study, patients who had telephone visits instead of TN visits were more likely to be older, non- commercially insured than patients evaluated by TN, with a pediatric subgroup being more likely to be non-English speaking** (17). Furthermore, two of the aforementioned studies were conducted in urban tertiary-care settings similar to ours, (5, 17) lending further credence to the generalizability of our results.

Similar studies in non-neurological populations (10, 22– 28) have shown consistent results with ours, with one study from a large urban health **system demonstrating that socially vulnerable populations were more likely to use ED care and office visits in favor of telemedicine care** (10). It is interesting to note that multiple studies conducted prior to the COVID- 19 pandemic have found that minority status was associated with increased odds of telemedicine utilization in comparison to White patient groups, (29–31) suggesting that our findings may be in part related to the extraordinary nature of the COVID-19 public health emergency. Nonetheless, taken together with results from previous studies, our findings underscore the presence of important asymmetries in TN access for traditionally disadvantaged patient populations during the COVID pandemic. These care asymmetries carry meaningful social consequences and require attention at a systemic level.

Importantly, our findings do not fully explain or identify the causes of the TN utilization asymmetries we observed. Contributing factors likely include existing, **inter-related digital and socioeconomic inequalities in the US healthcare system that clearly preceded the COVID-19 crisis**. **This digital divide has been shown to disproportionately affect the most disadvantaged patients in society, including ethnic minority, (32) elderly, (33, 34) economically disadvantaged,** (34, 35**) non-English speaking, and low health literacy patient groups (36).** **Concerningly, technology access gaps persisted during the early and middle phases of the COVID-19 crisis, with patients from disadvantaged populations continuing to demonstrate poor utilization of both telehealth** (37, 38) and digital technologies (39–41). Echoing the concerns with respect to global care equity raised by several authors during this period, (10, 12, 22, 34, 37, 40–43) our results, when taken together with the technological requirements of TN, may suggest that vulnerable patient populations may have been at a disadvantage during the larger shift to digital care platforms TN that occurred in the early COVID-19 pandemic.

However, the digital divide may not be the sole explanation for our results, particularly among Medicaid beneficiaries. At our institution, a diverse population of Medicaid-insured patients are treated in hospital-administered clinics by resident and fellow trainees under the supervision of attending neurologists (2). **Because many trainees were deployed to inpatient services for COVID-related care during the first several months of the COVID surge** (2) **and only returned to in-person office visits in June 2020, Medicaid-insured patients were likely unable to find available providers during the initial 3 months of the COVID pandemic between March and May 2020.** **This return to office visit care, combined with a preference for office over TN care, may also partially explain why Medicaid-insured patients were significantly less likely to be lost to follow-up during the COVID period.** Additionally, **hospital-administered and faculty practice clinics may have differed in the degree of TN platform on- boarding and technical support that was provided to patients and their caregivers to encourage familiarity with TN care. This may have driven some of the decreased TN utilization among Medicaid-insured patients.**

**Additionally, the lack of integrated translator services in our institutional TN platform during the early COVID-19 pandemic period could have been the cause of low utilization among non- English speaking populations. While translator services were available during this period, they were not integrated into the official institutional TN platform and required providers to access the services via a separate but concurrent telephone communication**. **Providers’ variable technology preferences and beliefs about telemedicine care may also have significantly influenced the degree of TN utilization.**

Similar to patients who did not utilize TN during the 9- month COVID study period, **we found that patients who were lost to follow-up were more likely to have a preferred language other than English and Medicaid insurance. Well-documented associations between limited health care access and reduced English proficiency, (42–46) Medicare or Medicaid insurance, and low income (47) may explain some of these commonalities.** However, our second regression analysis suggests that neither language preference nor income were **independently associated with loss of global access to care, and that patients with Medicaid insurance were in fact less likely than commercially-insured patients to be lost to follow-up during the pandemic** (Table 5).

Despite some overlap between these two patient groups, we found significant sociodemographic differences. In comparison to patients who were not lost to follow-up, those who had no visits during the COVID period were more likely to be younger, commercially-insured, and have lower degrees of medical comorbidity. One explanation for this is that the latter population may have reflected the demographic makeup of patients who migrated out of the New York City area during the COVID-19 pandemic. Although little has been documented about this population’s insurance coverage or degree of medical comorbidity, populations that migrated out of New York City have been shown to be relatively younger than populations that did not migrate (48). Additionally, patients who had few medical comorbidities may have been more likely to temporarily suspend their care than patients with greater comorbidities. Finally, this population may have also comprised patients who had less restrictive insurance plans or greater financial means and were therefore able to seek care at healthcare institutions other than ours in the New York City area during the COVID-19 pandemic.

The question of a potential relationship between TN utilization and loss to follow-up is also important for contextualizing our study’s results with respect to both individual outcomes. While we could not establish that low TN utilization definitively caused insufficient or absent follow-up, we did find that nearly half of the patients who did not have a TN visit were also lost to follow-up during the COVID period, and vice-versa. Because we defined follow-up to include ED and hospital visits for both neurological and non-neurological reasons, it is unclear whether low TN utilization truly co-occurred with loss of outpatient neurological follow-up. Despite our finding that publicly-insured, Black or African-American, and non-English speaking patients were significantly less likely to utilize TN than their commercially-insured, White, and English-speaking counterparts, these same patient factors were not significantly associated with loss to follow-up, suggesting that such patients received care through non-TN modalities.

The likely explanation for this is our finding that non- TN utilizing patients were significantly more likely than TN- utilizing patients to seek ED care during the COVID period. Additionally, patients who had more than one ED visit during the COVID period were also more likely to belong to vulnerable populations than their counterparts who did not present to the ED. Taken together, these findings are consistent with existing studies demonstrating that patients that preferentially used EDs for care over telehealth during the early COVID surge were more likely to belong to minority populations (10, 24). Reassuringly consistent with a recent study, (27) these groups were also well- represented among patients that had one or more office visits during the pandemic period, suggesting that despite lower TN utilization, populations that are historically affected by health disparities may have been able to preserve their access to their neurological providers through in-person, office encounters (Supplementary Table 3).

**Limitations**

This study was limited by several notable factors. First, the generalizability of our results may be limited, given the exceptional nature of the COVID-19 public health emergency and the resulting, unusually profound impacts on neurological care delivery. **Our analysis also lacked granular sociodemographic characteristics such as providers’ attitudes toward TN, patient domiciled status, access to caregivers or home assistance, and reliable access to Wi-Fi, smartphones, or computers**. We also could not differentiate those patients that presented to the ED or were hospitalized for neurological complaints, or those who were completely lost to neurological follow-up but may have presented to the ED or been hospitalized for non-neurological conditions. **Because we could not collect information relating to ED visits or hospitalizations at institutions other than ours from our clinical data warehouse, the rates of both these outcomes may have been understated.** Additionally, our analysis did not incorporate text data, including follow-up plans from visit progress notes. We therefore could not use this information to identify patients who were directed to follow-up after the end of the study period. However, to partially address this limitation, we identified a likely subgroup of such patients by using a discrete but less reliably populated field in our data warehouse.

In this retrospective cohort analysis of TN utilization at an urban tertiary-care Medical Center before and during the COVID-19 pandemic, we found that TN utilization varied according to race, income, insurance, and preferred language. By contrast, differences in loss to follow-up varied according to different, and times opposite patterns in the same factors. Importantly, none of these sociodemographic factors, with the exception of Medicaid insurance coverage, were significantly associated with loss to follow-up. This may suggest that low TN utilization may have coincided with, but not necessarily translated to loss of follow-up during the pandemic. Finally, we also found that populations with low TN utilization were more likely to use ED visits for care, and both groups had significant sociodemographic overlap, raising the possibility that the two may be causally related.

Further studies should incorporate granular data such as measures of patient education, provider attitudes, and technological literacy into analyses of TN utilization in order to better understand the causes of our findings. Future TN investigations should also study the effects of TN utilization on neurological care outcomes, as well as the optimization of TN care access among patients from different sociodemographic groups.

**DATA AVAILABILITY STATEMENT**

The datasets presented in this article are not readily available because this would jeopardize patient privacy. De-identified and/or anonymized data may be made available upon request by qualified investigators with sponsorship by an institution. Determination and/or approval from the Institutional Review Board of the Icahn School of Medicine at Mount Sinai as well as the receiving institution will be required. Requests to access the datasets should be directed to Benjamin R. Kummer, [benjamin.kummer@mountsinai.org](mailto:benjamin.kummer@mountsinai.org).

**Study 18: Lin C et al. 2023**

[**https://pubmed.ncbi.nlm.nih.gov/36609783/**](https://pubmed.ncbi.nlm.nih.gov/36609783/)

This study examined mental health service utilization and disparities during the first year of COVID. We analyzed data from all adult respondents with any mental illness in the past year (n = 6967) in the 2020 National Survey on Drug Use and Health to evaluate if mental health service utilization differed by geographic areas, race/ethnicity, and age groups. Only 46% of individuals with any mental illness had received mental health treatment. Compared to non-Hispanic Whites, Asian and Hispanics were less likely to receive outpatient services and prescription medicine. Rural residents received less outpatient treatment compared to large metropolitan residents. No difference was found in telemedicine utilization across area types and race/ethnicity groups. Older individuals were less likely to utilize telemedicine services. Our findings highlighted continued mental health treatment disparities among race/ethnic minorities and other sub-populations during COVID. Targeted strategies are warranted to allow older populations to benefit from telemedicine.

**Discussion**

The study highlighted the unmet mental health treatment needs during the first year of the COVID pandemic, that nationally less than half of the adult populations with mental illnesses received treatment to address their mental health issues. As reflected by the survey responses, some of the pre-existing challenges (e.g.**, low accessibility and affordability)** in mental health service utilization had unquestionably been exacerbated due to stay-at-home orders and the rapid shifting in healthcare service modalities during the early stage of the pandemic (Arevian et al., 2020; Bojdani et al., 2020; Busch & Kyanko, 2021; Mueller et al., 2021). The disruption in mental health care brought by COVID could not be completely solved by substituting in-person treatment with telemedicine (Costa et al., 2021; McDowell et al., 2021). **The low telemedicine utilization rate (~ 5%) reported in the survey may suggest only a supplementary role of telemedicine in mental health service provision during the first year of COVID. I**t is worth noting that the rate of telemedicine use reported in this survey was much lower compared to the number reported elsewhere (e.g., ~ 41% of behavioral health visits were reported to be conducted via telemedicine in October 2020; Mehrotra et al., 2020). The proportion of telemedicine use should be interpreted cautiously due to different survey question set up in the 2020 NDSUH survey, where internet/phone services were framed as additional sources of mental health care that were delivered in addition to inpatient, outpatient, and prescription medicine; therefore, some of the outpatient counseling and medication prescriptions delivered via the internet may not have been captured as telemedicine use in the study. Nonetheless, this finding calls for further studies to confirm the rate of telemedicine use in mental health care and strategies to enhance the role of telemedicine in mental health services.

The findings draw attention to the long-standing racial disparity in healthcare (Hines et al., 2017; Wu et al., 2018) that has been persistently manifested during the COVID pandemic. Non-Hispanic Asian and Hispanic respondents with mental illnesses consistently fell behind in all types of mental health treatment utilization as compared to non-Hispanic White. In addition to **the residential segregation and inequitable distribution of health-related resources (Yelton et al., 2022), the unmet mental health needs among Asian and Hispanic populations might be attributable to negative cultural beliefs about mental health and misconceptions of pharmaceutical treatment for mental illness** (Garcia et al., 2011; Givens et al., 2007; Lu et al., 2021). Surprisingly, no significant difference in telemedicine utilization across race/ethnic groups was found, possibly due to the lessened stigma-related concerns to receive telemedicine-delivered mental health care (Arafat et al., 2021; Fletcher et al., 2018). **This finding calls for a better understanding of diverse cultural groups’ concerns and preferences of mental health treatment, with which culturally competent strategies (such as ethnic matching and culturally tailored languages in assessment and counseling) can be devised to engage race/minority patients with mental illnesses in treatment** (Sue et al., 2012).

**A surprising finding is that residents in large metropolitan areas utilized less mental health treatment than those in small metropolitan areas. We speculate the reason for this phenomenon being the COVID crisis was initially concentrated in urban areas before it gradually spread to suburbs and then rural areas (Matheson et al., 2020), so urban residents, as compared to those in suburbs, might have avoided in-person healthcare services due to the fear of COVID exposure during the first year of the pandemic.** Although non-metropolitan and large metropolitan areas were not significantly different in any **mental health treatment, the gap in outpatient mental health treatment in rural areas identified in this study warrants attention and targeted approaches to address rural-specific service barriers, including the limited availability of specialty mental health care, lack of trained mental health providers, and underdeveloped care coordination in rural areas** (Andrilla et al., 2018; Kepley & Streeter, 2018; Morales et al., 2020; Myers, 2019). Researchers have raised concerns that inconsistent uptake of telemedicine in rural areas as it is in metropolitan areas will exacerbate the already wide disparity in access and quality of care (Summers-Gabr, 2020; Yang & Qi, 2022). However, no significant difference in additional telemedicine service utilization was found between types of areas. This null finding would somewhat serve to reduce the concerns about the negative impacts of the digital divide on rural mental health service disparity. **With enhanced broadband coverage, telemedicine could be a viable approach to increase access and alleviate mental health treatment disparity in rural areas** (Myers, 2019).

Older populations experienced disproportionally greater COVID-related challenges, including social isolation, fear of being infected, disruption of daily routine, and heightened risks of complications and mortality from COVID (Chen et al., 2021; Vahia et al., 2020). Therefore, they could benefit from telemedicine to reduce the commute burdens and COVID risks associated with in-person care (Beauchet et al., 2020). However, this study revealed less telemedicine service utilization among older patients**, possibly due to their greater difficulty adapting to internet technology (Lam et al., 2020; Ridout et al., 2021).** I**n addition, some older patients conceived telemedicine as incomplete or less rewarding compared to traditional in-person visits** (Aliberti et al., 2022; Ladin et al., 2021). **Therefore, compensated high-speed internet and technical assistance are necessary but not sufficient to bridge older patients to their needed mental health care; a thorough understanding of context-specific issues faced by older patients during telemedicine is warranted to develop strategies to promote equitable telemedicine-delivered services for vulnerable older patient**s (Gillie et al., 2022).

This study revealed other populations among whom mental health treatment and services were under-utilized during COVID. Disproportionately lower mental health service utilization in males was consistently reported in previous studies (Chang et al., 2019; Harris et al., 2015; Sagar-Ouriaghli et al., 2019), because of mental health service seeking are often perceived to be a sign of weakness, which is contradictory of traditional masculine gender role (Seidler et al., 2016). This finding suggests education efforts to increase mental health awareness and dispel misconceptions to improve mental health service utilization by men. Contradictory to previous findings (Rosenthal et al., 2012), **this study found that full-time employees utilized mental health treatment and services at a lower level than part-time employed or unemployed populations, with other covariates (including insurance and income) being controlled. Supported by literature (Dewa, 2014) and respondents’ reported reasons for not receiving treatment, full-time employees’ mental health treatment seeking may be deterred by workplace stigma towards mental illness and fear of damaging their career if disease status is inadvertently disclosed**. **Employers should provide a supportive environment and flexible work hours to encourage their employees’ mental health service utilization** (Giorgi et al., 2020). **Higher levels of mental health treatment and additional telemedicine service utilization were observed among respondents who experienced major depressive episodes and/or serious psychological distress**. **This finding can be explained by the Anderson Behavioral Model of Health Service, that a person needs factors, e.g., pre-existing health conditions, are predictive of their health service utilization (Anderson, 1995)**. **However, such an association was not observed among respondents with co-existing mental illness and SUD**. Since the co-occurrence of mental illness is a documented predictor of substance use relapse and overdose death (Evans et al., 2015), heightened efforts are needed to break the treatment-seeking barriers (Priester et al., 2016) and make mental health services available, accessible, and acceptable to this marginalized sub-population during and beyond COVID.

The study has several limitations. First, the cross-sectional design of the 2020 survey did not allow us to make any causal inference of the identified associations. Second, self-reports in NSDUH were subject to recall bias and social-desirability bias. Third, NSDUH excluded the homeless, military personnel on active duty, and residents of institutional group quarters, so the study findings cannot be generalized to these populations. Fourth, web-based screening/interviewing procedures employed in the 2020 NSDUH survey yielded lower response rates than in-person data collection (SAMHSA, 2021), as well as oversampling of tech-savvy respondents and over-estimation of additional telemedicine service utilization. In addition, the change of sampling method employed in 2020 limited our capacity to compare mental health utilization patterns to the pre-pandemic years. Fourth, the publicly available NSDUH dataset did not contain a calendar date variable nor a locator indicator, thus, we were unable to take into account COVID waves, local prevention policies, and their impact on the respondents’ treatment-seeking. Fifth, gender was dichotomized as male and female in the NSDUH dataset, so we were not able to examine mental health service use among transgender and non-binary people. Lastly, having AMI in the past year was characterized based on DSM-IV criteria in the NSDUH, which might be classified differently using DSM-5 criteria.

In conclusion, our findings highlighted continued mental health treatment disparities, especially among race/ethnic minorities, during the first year of the COVID pandemic. We suggest future research to investigate the influence of cultural factors on mental health serve-seeking and provision for certain race/ethnic minority groups. Although telemedicine-delivered mental health services may help to remediate these disparities, older populations with mental illness are in need of heightened support to take advantage of telemedicine. **This study also suggested the unmet mental health service needs among the male population, full-time employees, patients with insufficient insurance coverage, and patients with co-occurring SUDs.**

**Study 19: Mueller et al. 2022**

[**https://www.ncbi.nlm.nih.gov/pmc/articles/PMC9015206/**](https://www.ncbi.nlm.nih.gov/pmc/articles/PMC9015206/)

Introduction:

The shift from in-person visits to telehealth visits during the COVID-19 pandemic presented unique challenges for patients with pain. Disparities in health care access already existed, and the impact of telehealth on these inequities has not been studied.

Objectives:

To identify sociodemographic characteristics of patients with pain obtaining care through video, telephone, and in-person visits as social distancing restrictions evolved during the COVID-19 pandemic.

Methods:

Using our institutional clinical data warehouse, we identified 3314 patients with pain receiving care at a large academic institution in New York City during a baseline period (September 23, 2019–March 22, 2020) and counted telephone, video, and in-person visits during the following conditions: **a shutdown period (March 23, 2020–May 23, 2020), when nonessential in-person visits were strictly limited, and a reopening period (May 23, 2020–September 23, 2020), when restrictions were relaxed and in-person visits were available.** Patients were categorized into 4 groups based on the technology used to complete a visit: (1) video, (2) telephone, (3) in-person, and (4) no visit.

Results:

**Patients who were older, publicly insured, and identified as Black or Hispanic were overrepresented in the telephone visit group during shutdown and the in-person group during reopening. A** video visit during shutdown increased the likelihood of continued video visit use during reopening despite the return of in-person visits.

Conclusions:

Results show differences in how patients with pain accessed clinical care in a socially distanced world and that flexibility in method of health care delivery may reduce barriers to access. Future research will identify factors (eg, Internet access, digital literacy, provider–patient relationships) driving heterogeneity in telehealth use in patients with pain.

**Discussion:**

In this retrospective cohort study, we examined utilization of telehealth and in-person visits by patients living with pain at an academic center in NYC during the first wave of COVID-19. This study is the first to focus on patients living with pain and examine how telehealth use evolved as in-person visits became available. We report several novel findings. First, patients with pain who were older, Black, Hispanic, and publicly insured had an increased likelihood of accessing medical care (of any visit type) during both shutdown and reopening periods. Second, during the shutdown of nonurgent in-person visits, these patients were more likely to obtain care through telephone, not video, whereas during reopening, in-person visits predominated, and disparities in video visit use were mitigated**. Finally, a video visit during shutdown was a strong independent predictor of continued video use after in-person visits returned**.

In our population of patients with pain, we found that a greater proportion of patients who are Black and Hispanic received care during shutdown and reopening periods. This result was not expected and, to the best of our knowledge, not previously reported. Several reasons may account for this pattern. **Previous work has demonstrated that patients of minority race and ethnicity and those of lower socioeconomic status are more likely to experience severe pain,**31,39 which may increase the likelihood of seeking care. The biopsychosocial model of pain also highlights the important contribution of stress in modulating the individual pain experience**. The differential impact of COVID-19 on minorities and those with a lower socioeconomic status45 may have led to exacerbations of pain, increasing the likelihood of a medical visit**.1,28 **Finally, chronic medical conditions that are prevalent in people who are Black or Hispanic may have also an increased need for medical care.**16,22 The female predominance in our study population is consistent with previous work demonstrating an increased pain prevalence among female individuals relative to male individuals.19,32,33,41 We found a greater utilization of medical care by female pain patients during both shutdown and reopening. This finding is consistent with previous research showing male individuals may underutilize healthcare services, a pattern that may be exacerbated by the stigma of pain.30,34

**Previous research examining sociodemographic factors and use of telehealth demonstrate a context-dependent relationship**. For example, at a Midwest academic center, family medicine telehealth visits were used less often by racial and ethnic minority groups.37 At a Northeast academic institution, patients who identified as Asian showed lower utilization than patients who identified as Black or White.42 However, in a subgroup analysis comparing telephone and video visits, patients identified as Black were less likely to complete a video visit than those who identified as White.42 **Our study supports the importance of distinguishing between telephone and video visits.21,47 During shutdown, older patients, patients identified as Black and Hispanic, and publicly insured patients were significantly more likely to use telephone vs video than younger, White, privately insured patients.** Although this study does not establish the causes underlying these patterns of health care utilization, several hypotheses can be explored in future work. **Known disparities in broadband access and technology literacy may contribute to these patterns**.9 **In addition, people of lower socioeconomic status may have reduced access to a private space at work and home. An audio-only telehealth visit might afford greater privacy or flexibility and therefore be preferable for some patient**s.38

High utilization of telephone communication may have important consequences on health outcomes. **Despite the establishment of parity for telephone visits by the Centers for Medicare and Medicaid Services (CMS), telephone visits are associated with lower patient satisfaction and inferior communication of medical information in comparison with video visits.20,27,44 For patients with limited English proficiency, telephone visits are especially challenging.27 Visual information may be particularly important for providers caring for pain patients because nonverbal cues can provide insight into the individual pain experience.** Qualitative methods may allow a more nuanced understanding of the experiences felt by patients with pain during the COVID-19 pandemic when in-person visits were not an option.

Our findings provide additional insight beyond previously published studies by examining how disparities in telehealth evolved with time. During reopening, the disparity in telephone vs video visit use among patients with pain who were publicly insured and identified as Black and Hispanic was mitigated and among older patients abolished. **This is likely due to the development and implementation of outreach efforts that took time to execute and included assisting patients with portal activation and offering video visits through multiple platforms.** Previous work has demonstrated positive effects of such outreach efforts.18 **The strong relationship between video visit group during shutdown and continued video use during reopening aligns with the popularity of video visits and their continued availability despite a decreased need to social distance due to declining rates of COVID-19 infection. For patients with pain that limits mobility, TH may offer a significant benefit.**

Several reasons may account for disproportionate representation of patients who identify as Black or Hispanic in the in-person group during reopening. The importance of an in-person contact may have outweighed a potential increased risk of COVID-19 exposure. **Historical mistreatment and current systemic inequalities may contribute to a wariness of technological innovations by the medical community. In addition, there is evidence that face-to-face contact may play a more important role in therapeutic alliance and rapport building for race and ethnic minority individuals.**15,43

Our study had several limitations. First, our data lacked demographic characteristics associated with digital literacy such as level of education, preferred language, and income. Our data also lacked visit satisfaction and pain severity scores because this information was not uniformly collected. **Second, our data are from a single large health system and patients could have had encounters at a different health system. Third, our urban study population may limit the study's generalizability to rural areas where broadband Internet is not as readily available.** Fourth, due to the large number of medical specialties represented by our 33 EPIC-Net pain providers, a formal comparison of patients across provider disciplines would be confounded by individual provider practices. Finally, because almost all patients of Black and Hispanic race and ethnicity resided in NYC, the influence of a patient's geographic residence on telehealth utilization could not be examined.

In summary, our study characterizes how patients with pain used telephone, video, and in-person visits during the COVID-19 pandemic and describes how telehealth use patterns evolved as social distancing restrictions were relaxed and in-person visits became available. **This important information can be used to guide the formation and implementation of inclusive and flexible telehealth services and policies to prevent widening of existing disparities for patients with pain.** Future qualitative studies are needed to understand barriers and potential solutions to adoption of telehealth by patients with underrepresented pain.

**Study 20: Neeman et al. 2021**

<https://pmc.ncbi.nlm.nih.gov/articles/PMC9067360/>

The COVID-19 pandemic created an imperative to re-examine the role of telehealth in oncology. We studied trends and disparities in utilization of telehealth (video and telephone visits) and secure messaging (SM; ie, e-mail via portal/app), before and during the pandemic.

METHODS

Retrospective cohort study of hematology/oncology patient visits (telephone/video/office) and SM between January 1, 2019, and September 30, 2020, at Kaiser Permanente Northern California.

RESULTS

Among 334,666 visits and 1,161,239 SM, monthly average office visits decreased from 10,562 prepandemic to 1,769 during pandemic, telephone visits increased from 5,114 to 8,663, and video visits increased from 40 to 4,666. Monthly average SM increased from 50,788 to 64,315 since the pandemic began. Video visits were a significantly higher fraction of all visits (P < .01) in (1) younger patients (Generation Z 48%, Millennials 46%; Generation X 40%; Baby Boomers 34.4%; Silent Generation 24.5%); (2) patients with commercial insurance (39%) compared with Medicaid (32.7%) or Medicare (28.1%); (3) English speakers (33.7%) compared with those requiring an interpreter (24.5%); (4) patients who are Asian (35%) and non-Hispanic White (33.7%) compared with Black (30.1%) and Hispanic White (27.5%); (5) married/domestic partner patients (35%) compared with single/divorced/widowed (29.9%); (6) Charlson comorbidity index ≤ 3 (36.2%) compared with > 3 (31.3%); and (7) males (34.6%) compared with females (32.3%). Similar statistically significant SM utilization patterns were also seen.

CONCLUSION

In the pandemic era, hematology/oncology telehealth and SM use rapidly increased in a manner that is feasible and sustained. Possible disparities existed in video visit and SM use by age, insurance plan, language, race, ethnicity, marital status, comorbidities, and sex.

**Discussion**

Overall, the results of our study exhibited two main themes. First, in our health care system, the COVID-19 pandemic was associated with a rapid shift toward telehealth and away-from-office visits, while the patient and oncology provider populations remained steady. Similar reports were previously published by others27 and us,16,18,19 further supporting the feasibility and ubiquity of these changes.

Second, and more importantly, our study showed that secure messages and video visits were used less often by historically underserved populations such as the **elderly, disadvantaged or historically marginalized groups, those without commercial insurance, non-English speakers, and others**. Although all the comparisons included in this study are statistically significant (partly because of the large sample size), some of them are more clinically and/or operationally meaningful. For example, the small difference in video visit use between women and men (32.3% v 34.6%, respectively) is not deemed as meaningful as the much larger differences in video visit utilization of the various age groups. To the best of our knowledge, this is the first large study to describe disparities in use of telehealth and secure messages in medical hematology/oncology, across all types of cancers. Our findings were consistent with smaller prior studies that reported telehealth, secure-message, and EHR-portal enrollment-related disparities in various cancer-related disciplines,9-12,28 and they demonstrate that such disparities persisted despite the major shifts toward telehealth since COVID-19 emerged. Our study focused on patients in California, one of the most racially and ethnically diverse state in the United States (by US Census measures29), which outperforms most other states in government benchmarks of equity in health care.30 **Kaiser Permanente has promoted equity, inclusion, and diversity throughout its history, and regularly embeds mechanisms to identify and eliminate inequities in its operations,31 and its patient population reflects the racial and ethnic diversity of the population it serves.15 Thus, it is possible that our findings may in fact under-represent the severity of disparities in utilization of telehealth and secure message by patients with cancer across the nation.**

Surprisingly, among the various demographic groups, there were only subtle differences in the fraction of office visits out of all visits, and much more pronounced differences in the fraction of video versus telephone visits. This is in contrast to others' findings9 that also showed demographic disparities in use of office versus telehealth visit (video and telephone combined). In our health care system, medical assistants or navigators are in charge of booking patients for visits with hematology/oncology, and patients are not able to independently schedule their own appointments. **Although our study was not designed to answer this question, it is hypothesized that in our health care system, it is the provider or the system that commonly decides between office or telehealth visit, but it is the patient or patient characteristics that influence the choice between telephone and video visits.**

Our study was also one of the first to describe the volume of SM in oncology, showing that within our health care system, secure message utilization has been rising consistently before and even more so since the pandemic, and suggesting that use of secure messages is becoming a more prevalent method of care delivery in oncology. This study is somewhat limited in that we could not explore the content or themes of the included > 300,000 secure messages; however, we intend to report on this issue in future publications. SM technology is well received by patients32 and may reduce the need for more costly in-person or telehealth visits.14,33 However, the use of SM can have negative potential effects on provider well-being and work-life balance.34,35 Despite the above, secure messages are currently either not billed for or are compensated at a much lower rate compared with office or telehealth visits.36

**Regulation on reimbursement for telephone and video visits, by contrast, continues to evolve since the COVID-19 pandemic started, but in certain practice settings, these types of visits are reimbursed at a considerably lower rate than office visits.**2,18 The exemptions **regarding payment for telehealth by the Centers for Medicare and Medicaid are still considered temporary, and coverage and compensation rates for telehealth services by private insurers are governed by individual state regulations**.37 **This lack of payment parity between methods of care delivery may affect provider choices in how frequently to see each patient and by which method(s), and/or discourage providers from attending to secure messages in a timely and thoughtful manner, thus potentially leading to insufficient medical supervision with less use of telehealth and secure messages, or increased exposures to COVID-19 and other pathogens with office visits**. **Additionally, since telephone visits are reimbursed at a much lower rate than video visits in most settings, some providers may not offer telephone visits to their patients and, as such, inadvertently exclude access to telehealth for those who cannot use video. Thus, it is concerning that this lack of payment parity combined with the disparities in use of video visits and secure messages reported herein may disproportionately negatively affect clinical outcomes and quality of life for disadvantaged populations.**

There were several limitations to our study. First, it was conducted relatively early during the COVID-19 pandemic and thus may not fully represent the more recent or future evolutions in practice stemming from this pandemic. Second, our study was performed solely in Northern California, an area with one of the highest percentages of broadband internet access,38 and thus, our findings may be less relevant to rural and other areas with less pervasive access to broadband infrastructure. Third, the telehealth-related disparities noted in our study were based on the fraction of telehealth visits out of all visits but not on absolute numbers visits at the individual patient level. This may introduce a bias, as some patient groups may receive a similar fraction of video visits, but have higher or lower absolute number of video visits per patient compared with other groups of patients. However, the concordant disparities seen with absolute numbers of secure messages per patient make that concern less likely. Fourth, some of the demographic variables included herein (ie, race and ethnicity, and sex) were self-reported. There are many ways to describe these intricate social constructs; however, given the complex historical and societal connotations of sex, race, and ethnicity, we believe that self-report is the most appropriate way to describe/determine this information39. Finally, additional research is needed to measure the actual clinical impact of the disparities reported herein.

The future role of telehealth and secure messages in cancer care after the COVID-19 pandemic remains to be determined. However, several indications increasingly suggest that these technologies will continue to take a significant role in an emerging hybrid cancer care delivery model. T**hese include the rapid and sustained uptake of telehealth and secure messages as shown herein, high patient40-42 and provider16 satisfaction with telehealth, the lower-than-desired COVID-19 vaccination rates and ongoing infection waves,43 dispersion of broadband internet technology over time, and the recent recommendations for policymakers to extend current telehealth-related regulatory expansions.44 Indeed, ASCO has advocated for telemedicine policies to remain in place after the public health emergency ends.**3

As such, recognizing these disparities in telehealth and secure message utilizations is the first necessary step toward improving equity and inclusion in these realms of care. Specific improvement measures have been suggested by others, **including live interpreter services, education and tech support for patients and caregivers, working with local communities to identify public facilities with broadband access for patients to use, and teaching clinicians communication skills specific to telehealth**.45 The inherent characteristics of telehealth and SM technologies could be helpful in countering long-standing injustices and discrimination in health care by improving access to care and reducing logistic and financial burdens for caregivers and patients.3,45 Thus, overcoming the disparities in telehealth and secure messaging is a uniquely important and timely issue. Future research should focus on establishing the barriers to effective use of telehealth and secure messaging in various underserved populations, **understanding preferences of patients and caregivers from various demographic groups regarding the use of these technologies, and testing interventions to improve access and equity in telehealth and secure messages**. In our own health care system, improving equity in telehealth was chosen as the main patient-care equity goal for the department of Hematology and Oncology in 2021, and an extensive patient survey of patients' preferences and barriers relating to telehealth and secure messaging is underway.
