# Supplementary material for: Video-based telemedicine utilization patterns and associated factors among racial and ethnic minorities in the United States during the COVID-19 pandemic: A mixed-methods scoping review
Source: PLOS Digit Health. 2025 Jul 24;4(7):e0000952. doi: 10.1371/journal.pdig.0000952 (PMC12289041; doi:10.1371/journal.pdig.0000952)
Supplement: S8 File — (DOCX) [file pdig.0000952.s008.docx]

Paper 1:

Study 1. Bustamante et al. 2022

Link: <https://www.frontiersin.org/journals/public-health/articles/10.3389/fpubh.2023.1222203/full>

| **Thematic barriers** | | | | |
| --- | --- | --- | --- | --- |
| Race & insurance intersection | Access | Proficiency |  |  |
| Higher use of telehealth for R/E patients with commercial insurance relative to other types of insurance.  Uninsurance is a barrier for Black and Latina  Medicaid insurance is a barrier for Asians | Limited access to technology-related resources during the pandemic  Access to devices and broadband coverage  No access to healthcare due to low SES  Limited access in both commercial health systems and safety net environments | Asians adapted better to digital technologies compared to Black and Latino patients with higher rates of video visits  Discomfort and lack of familiarity  Digital literacy  English proficiency |  |  |

Study 2. Chen et al. 2021

Link: <https://www.ncbi.nlm.nih.gov/pmc/articles/PMC8415734/>

| **Thematic barriers** | | | | | |
| --- | --- | --- | --- | --- | --- |
| **Reimbursement** | **Systemic** | **Access** | **Proficiency** | **Intersectionality** | Facilitators |
| Lack of availability of telephone reimbursement can preclude patients for seeking care, patients who do not know about or are unable to use telemedicine (some for motor, literacy, or sensory reasons). | Racism and discrimination in medicine  Trust | Limited access to smartphone and broadband access (potentially exacerbated by the financial strain brought on by the pandemic).  Low SES and lower income were associated with phone use instead of video visit use.  Technology accessibility | Asians adapted better to digital technologies compared to Black and Latino patients with higher rates of video visits  Digital literacy  Technological proficiency  Telemedicine literacy | Patients with language barriers are excluded from telemedicine participation given lack of multilingual web applications and inconsistencies in the availability of translational service from set to appointment completion | Promotion and education of telehealth through local channels and networks to increase awareness ( churches, grocery stores, barbershops, bilingual mediums and non-electronic mediums such as newspapers). This may help to increase trust, literacy and accessibility.  Establishing regulatory bodies of federal policies to enhance the privacy of patient data, particularly in virtual mediums such as consuming-facing apps and devices.  Increase device access among disadvantaged patient populations  (included subsidies for internet access and insurance coverage of video-enabled telecommunication devices.) |

Study 3. Chen et al. 2023

Link: <https://pubmed.ncbi.nlm.nih.gov/37308801/>

| **Thematic barriers** | | | |
| --- | --- | --- | --- |
| **Patient-level** | **Provider and system-level** | **Access** | **Proficiency** |
| Patient preferences are deterministic of adoption patterns.  (no differences in the selection of telehealth in one period and changes in preferences where those with 2 or more comorbidities preferred in-person visits over telehealth visits.)  With exposure, people can learn over time to become telehealth users despite differences relating to demographic variations | Biases related to clinician comfort with telehealth and workflow practices.  -related issues such as the design of telehealth platforms  The presence or absence of critical functions for diverse patients, clinicians, staff user populations, including user interpreter integration and simple user experience.  Beyond this, more clinician and staff training and usage of structured assessments for patients' telehealth capabilities or preferences may help to minimize variation in practice due to biases from within the health system. | Digital Access | Literacy |

Study 4. Chumbler et al. 2022

Link: <https://pubmed.ncbi.nlm.nih.gov/36895424/>

| **Thematic barriers** | |  |  |  |  |
| --- | --- | --- | --- | --- | --- |
| **Access** | **Physical health** |  |  |  |  |
| Broadband and digital device access | Patients with comorbidities had greater odds of using telehealth services |  |  |  |  |

Study 5. Chunara et al. 2021

Link: <https://www.ncbi.nlm.nih.gov/pmc/articles/PMC7499631/>

| **Thematic barriers** | | | | | |  |
| --- | --- | --- | --- | --- | --- | --- |
| **Digital infrastructure** | **Systemic** | **Access** | **Proficiency** | **Intersectionality** | Provider level | Patient level |
| Telemedicine technical capacities:  technical glitches  compatibility/interoperability challenges  Provider technical competence) from an institutional perspective was likely minimized | Racism and discrimination in medicine  Agism  Trust  bias  Cultural competencies  Provider representativeness  the continuing collection of data on disparities and publication of results; the systematic identification of biases and the development of educational and training programs to address them; and removing as much individual discretion of providers as possible (eg, specifically during this period, standardized and ready testing availability would help). | Access to resources | Health literacy | Age and race barriers resulting from ageism and racism  Spanish-speaking patients had lower odds of telemedicine use | Availability of telemedicine-capable clinicians | insurance status  coverage type  knowledge,  attitudes,  cultural beliefs,  health behaviors,  Adherence  language  health literacy  social support  religious beliefs  self-efficacy  preference  psychosocial factors |

| Community-level | Marketing an awareness | Digital infrastructure (facilitator) | Implementation |  |
| --- | --- | --- | --- | --- |
| Mean income and median household size | These programs were heavily invested in and socialized to patients during both the pre-pandemic and acute pandemic periods.  positive targeted outreach, and research  communication of information on the use and availability of telemedicine could be studied in terms of how it may appeal to diverse populations in trustworthy ways | digital patient platforms  high levels of patient portal  (MyChart) participation,  Robust telemedicine program  Culturally and structurally appropriate tools and technologies | Facilitators  methods that leverage participatory design: cultural and linguistic adaptations can aid in helping patients manage online systems  Prior studies have included patient and community participants to culturally tailor online systems |  |

Study 6. Cousins et al. 2022

Link: <https://pubmed.ncbi.nlm.nih.gov/36395112/>

| **Thematic barriers** | | | | |  |
| --- | --- | --- | --- | --- | --- |
| **Note: “**Patient and provider skills as well as payor guidance are all in flux along with the pace of the pandemic itself.” | | | | |  |
| **Payor practices** | **Institutional level** | **Access** | **Distance** | Provider level | Patient level |
| Payor practices have guided telehealth use and enabled dramatic increases in telehealth use | Technological resources  Telehealth expertise  Providers and institutions might also have varied skill sets and resources to enable patients’ efforts to engage in telehealth | Internet (broadband?)  educational initiatives | Increasing distance was perhaps greater perceived incentive in the form of avoiding a lengthy trip to receive care**.**  Those who are distally located from the clinic may like to plan ahead prior to virtual visits with providers in hopes of avoiding the extra cost and effort required to attend visits in person.  Individuals living in remote areas were more likely to have engaged in video visits with another system prior to their encounter in our system | Providers and institutions might also have varied skill sets and resources to enable patients’ efforts to engage in telehealth | Patients can be taught, so it is likely that some patients might have gained the capacity to complete telehealth visits during the pandemic |

Study 7. Duan et al. 2022

Link: <https://www.ncbi.nlm.nih.gov/pmc/articles/PMC9115291/>

| **Thematic barriers** | | |  |
| --- | --- | --- | --- |
| **Note:** | | |  |
| **Systemic/ racism** | **Access** | **Intersectionality** | **Patient level** |
| Structural racism may contribute to lower technology access and digital literacy in Black communities  Mistrust of the health care system and technology in Black communities | Access to video‐enabled devices and digital literacy  Lack of activated patient portals and email addresses | We found that patients who were Hispanic/Latino and non‐English speaking were disproportionately affected by the COVID‐19 pandemic | Patients with public insurance had significantly lower odds of using telemedicine despite widely expanded coverage for telehealth services |

Study 8. Ekwegh et al. 2023

Link: <https://www.ncbi.nlm.nih.gov/pmc/articles/PMC9915549/>

| **Thematic barriers** | | | | |  |
| --- | --- | --- | --- | --- | --- |
| **System level** | **Access** | **Proficiency** | **Intersectionality** | **Provider level** | **Patient level** |
| The Centers for Medicare and Medicaid Services approved telehealth waivers as a component of the Public Health Emergency framework, which likely increased the utilization of telehealth services by Medicare beneficiaries | Access to a cellular network on a mobile device, and wireless internet at home were significantly associated with the utilization of telehealth care  Lower incomes have lower rates of ownership of a smartphone, home internet, or desktop/laptop/tablet computer, resulting in decreased technological access  Socioeconomic status, resulting in lower access to and proficiency with various forms of technology, especially for impoverished populations.  Being enrolled in Medicare was also associated with telehealth use. Webber and colleagues found that individuals with commercial or private health insurance were likelier to have appropriate technological devices for telehealth use compared to Medicare patients with a prepaid phone or no phone at all  Bandwidth connectivity, availability of devices to perform telehealth, and socioeconomic and language barriers  Technological devices owned by lower-income individuals, such as prepaid cellphones, may have fewer features and poorer capability to access telehealth components, such as video call visits or reviewing their online health records.  Populations live in communities with environmental and structural inequalities, with little or no wireless internet services, also known as “WiFi deserts  Moreover, the high expense of technology, inadequate internet connectivity, poor accessibility for the impaired, and the prevalence of low-performing gadgets all contribute to a general lack of access to technology | One of the major obstacles of telehealth use is the influence of socioeconomic status, resulting in lower access to and proficiency with various forms of technology, especially for impoverished populations.  Illiteracy | Age and race and ethnicity. Significant findings between older age and higher telemedicine utilization among this under-resourced population  Chronic illness and race and ethnicity: Increased telemedicine and were more likely to be offered. |  | Perceived threat of COVID-19 and attitudes towards the vaccine mitigated telemedicine use  A lack of enthusiasm or desire  A study that explored telehealth perspectives among under-resourced communities in the South Los Angeles area found that African Americans were more concerned regarding th**e** lack of privacy and confidentiality and the physical absence of the provider, compared to Latinos  Lack of preparation in how to use telehealth may have imposed barriers, such as inconvenience, cognitive or sensory impairment, and lack of perceived benefit.  However, this may be compounded by other chronic health issues faced by African American and Latino groups in South Los Angeles, including low back pain [62], poor nutritional status [63], and frequent emergency care utilization [ |

| Thematic facilitators: | Thematic facilitator cont’d |  |
| --- | --- | --- |
| Expansion of subsidized wireless internet access in marginalized communities is necessitated.  Education outreach and training by healthcare systems and community health workers to improve uptake of telehealth currently and post-COVID-19 should be considered.  Relevant culturally sensitive theory-based telehealth services  Interventions  40% of middle-aged and older African Americans required assistance to join telehealth services, which should prompt providers to ensure that this population has comfort with telehealth services or provide technological assistance.  ensure that there is full support for telehealth with both internal and external partners prior to implementation for integration ease [59]. Various healthcare providers  Health systems and providers must develop and continuously improve infrastructure for greater access and availability of telehealth for all individuals, which includes compatibility of various technological devices for audio and video visits  Telehealth visits should incorporate a multidisciplinary approach, in which various providers can meet with the patient in a single visit, such as the physician, pharmacist, and social worker. | Future policies should increase internet access, broad infrastructure, and available intelligent devices that will economically and technologically benefit under-resourced populations.  it will be crucial to examine their attitudes and receptiveness toward the healthcare innovation of telehealth. |  |

Study 9. Ennis et al. 2021

Link: <https://pubmed.ncbi.nlm.nih.gov/34312740/>

| **Thematic barriers** | |  |  |  |  |
| --- | --- | --- | --- | --- | --- |
| **System level** | **Access** | **Proficiency** | **Intersectionality** | **Provider level** | **Patient level** |
| Insurance status  Disease severity (HIV viral load)  In the US those of lower socioeconomic status are more likely to have detectable viral load due to lack of access to needed care  Race  Sexual orientation | Patients of lower **socioeconomic status** and racial and ethnic minorities are more likely to miss HIV care appointments and have worse clinical outcomes  Lack of digital access  Patients were adherent to their medical appointments and attempted to connect to the appointments but were then forced to convert the video telehealth appointment **to phone-only appointments due to technology difficulties**  Those who often have the most to gain from telehealth approaches are also the least likely to have access to broadband [21, 22] and/or cannot afford the necessary technology. Currently, approximately three-in-ten adults with household incomes below $30,000 a year do not own a smartphone, and more than four-in-ten do not have home broadband services (44%) or a computer (46%) [22] Additionally, at least 162 million Americans and 1.4 million Native Americans living on Tribal lands [23] have little-to-no broadband access.  Lack of access to broadband due to geographical location, lack of access to technology needed such as lower-performance computer, lower speed wireless connections, and lower-priced connections such as dial up [25, 26].The lack of access is problematic because of the rise in services such as video telehealth that require access to high speed reliable networks using adequately powered devices. |  |  |  |  |

**Study 10. Eruchalu et al. 2022**

Link: <https://pubmed.ncbi.nlm.nih.gov/35213441/>

| **Thematic barriers** | | | | |  |
| --- | --- | --- | --- | --- | --- |
| **Notes:** Characterized in previous literature as operational and logistical factors | | | | |  |
| **System level** | **Access** | **Proficiency** | **Intersectionality** | **Provider level** | **Patient level** |
| The novel finding that Black patients were more likely to use virtual surgical consultation during the Phase II period may reflect institutional efforts to expand digital health access for communities of color in addition to disproportionate access barriers to in-person care in this population  virtual visit use at this institution has remained closely associated with healthcare policy, and video visits continue to provide an important tool for care delivery. | Latinx patients were less likely to have a video vs audio visits relative to White patients  insurance type was not significantly associated with visit type  **Hispanic, low-income, have Medicaid insurance, and who speak languages other than English or Spanish** were less likely to have telemedicine visits during the Public Health Emergency and were more likely to face barriers to navigating online platforms.  Previous work has demonstrated that racial/ethnic minority and low-income patients are less likely to use the internet to obtain health information  Furthermore, patients who lack broadband internet access and those with lower digital literacy tend to have fewer telemedicine visits and are less likely to use patient portals to communicate with providers  , | Digital literacy  Decreased use of video virtual visits by patients who are Latinx, older, have lower education level, and who have non-English primary language may reflect lower digital literacy and digital access in these populations | Non-english speaking and race/Ethnicity  lower median household income, Medicaid insurance, and older age had decreased odds of virtual visit and video streaming use  ( in the study these disparities did not manifest in phase 1, but manifested later in phase 2. | Importantly, telemedicine use patterns continue to evolve during the pandemic. Virtual visit engagement is affected by patient and provider factors, which are influenced by the public health landscape as well as institutional, state, and national policies that govern healthcare delivery  For example, increased engagement of Black patients with virtual consultation and increased use of phone visits by Latinx patients may reflect personal or cultural preference, concerns about potential COVID-19 exposure during a clinic visit, or privacy concerns regarding video visits | Among patients using virtual visits, race/ethnicity and insurance type were not significant predictors of video use.  Additionally, patients may have privacy or security concerns related to discussing health information over a virtual platform |

| Thematic facilitators: | Thematic facilitators cont’d | Thematic facilitators cont’d |
| --- | --- | --- |
| This academic hospital implemented initiatives to reduce disparities in telemedicine access, address structural racism, and promote equity in patient-facing digital health platforms.  Thus, policies to expand digital access and promote digital literacy in vulnerable communities are urgently needed to reduce disparities in telemedicine engagement and promote high-quality surgical care delivery during virtual visits  broadband subscribership and data charge subsidies will help socioeconomically disadvantaged patients engage with mobile health platforms  Health systems can also collaborate with local and national governments to support policies that expand access to broadband internet and technological devices, which will enable participation in virtual visits. | Health systems should also purposefully enroll under-resourced patients in telemedicine platforms.  Digital literacy training as well as cultural and linguistic inclusivity in mobile health platform development will further help vulnerable patient populations engage with telemedicine.  Finally, expanding reimbursement incentives for virtual surgical consultation, including phone visits and patient portal communications, will promote equitable telemedicine access  Nevertheless, less is known regarding patient and provider perceptions of the quality of initial surgical consultation that is conducted through video or audio-only modalities. | Institutional policies that promote digital access and literacy should focus on facilitating effective use of video-enabled technology.  To promote equity, surgical providers can collaborate with their institutions and local policy makers to advocate for parity in reimbursement across visit modalities. Although providers and departments may become more selective in their use of digital health platforms as policies change, telemedicine will likely remain a valuable method of surgical care delivery. |

Study 11. Esper et al. 2021

Link: [https://pubmed.ncbi.nlm.nih.gov/33725762](https://pubmed.ncbi.nlm.nih.gov/33725762/)1

| **Thematic barriers** | | |  |  |
| --- | --- | --- | --- | --- |
| **Note:** | | |  |  |
| **Distance** | **Access** | **Intersectionality** | **Patient level** | **Provider- or clinic- level** |
| Patients who chose to participate in a telemedicine visit were from a farther average distance than our prepandemic in-person patient population. This is of particular interest in movement disorder patients, where the combination of disease progression often leads to limited mobility and subsequent challenges with in-person visits, as well as limited access to specialty care, is paramount | It is well documented in the literature that racial/ethnic minorities who are socioeconomically disadvantaged face significant barriers to receiving healthcare. Much of this disparity is thought to be due to lack of timely access to appropriate healthcare  Additional problems may include access to technology to conduct visits, including both devices and broadband or cellular capability. We anticipate further study to define the reasons for these disparities so they can be mitigated.  In our study, a higher percentage of patients with DBS chose to convert to telemedicine than patients without DBS. These findings may hint at the influence of disease severity, although we did not measure this variable directly. | Contrary to prior reports, age did not play a role in converting to a telemedicine visit  Men were more likely to transition to a telemedicine visit. This trend has been previously demonstrated in a number of publications  (particularly in developing countries where gender disparities may be more profound. It has been shown that some women face social barriers that may inhibit their participation in telemedicine) | Patient desire to wait for in-person appointment | lag in scheduling new patient visits to be able to accommodate existing patients of the practice  Lag due to need to minimize new testing (e.g., MRI) given social distancing restrictions  Inability to contact the patient or surrogate  Telemedicine adoption barrier due not scheduling procedural visits that were not able to be performed by telemedicine. |

**Study 12: Friedman et al. 2022**

[**https://pmc.ncbi.nlm.nih.gov/articles/PMC9256787/**](https://pmc.ncbi.nlm.nih.gov/articles/PMC9256787/)

| **Thematic barriers** | | | | |  |
| --- | --- | --- | --- | --- | --- |
| **Notes:** Our study was conducted over a longer time period (6 months) than other studies examining disparities in telehealth usage among the general population during the COVID-19 pandemic,which may have lessened the impact of the initial disparities seen at the sudden onset of the pandemic. ( a limitation of this body of work: short study period may not adequately reflect the magnitude of disparities in utilization) (review notes on study from phase one and two). | | | | |  |
| **System level** | **Access** | **Proficiency** | **Intersectionality** | Provider level | Patient level |
|  | **I**nternet [access] may have prevented telehealth usage, while use of public transit to attend in-person appointments, as well as fears of increased SARS-CoV-2 risk associated with the hospital setting may have resulted in overall lack of healthcare utilization among PwH during the pandemic**.**  hese racial and ethnic differences in telehealth use are likely due to structural factors including smart phone ownership (which is lower among Black patients) and lack of broadband access [30–32].  Other studies have found that patients with public insurance were less likely to use telemedicine |  | We found that older persons were more likely to attend virtual visits compared to younger patients, unlike some studies of non-HIV populations. Other studies also found that older persons were more likely to use telehealth than younger people  Potentially driven by news coverage highlighting the threat of COVID-19 particularly for older populations  preference of these same patients for telephone rather than video telehealth visits could be due to discomfort with the newer technology of video calls as opposed to the more traditional audio phone calls |  | It is also possible that PwH acquiring SARS-CoV-2 was a factor that drove telehealth use among PwH.  It may also be due to factors such as inability to obtain privacy at home or at work for a telehealth visit or cultural preference for in-person appointments |

| Thematic facilitators |  |  |
| --- | --- | --- |
| These financial and technological barriers can be addressed by interventions at the federal, state and local level  They may also be addressed by promoting and incorporating feedback regarding telehealth services among disadvantaged minorities |  |  |

**Study 13: Govier et al. 2022**

<https://www.ncbi.nlm.nih.gov/pmc/articles/PMC9012053/>

| **Thematic barriers** | | | | |
| --- | --- | --- | --- | --- |
| **Notes:**  social deprivation based on community social vulnerability such as minority status and language, and housing type and transportation were not associated with virtual care utilization (though they were associated with primary care utilization)    “it will be crucial to transition telehealth services from a crisis intervention tool to an equitable and sustainable system for providing proactive patient care” | | | | |
| **Access** |  |  |  |  |
| non-Hispanic White individuals generally enjoyed greater access to virtual care than non-White and Hispanic/Latino individuals during the COVID-19 pandemic—a particularly concerning finding given that racialized groups generally experience greater chronic disease burden than White and non-Hispanic populations and increased COVID-19 infection rates, both of which may necessitate increased access to care. |  |  |  |  |

| Thematic facilitators: | Thematic facilitators cont’d | Thematic facilitators cont’d |
| --- | --- | --- |
| Conducting targeted patient outreach and actively connecting with individuals and groups who experience barriers to care has been shown to improve access to and utilization of care: For example, in a study by Ospina-Pinillos et al. [58], participatory design methods were used to tailor the website of a virtual mental health clinic to improve outreach to Spanish-speaking individuals, which led to adequate acceptability levels in the website’s homepage, and triage, booking, and video visit systems for Spanish-speakers, and also enabled the clinic to identify the need for tailored assessment tools and greater integration with Spanish-speaking services and communities  **Racial/ethnic concordance** between patients and providers is associated with improved use of preventive services, satisfaction with care, patient-provider communication quality, and patient participation in care and decision-making  **In addition, evidence shows that clinicians from racialized groups are more likely to treat patients from racialized groups, including those who live in medically underserved and vulnerable areas**  Policymakers and health systems must purposefully devote financial and other resources to improving provider representativeness and dismantling racist and discriminatory practices including those that have resulted in a current provider supply that is more White and socioeconomically advantaged than the general U.S. population | A systematic review of interventions aimed at modifying the healthcare system to better outreach to and serve racialized groups and communities revealed that these interventions were associated with both improved processes of care delivery and reduced access disparities.  health systems must first be able to identify those experiencing barriers to care and find ways to create meaningful connections with them. This necessitates leveraging our current understanding of the multiple intersecting individual and community factors affecting access to care and addressing them in outreach materials and methods  **At a systems level, this means deconstructing current systems which are inherently racist, overtly discriminatory, and implicitly biased, and rebuilding them into more just and healing systems that are acceptable and comfortable for diverse patient populations.**  For virtual care, this also means conducting additional research on what constitutes effective and trustworthy outreach and communication to diverse populations  Designing culturally appropriate tools and technology that enable and improve access requires adaptations to systems predominantly designed for White, English-speaking individuals  Health systems can c**ollect and incorporate input on telehealth tools and technologies from racialized groups and those with limited English proficiency** [64], **as evidence indicates that cultural and linguistic tailoring can improve healthcare access and outcomes** | Data collection and user testing should be done in a participatory manner in which cultural adaptations, and knowledge and language translation are co-designed with patients and/or research participants  Health systems can also increase robust adoption of the National Culturally and Linguistically Appropriate Services Standards developed by the U.S. Department of Health and Human Services [68], which are intended to provide health workers and systems with a blueprint for developing equitable, understandable, respectful systems of care  One policy solution is to provide funding for broadband expansion in medically underserved communities. Several initiatives are underway to accomplish this: As part of thec, the Federal Communications Commission is launching the $3.2 billion Emergency Broadband Benefit program to help Americans with qualifying household incomes obtain high-speed internet  , a $100 million federal pilot program has been implemented to cover eligible costs of broadband connectivity, network equipment, and information services needed to provide connected care services to patients; and the COVID-19 Telehealth Program included $200 million in Congressional appropriations to help healthcare providers provide connected care to patients at their homes or in mobile locations |

**Study 14: Grefe et al 2023**

<https://pubmed.ncbi.nlm.nih.gov/37186764/>

| **Thematic barriers**  **Notes:** Characterized in previous literature as operational and logistical factors | | | | | |
| --- | --- | --- | --- | --- | --- |
|  |  |  |  |  |  |
| **Access** | **Policy** |  |  |  |  |
| Continued lower reimbursement for audio visits compared to video visits could negatively affect access to specialty services for children belonging to vulnerable groups  We did not have other socio-demographic variables that could have affected appointment outcomes transportation, internet access, or ability to access EHR portal. We did not collect data specifically for research purposes. | Continued lower reimbursement for audio visits compared to video visits could negatively affect access to specialty services for children belonging to vulnerable groups |  |  |  |  |

| Thematic facilitators: | Thematic facilitators cont’d | Thematic facilitators cont’d |
| --- | --- | --- |
| The best approach may be a shared decision-making model, wherein providers and families together decide on the best type of outpatient visit (audio vs. video vs. in-person) for the child given the type of care needed. Health systems caring for children should create structures and processes (such as clinic schedules and workflow) so that telehealth services remain an option for children for outpatient visits beyond the pandemic | Hence, flexibilities in the type of appointments offered (audio, video, or in-person) could reduce the strain on the health system by reducing canceled or missed appointments  Audio visits reduced the odds of missed appointments for all children; this association was more pronounced for children of Black race  Thus, telehealth (especially audio encounter) has the potential to address some of the existing inequities in healthcare access |  |

**Study 15: Jallow et al. 2023**

[**https://pmc.ncbi.nlm.nih.gov/articles/PMC9633105/**](https://pmc.ncbi.nlm.nih.gov/articles/PMC9633105/)

| **Thematic barriers** | | | | | |
| --- | --- | --- | --- | --- | --- |
| **Notes:** “As the literature describes, the unknown population may mask health care disparities among minority groups”  “Additionally, our results regarding ethnicity/race are specific to MedStar Health and do not necessarily reflect the ethnic representations in other health systems. Lastly, only synchronous teledermatology visits were included in this study, so the results from our study may not be generalizable to practices that utilize asynchronous or hybrid modalities of teledermatology.” | | | | | |
| **System-level** | **Access** | **Proficiency** | **Intersectionality** | Provider level | Patient level |
|  | Consistent with our hypothesis, these results emphasize the difficulties with maintaining access to technology in a group that is already considered to be disproportionately disadvantaged  Black and Hispanic patients are also less likely to own a computer or have broadband internet access at home. | The Asian patient population had the highest telehealth visit rate during and after the quarantine (73.44% and 18.09%, respectively) (Fig. 2).This result may have occurred because the English-speaking Asian population has higher rates of technology-based, technically skilled jobs than the rest.9 Our data could reflect this population's comfort and familiarity with the abrupt transition to telemedicine technology. | Alternatively, the ​​Medicaid insurance type had the lowest telehealth visit rate (excluding other/unknown) both during and after the quarantine  These findings suggest that the widespread use of telehealth instead of in-person visits during the pandemic decreased access to dermatologic care for the Medicaid insurance group. This finding is consistent with propositions in the literature suggesting disparities in access to telehealth among lower socioeconomic groups for whom this insurance type is intended  Virtual interpreters are a potential barrier for non-English speakers |  | non-White patients were more concerned with conversation privacy and inappropriate access to their data.  negative cultural perceptions of the telemedicine model may influence their distrust of the telehealth environment |

| Thematic facilitators: | Thematic facilitators cont’d | Thematic facilitators cont’d |
| --- | --- | --- |
| Our findings are inconsistent with other findings in the literature suggesting that non-English-speaking populations would have trouble connecting with telehealth care.8 One hypothesis is that these patients may find Medstar Health's teledermatology system relatively user-friendly. | With a well-integrated and user-friendly virtual visit platform, non-English speakers may benefit from teledermatology |  |

**Study 16: Kolb et al 2021**

[**https://pubmed.ncbi.nlm.nih.gov/33079014/**](https://pubmed.ncbi.nlm.nih.gov/33079014/)

| **Thematic barriers** | | | | |  |
| --- | --- | --- | --- | --- | --- |
| **Notes: Limitations:**  We acknowledge that these findings may vary depending on institution-specific telehealth plat- forms, registration, and scheduling processe | | | | |  |
| **System level** | **Access** | **Proficiency** | **Intersectionality** | Provider level | Patient level |
| email containing the instructions for the download and setup of the telehealth platform (or app), which had been diverted to spam folders. Instructions were initially not available in languages other than English,  For caregivers who were able to access the virtual waiting room but unable to connect to the provider within the app, the optimization of telehealth platforms is necessary  The required upload and download speeds set by the telehealth platform may play a large role in the ability of patients to connect with and maintain the connection to their providers  All major telehealth platforms use end-to-end encryption, which requires higher bandwidth and open network ports. CareConnect requires 3 Mbps for a stable 2-person (ie, physician and patient) connection but this may increase to 6 Mbps when a translator or other user is added into the call. Zoom  and many other videoconferencing technologies require less than 1 Mbps, which correlates with higher rates of successful videoconferencing, but they do not have end-to-end encryption  Under reduced manning, our division did not have sufficient man- power to directly contact each patient who missed a tele- health appointment  We also did not have enough portable workstations to allow all office personnel to work from home. For patients new to telehealth, setting up the app with the patient can take 20 minutes or longer; therefore, it was not feasible to attempt the visit that day, even if families could be contacted. Since patients could be rescheduled within 24 to 72 hours in nearly all cases, it was likely not a significant problem for most families  It is noteworthy that even in a large tertiary care facility, there was insufficient manpower and portable workstations to optimize a large- scale telehealth practice. This problem would likely be com- pounded in smaller practices.  We also found that our technical support capability was initially overwhelmed by the large number of patients who did not receive or were unable to use the instructions. | education gaps and the inability to afford devices and Wi-Fi capability within the home. Further study into the cause of these potential socioeconomic and ethnic disparities is warranted.  one major Internet service provider in the United States guarantees only upload speeds of 768 kb/s for their least expensive Internet plan. This may significantly disadvantaged families with lower budgets for home Internet service.  Other identified causes of technological failure include multiple devices using the Internet simultaneously, poor Wi- Fi connection, and firewall or antivirus software delaying, interrupting, or denying telehealth traffic. | Technical issues were the most common causes  For fam lies with large education or technologic gaps, technical sup- port assistance may be necessary for success | Five percent of patients were non–English speaking and sched- uled without translator support  caregivers who spoke only Spanish were initially scheduled without translator support, which was later incorporated into the scheduling workflow on telehealth day  Minorities and patients with public insurance represented 53.6% and 61.9% of missed appointments, respectively.  Three of 6 patients who missed multi- ple telehealth appointments were Hispanic or Latino, which may support the concern that language barriers were a frequent cause of MA  Disparities in access to telehealth care may exist for patients with public insurance or minority groups with language or technology barriers.. |  | Distance from clinic  appointments before 9 AM and after 2 PM  New patients from minority groups with public insurance. New patients are more likely to miss telehealth appointment relative to establish patients  some caregivers were unable to understand the instructions for using the application. |

| Thematic facilitators: | Thematic facilitators cont’d | Thematic facilitators cont’d |
| --- | --- | --- |
| Education mate- rials regarding early application set up in advance of the appointment and the technological aspects of telehealth should be available to families to promote success.  A backup platform for families incorporating a web link sent directly via text message, which will automatically connect to the patient waiting room and requires only 2 Mbps, may help to decrease the technology-driven issues in some families. T | We recently upgraded our telehealth package to allow speeds as low as 2 Mbps when using a computer, but speeds are unchanged for phone and tablet devices  Providers and families should be given troubleshooting tips in their native language, including the use of hard-wired connections in lieu of Wi-Fi when possible, reducing firewall gateway settings to the lowest level that will allow peer-to-peer applications, using Wi-Fi with a minimum of 3 bars, moving devices closer to the router to avoid microwave and interference from other 2.4 GHz devices, and disconnecting other devices from the Internet during the telehealth visit  Patient reminders via both email and text messaging are encouraged to prevent the loss of reminder emails to spam. | .  Other institutions have been able to proactively contact telehealth patients who do not check in on time  Encouragement of set- ting the application up at the time of appointment scheduling may result in longer call times but decreased technical sup- port resources and late and MA rates.  At the onset of large-scale telehealth operations, families were sent an email to prepare them for the visit with instructions to assist with download- ing and setting up the application.  We later incorporated text reminders at 72 hours and 30 minutes prior to the appoint- ment to reduce MAs. Some studies showed decreased rates of missed office appointments when patients were scheduled within 2 to 3 weeks  concise education materials on the technical aspects of telehealth in the care- giver’s primary language may help to improve MA rates.  Optimization of telehealth processes by providing patient reminders, application setup at the time of appointment scheduling, determining the need for translator assistance, and reducing the required upload and download speeds for tele- health platforms may significantly reduce the rates of MA and conversion to other communication.  When considering telehealth expansion, an assessment of institutional capability is critical, as robust technical support capability may be necessary to address shortfalls in education.  . |

**Study 17: Kummer et al. 2022**

[**https://pmc.ncbi.nlm.nih.gov/articles/PMC8873082/#:~:text=Results,up%20during%20the%20COVID%20period.**](https://pmc.ncbi.nlm.nih.gov/articles/PMC8873082/#:~:text=Results,up%20during%20the%20COVID%20period.)

| **Thematic barriers** | | | | | |
| --- | --- | --- | --- | --- | --- |
| **Notes: “**Concerningly, technology access gaps persisted during the early and middle phases of the COVID-19 crisis, with patients from disadvantaged populations continuing to demonstrate poor utilization of both telehealth”  “Because we could not collect information relating to ED visits or hospitalizations at institutions other than ours from our clinical data warehouse, the rates of both these outcomes may have been understated.” | | | | | |
| **System level** | **Access** | **Proficiency** | **Intersectionality** | Provider level | Patient level |
| **Because many trainees were deployed to inpatient services for COVID-related care during the first several months of the COVID sur**ge**and only returned to in-person office visits in June 2020, Medicaid-insured patients were likely unable to find available providers during the initial 3 months of the COVID pandemic between March and May 2020.**  Hospital-administered and faculty practice clinics may have differed in the degree of TN platform on- boarding and technical support that was provided to patients and their caregivers to encourage familiarity with TN care. This may have driven some of the decreased TN utilization among Medicaid-insured patients.  Additionally, the lack of integrated translator services in our institutional TN platform during the early COVID-19 pandemic period could have been the cause of low utilization among non- English speaking populations. While translator services were available during this period, they were not integrated into the official institutional TN platform and required providers to access the services via a separate but concurrent telephone communication | inter-related digital and socioeconomic inequalities in the US healthcare system that clearly preceded the COVID-19 crisis  Our analysis also lacked granular sociodemographic characteristics such as patient domiciled status, access to caregivers or home assistance, and reliable access to Wi-Fi, smartphones, or computers | Technology literacy | Groups with lower telemedicine utilization were older, non-White, non-English speaking, non-commercially insured, have greater medical comorbidity,ive in areas with lower household incomes, and seek care through ED visits or hospital admissions  In another comparable study, patients who had telephone visits instead of TN visits were more likely to be older, non- commercially insured than patients evaluated by TN, with a pediatric subgroup being more likely to be non-English speaking  This digital divide has been shown to disproportionately affect the most disadvantaged patients in society, including ethnic minority, (32) elderly, (33, 34) economically disadvantaged, ) non-English speaking, and low health literacy patient groups (36).  This return to office visit care, combined with a preference for office over TN care, may also partially explain why Medicaid-insured patients were significantly less likely to be lost to follow-up during the COVID period.  we found that patients who were lost to follow-up were more likely to have a preferred language other than English and Medicaid insurance. Well-documented associations between limited health care access and reduced English proficiency, (42–46) Medicare or Medicaid insurance, and low income (47) may explain some of these commonalities.  **independently associated with loss of global access to care, and that patients with Medicaid insurance were in fact less likely than commercially-insured patients to be lost to follow-up during the pandemic** | Providers’ variable technology preferences and beliefs about telemedicine care may also have significantly influenced the degree of TN utilization.  Our analysis also lacked granular sociodemographic characteristics such as providers’ attitudes toward TN, | Patient education level |

**Study 18: Lin C et al. 2023**

[**https://pubmed.ncbi.nlm.nih.gov/36609783/**](https://pubmed.ncbi.nlm.nih.gov/36609783/)

| **Thematic barriers** | | | | |  |
| --- | --- | --- | --- | --- | --- |
| **Notes: “**The low telemedicine utilization rate (~ 5%) reported in the survey may suggest only a supplementary role of telemedicine in mental health service provision during the first year of COVID. I”  “This finding can be explained by the Anderson Behavioral Model of Health Service, that a person needs factors, e.g., pre-existing health conditions, are predictive of their health service utilization (Anderson, 1995)” | | | | |  |
| **Geographic** | **Access** | **Proficiency** | **Intersectionality** | Provider level | Patient level |
| A surprising finding is that residents in large metropolitan areas utilized less mental health treatment than those in small metropolitan areas. We speculate the reason for this phenomenon being the COVID crisis was initially concentrated in urban areas before it gradually spread to suburbs and then rural areas (Matheson et al., 2020), so urban residents, as compared to those in suburbs, might have avoided in-person healthcare services due to the fear of COVID exposure during the first year of the pandemic. | Pre-exisiting low accessibility and affordability of mental health utilization was exacerbated by stay at home order  the residential segregation and inequitable distribution of health-related resources (Yelton et al., 2022)  With enhanced broadband coverage, telemedicine could be a viable approach to increase access and alleviate mental health treatment disparity in rural areas  This study also suggested the unmet mental health service needs among the male population, full-time employees, patients with insufficient insurance coverage, and patients with co-occurring SUDs. |  | Older aga: , possibly due to their greater difficulty adapting to internet technology (Lam et al., 2020; Ridout et al., 2021).  There may be | mental health treatment, the gap in outpatient mental health treatment in rural areas identified in this study warrants attention and targeted approaches to address rural-specific service barriers, including the limited availability of specialty mental health care, lack of trained mental health providers, and underdeveloped care coordination in rural areas | , the unmet mental health needs among Asian and Hispanic populations might be attributable to negative cultural beliefs about mental health and misconceptions of pharmaceutical treatment for mental illness  n addition, some older patients conceived telemedicine as incomplete or less rewarding compared to traditional in-person visits  this study found that full-time employees utilized mental health treatment and services at a lower level than part-time employed or unemployed populations, with other covariates (including insurance and income) being controlled. Supported by literature (Dewa, 2014) and respondents’ reported reasons for not receiving treatment, full-time employees’ mental health treatment seeking may be deterred by **workplace stigma towards mental illness** and **fear of damaging their career if disease status is inadvertently disclosed**  Higher levels of mental health treatment and additional telemedicine service utilization were observed among respondents who experienced major depressive episodes and/or serious psychological distress  **However, such an association was not observed among respondents with co-existing mental illness and SUD** |

| Thematic facilitators: | Thematic facilitators cont’d | Thematic facilitators cont’d |
| --- | --- | --- |
| This finding calls for a better understanding of diverse cultural groups’ concerns and preferences of mental health treatment, with which culturally competent strategies (such as ethnic matching and culturally tailored languages in assessment and counseling) can be devised to engage race/minority patients with mental illnesses in treatment | Therefore, compensated high-speed internet and technical assistance are necessary but not sufficient to bridge older patients to their needed mental health care; a thorough understanding of context-specific issues faced by older patients during telemedicine is warranted to develop strategies to promote equitable telemedicine-delivered services for vulnerable older patient | **Employers should provide a supportive environment and flexible work hours to encourage their employees’ mental health service utilization** |

**Study 19: Mueller et al. 2022**

[**https://www.ncbi.nlm.nih.gov/pmc/articles/PMC9015206/**](https://www.ncbi.nlm.nih.gov/pmc/articles/PMC9015206/)

| **Thematic barriers** | | | | |  |
| --- | --- | --- | --- | --- | --- |
| **Notes:** “a shutdown period (March 23, 2020–May 23, 2020), when nonessential in-person visits were strictly limited, and a reopening period (May 23, 2020–September 23, 2020), when restrictions were relaxed and in-person visits were available.**”**  **“**Previous research examining sociodemographic factors and use of telehealth demonstrate a context-dependent relationship”  “Despite the establishment of parity for telephone visits by the Centers for Medicare and Medicaid Services (CMS), telephone visits are associated with lower patient satisfaction and inferior communication of medical information in comparison with video visits.20,27,44 For patients with limited English proficiency, telephone visits are especially challenging.27 Visual information may be particularly important for providers caring for pain patients because nonverbal cues can provide insight into the individual pain experience.”  “Second, our data are from a single large health system and patients could have had encounters at a different health system. Third, our urban study population may limit the study's generalizability to rural areas where broadband Internet is not as readily available.” | | | | |  |
| **Systemic** | **Access** | **Proficiency** | **Intersectionality** | Provider level | Patient level |
| Historical mistreatment and current systemic inequalities may contribute to a wariness of technological innovations by the medical community. | Internet access  In addition, people of lower socioeconomic status may have reduced access to a private space at work and home. An audio-only telehealth visit might afford greater privacy or flexibility and therefore be preferable for some patient | digital literacy | Patients who were older, publicly insured, and identified as Black or Hispanic were overrepresented in the telephone visit group during shutdown and the in-person group during reopening.  Finally, chronic medical conditions that are prevalent in people who are Black or Hispanic may have also an increased need for medical care.  Our study supports the importance of distinguishing between telephone and video visits.21,47 During shutdown, older patients, patients identified as Black and Hispanic, and publicly insured patients were significantly more likely to use telephone vs video than younger, White, privately insured patients. | provider–patient relationships  In addition, there is evidence that face-to-face contact may play a more important role in therapeutic alliance and rapport building for race and ethnic minority individuals. | provider–patient relationships  Previous work has demonstrated that patients of minority race and ethnicity and those of lower socioeconomic status are more likely to experience severe pain,(**disease severity**)  **. The differential impact of COVID-19 on minorities and those with a lower socioeconomic status45 may have led to exacerbations of pain, increasing the likelihood of a medical visit** |

| Thematic facilitators: | Thematic facilitators cont’d | Thematic facilitators cont’d |
| --- | --- | --- |
| Finally, a video visit during shutdown was a strong independent predictor of continued video use after in-person visits returned | This is likely due to the development and implementation of outreach efforts that took time to execute and included assisting patients with portal activation and offering video visits through multiple platforms. | The strong relationship between video visit group during shutdown and continued video use during reopening aligns with the popularity of video visits and their continued availability despite a decreased need to social distance due to declining rates of COVID-19 infection. For patients with pain that limits mobility, TH may offer a significant benefit.  This important information can be used to guide the formation and implementation of inclusive and flexible telehealth services and policies to prevent widening of existing disparities for patients with pain. |

**Study 20: Neeman et al. 2021**

<https://pmc.ncbi.nlm.nih.gov/articles/PMC9067360/>

| **Thematic barriers** | | |  |
| --- | --- | --- | --- |
| **Notes:** “Kaiser Permanente has promoted equity, inclusion, and diversity throughout its history, and regularly embeds mechanisms to identify and eliminate inequities in its operations,31 and its patient population reflects the racial and ethnic diversity of the population it serves.15 Thus, it is possible that our findings may in fact under-represent the severity of disparities in utilization of telehealth and secure message by patients with cancer across the nation” | | |  |
| **Policy** | **Intersectionality** | Provider level | Patient level |
| Regulation on reimbursement for telephone and video visits, by contrast, continues to evolve since the COVID-19 pandemic started, but in certain practice settings, these types of visits are reimbursed at a considerably lower rate than office visits  **regarding payment for telehealth by the Centers for Medicare and Medicaid are still considered temporary, and coverage and compensation rates for telehealth services by private insurers are governed by individual state regulations**  Additionally, since telephone visits are reimbursed at a much lower rate than video visits in most settings, some providers may not offer telephone **visits to their patients and, as such, inadvertently exclude access to telehealth for those who cannot use video**. Thus, it is concerning that this lack of payment parity combined with the disparities in use of video visits and secure messages reported herein may disproportionately negatively affect clinical outcomes and quality of life for disadvantaged populations. | Possible disparities existed with video visit and SM use by age, insurance plan, language, race, ethnicity, marital status, comorbidities, and sex. | This lack of payment parity between methods of care delivery may affect provider choices in how frequently to see each patient and by which method(s), and/or discourage providers from attending to secure messages in a timely and thoughtful manner, thus potentially leading to insufficient medical supervision with less use of telehealth and secure messages, or increased exposures to COVID-19 and other pathogens with office visits  Additionally, since telephone visits are reimbursed at a much lower rate than video visits in most settings, some providers may not offer telephone visits to their patients and, as such, inadvertently exclude access to telehealth for those who cannot use video. Thus, it is concerning that this lack of payment parity combined with the disparities in use of video visits and secure messages reported herein may disproportionately negatively affect clinical outcomes and quality of life for disadvantaged populations. | Although our study was not designed to answer this question, it is hypothesized that in our health care system, it is the provider or the system that commonly decides between office or telehealth visit, but it is the patient or patient characteristics that influence the choice between telephone and video visits. |

| Thematic facilitators: | Thematic facilitators cont’d | Thematic facilitators cont’d |
| --- | --- | --- |
| **These include the rapid and sustained uptake of telehealth and secure messages as shown herein, high patient40-42 and provider16 satisfaction with telehealth, the lower-than-desired COVID-19 vaccination rates and ongoing infection waves,43 dispersion of** **broadband internet technology over time, and the recent recommendations for policymakers to extend current telehealth-related regulatory expansions.44 Indeed, ASCO has advocated for telemedicine policies to remain in place after the public health emergency ends.** | including live interpreter services, education and tech support for patients and caregivers, working with local communities to identify public facilities with broadband access for patients to use, and teaching clinicians communication skills specific to telehealth  understanding preferences of patients and caregivers from various demographic groups regarding the use of these technologies, and testing interventions to improve access and equity in telehealth and secure messages | and an extensive patient survey of patients' preferences and barriers relating to telehealth and secure messaging is underway. |
